# Dynamic Risk From Mexican Wolves and Mountain Lions Influences Elk Foraging Behavior

**DOI:** 10.1002/ece3.72520

**Published:** 2025-12-17

**Authors:** Julia E. Olson, Cara J. Thompson, Zachary J. Farley, Samuel I. Martinez, Scott T. Boyle, Nicole M. Tatman, James C. DeVos, Stewart D. Liley, James W. Cain

**Affiliations:** ^1^ New Mexico State University Department of Fish, Wildlife, and Conservation Ecology Las Cruces New Mexico USA; ^2^ New Mexico Department of Game and Fish Santa Fe New Mexico USA; ^3^ Arizona Game and Fish Department 5000 W Carefree Hwy Phoenix Arizona USA; ^4^ U.S. Geological Survey, New Mexico Cooperative Fish and Wildlife Research Unit New Mexico State University, Department of Fish, Wildlife, and Conservation Ecology Las Cruces New Mexico USA

**Keywords:** behavioral trade‐offs, foraging behavior, multiple predators, non‐consumptive effects, predation risk, vigilance categories

## Abstract

Foraging time is a major component of ungulate activity budgets but can be limited by anti‐predator behaviors (e.g., vigilance). Multitasking can reduce the nutritional costs of vigilance under heightened predation risk, but this may depend on the response of prey to risk from multiple predators across a complex spatiotemporal landscape. Mexican gray wolves ( *Canis lupus baileyi* ) and mountain lions ( *Puma concolor* ) are primary predators for elk ( *Cervus canadensis* ) in the Mexican wolf experimental population area in east‐central Arizona and west‐central New Mexico. We observed elk foraging across varying levels of wolf risk throughout all seasons and diel periods to quantify proportions of foraging, intense vigilance, and multitasking at the individual and herd levels. We quantified encounter and kill risk from Mexican wolves and mountain lions using habitat selection functions and utilization distributions. We modeled elk behaviors as functions of predicted risk for both predators in addition to temporal and environmental covariates and accounted for human presence. Our results indicate that individual elk reduced foraging in areas with higher predicted risk from Mexican wolves or mountain lions and increased intense vigilance and multitasking in areas with higher wolf risk. A reduction in the proportion of bedded elk in the herd during all diel periods under increased wolf risk supports previous findings. These results also suggest that elk compensate for higher intense vigilance and reduced foraging during foraging bouts by increasing cumulative foraging bouts per day at the cost of resting. Additionally, the probability of multitasking for individuals depended on an interaction between short‐ and long‐term wolf risk, and the likelihood of intense vigilance was highest under the greatest combined spatial and temporal risk from wolves. This research provides insight into the fine‐scale and complex behavioral responses of elk to their primary predators and implies a need for researchers to consider these non‐consumptive effects in future studies of predator–prey dynamics.

## Graphical abstract

We observed elk foraging across a wolf risk gradient throughout all seasons and diel periods to quantify proportions of foraging, intense vigilance, and multitasking at the individual and herd levels. We quantified encounter and kill risk from Mexican wolves and mountain lions using habitat selection functions and utilization distributions. We modeled elk behaviors as functions of predicted risk for both predators in addition to temporal and environmental covariates and accounted for human presence.

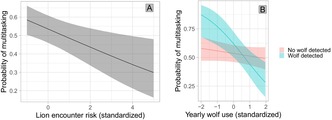

## 1 Introduction

Nutritional limitations can lead to changes in body condition, susceptibility to parasites, and reduced reproductive success and survival probability (Bukovinszky et al. 2009; Stephenson et al. 2020). Individual body condition in ungulates can scale up to influence population dynamics by affecting pregnancy rates or the birthweight and survival of neonates. For example, adult female bighorn sheep ( *Ovis canadensis* ) with higher levels of body fat had higher probabilities of pregnancy and overwinter survival (Stephenson et al. 2020). Additionally, the mean body fat of lactating females was positively correlated with increased population growth rates (Stephenson et al. 2020). Similarly, female elk ( *Cervus canadensis* ) in better nutritional condition over the prior breeding season gave birth to calves with greater body weights, a positive predictor of overwinter survival (Cook et al. 2004). These nutritional limitations on individual fitness are primarily a result of the interplay between forage conditions and foraging behavior of ungulates.

Wild ungulates spend the majority of their time foraging to meet their energetic and nutritional requirements (Hamel and Côté 2008; Roberts et al. 2017). Optimal foraging theory (OFT; Emlen 1966, MacArthur and Pianka 1966) suggests that ungulates will continue to forage in a patch until the energy gained from forage no longer outweighs the energy expended to collect it. However, ungulates must also balance foraging demands with other behaviors such as avoiding predation and conspecific competition. As prey, ungulates may choose to forage in areas with lower quality food or reduce foraging time to reduce predation risk (Brown et al. 1999; Christianson and Creel 2008; Cain III et al. 2024). Prey may also use high‐risk areas at times of low risk based on activity periods of predators (Kohl et al. 2018; Ganz et al. 2024).

Direct effects of predation (i.e., consumption of prey by predators) may be exacerbated by non‐lethal, indirect effects which may have significant ramifications for individual fitness and population dynamics, ranging from decreased body condition and reproductive rates to changes in population growth rates (Lima 1998; Brown et al. 1999; Creel et al. 2007). In systems with low predation rates but a large proportion of the prey population displaying anti‐predator behaviors, non‐lethal effects may outweigh the direct effects of predation (Dwinnell et al. 2019). Anti‐predator responses are related to predator proximity or behavior, surrounding habitat characteristics, or behavior of nearby conspecifics (Liley and Creel 2008). Under the flight initiation distance hypothesis, prey weigh the decision to engage in anti‐predator behaviors with their costs based on the proximity, behavior, and hunting strategy of predators (Ydenberg and Dill 1986). According to the risky places and risky times hypothesis, prey can mitigate the trade‐off between anti‐predator behavior and foraging by altering their behavior based on perceived risk levels (Lima and Dill 1990). For example, female elk in Yellowstone National Park increased vigilance in risky areas if wolves ( *Canis lupus* ) were actively present (Winnie and Creel 2007).

Prey vigilance can enhance detection of predators (Bøving and Post 1997; Skinner and Hunter 1998; Brown et al. 1999). Increased vigilance, however, can reduce foraging time, potentially limiting food intake and thus compromising fitness (Lima 1998), though a recent study on mountain goats ( *Oreamnos americanus* ) found no relationship between vigilance and individual fitness (Déry et al. 2024). Ungulate species, which in nearly all cases co‐evolved with predators and are adapted to predation risk, can reduce the potential foraging costs of vigilance by multitasking, in which animals process food while searching their environment for predators (Fortin, Boyce, and Merrill 2004). This is in contrast to “intense vigilance” where chewing ceases in order to increase detection of predators by devoting all senses to scanning the environment (Blanchard and Fritz 2007; Yiu et al. 2021). Multitasking, however, does not entirely eliminate the costs of vigilance. Fortin, Boyce, Merrill, and Fryxell (2004) reported that although elk and bison ( *Bison bison* ) employed multitasking, individuals reduced their bite rate by 20% as vigilance increased. Higher levels of predation risk may require more time being intensely vigilant, leading to a greater disruption to prey foraging behavior. However, the extent to which increasing vigilance in high‐risk areas affects nutritional state likely depends on forage conditions, as ungulates foraging in high‐risk areas with high‐quality forage may not be nutritionally limited (Brown et al. 1999), and evidence for changes to ungulate population dynamics mediated by non‐lethal predation risk is highly limited.

The distribution of risk on the landscape may also affect vigilance. The risk allocation hypothesis predicts that prey consistently exposed to high levels of predation risk will be less vigilant at safer times than prey exposed to relatively low levels of risk, while prey in the low‐risk environment will be more vigilant at the riskiest times than prey in the high‐risk environment (Lima and Bednekoff 1999). This strategy can resolve the dilemma of prey foraging under greater long‐term densities of predators, where animals are unable to engage in heightened anti‐predator behavior in response to every risk signal. Creel et al. (2008) reported evidence supporting this hypothesis, with overall lower and more variable wolf risk resulting in higher vigilance in elk than overall higher but less variable wolf risk. This is contrary to the risky places/risky times hypothesis, which predicts that the elk exposed to greater overall risk should be more vigilant (Dröge et al. 2017). Additionally, predator characteristics including pack size may influence vigilance, with elk exposed to larger wolf packs displaying elevated vigilance (Liley and Creel 2008; Farley et al. 2024).

Prey forage in larger groups to mitigate associated costs of vigilance by employing collective vigilance or because of the dilution of risk for individuals in larger groups (Bøving and Post 1997; Skinner and Hunter 1998; Childress and Lung 2003; Périquet et al. 2012). Larger groups, however, can be more detectable to predators, and increased intraspecific competition can reduce forage intake (Lima and Dill 1990; Laundré et al. 2001; Creel and Winnie 2005). Animals located on the periphery of the herd may be more vigilant because they may encounter an attacking predator first (Skinner and Hunter 1998; Burger et al. 2000; Dalmau et al. 2010). An individual's ability to make the tradeoff between vigilance and feeding also depends on their body condition, age, and reproductive state. During summer, female elk with calves tend to show higher levels of vigilance than those without calves, indicating that the greater risk of predation to calves can outweigh the cost of reduced foraging time for lactating females (Skinner and Hunter 1998; St‐Louis and Côté 2012; Yiu et al. 2021). The physical environment also plays a role in perceived predation risk, which may be influenced by escape capacity or cover (Altendorf and Laundre 2001; Liley and Creel 2008). Therefore, a combination of factors including prey, predator, and environmental characteristics likely influences prey vigilance levels (Liley and Creel 2008).

Mexican gray wolves ( *Canis lupus baileyi* ; hereafter Mexican wolves) were extirpated from the southwestern United States in the mid‐1900s and reintroduced to New Mexico and Arizona in 1998 (Parsons 1998). As of December 2023, the population had a minimum of 257 wolves which comprised 60 packs (≥ 2 individuals; USFWS 2024). Elk are the primary prey for Mexican wolves, comprising up to 80% of their diet in the summer (Smith et al. 2023; Martinez 2024) and variation in wolf density and an expanding population results in a landscape with patches of high and low predation risk. Mountain lions ( *Puma concolor* ) also contribute significantly to predation risk for elk in this region. Few studies have investigated the impact that Mexican wolves have on the foraging behavior of elk (Farley et al. 2024), which is a major knowledge gap as Mexican wolf numbers increase. Similarly, there has been little research on the effects of mountain lion predation risk on elk in the Southwest (though see Boyle 2025), although studies on other mountain lion populations sympatric with elk and wolves indicate that mountain lions could account for a greater proportion of adult and calf mortalities than wolves, especially in areas where wolves are recovering (Eacker et al. 2016). In systems where two predators occupy similar dietary niches, their presence may have an additive effect on risk‐avoidance behavior in prey, or prey may only respond significantly to risk by their primary predator (Prugh et al. 2019). Prey may also select habitat to spatially and temporally avoid areas of high risk from multiple predators, or they may primarily avoid areas with the most predictable risk (Kohl et al. 2019). The response of elk to risk posed by mountain lions, a stalking predator, may be more related to habitat characteristics that provide stalking cover whereas risk created by Mexican wolves, a coursing predator, may be more directly related to predator presence and density. Humans are also a significant predator for elk and may outweigh the lethal and non‐lethal effects of non‐human predators during hunting seasons (Proffitt et al. 2009; Ganz et al. 2024). Thus, predictable risk from elk hunters likely influences seasonal elk anti‐predator behavior (Proffitt et al. 2009).

We investigated the foraging ecology and behavior of elk in response to predation risk from Mexican wolves and mountain lions in New Mexico and Arizona. We hypothesized that elk would increase foraging and reduce intense vigilance at times of low short‐term risk in areas with generally higher long‐term predation risk due to risk allocation. Additionally, we predicted a contrasting effect of human density in hunting and non‐hunting seasons. During hunting season, we expected foraging time would be highest in areas with lowest human density while during non‐hunting seasons elk would increase foraging in areas with moderate human density due to a refugia effect from predators. We also hypothesized that when forage was limited in winter and early summer, elk would allocate more time to foraging and would use multitasking more than intense vigilance to compensate for reduced nutritional intake. However, we predicted elk would trade off foraging to increase intense vigilance during monsoon season as the better forage conditions would allow elk to allocate a greater proportion of their activity budget to vigilance at a time when vulnerable calves are present in the herd. We also predicted that elk would reduce vigilance in larger herds but increase traveling and step rate due to competition.

## 2 Study Area

**FIGURE 1:**
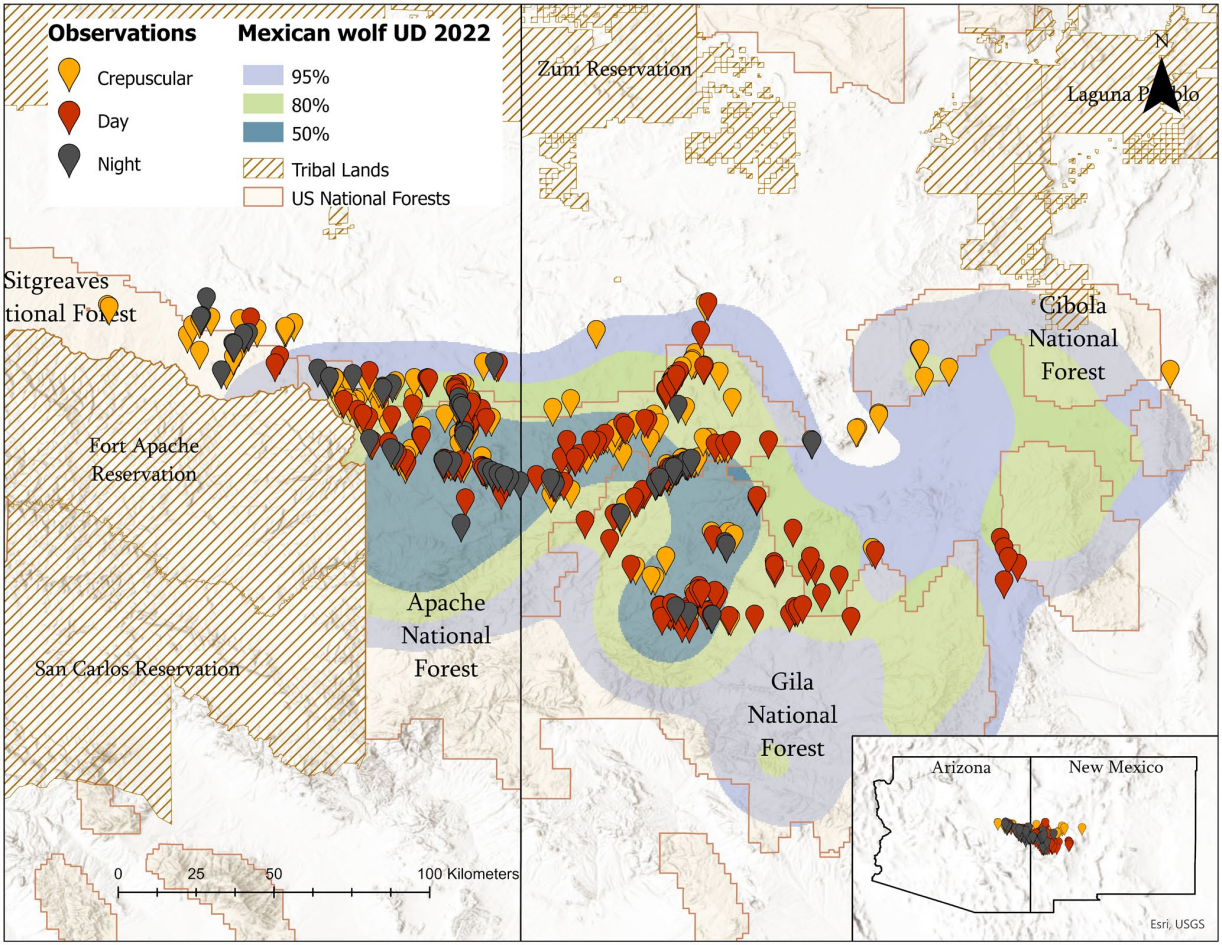
Observation locations and land ownership within the study area in 2022–2023. Red indicates observations that occurred during the day; yellow at dusk (within 1 h before or after sunset) or dawn (within 1 h before or after sunrise); and black at night. The Mexican gray wolf population utilization distribution (UD) for 2022 in Arizona and New Mexico is shown with 50%, 80%, and 95% isopleths. Data from tribal lands are excluded from figures based on an agreement between local tribes and USFWS.

We studied elk behavior in the Mexican Wolf Experimental Population Area (MWEPA) in New Mexico and Arizona. Our study area covered ~30,000 km^2^ and includes the Apache‐Sitgreaves, Gila, and Cibola National Forests along with eight wilderness areas and one primitive area and is bordered to the west by the White Mountain (Fort Apache) and San Carlos Apache reservations and to the north, east, and south by private, state‐trust, and Bureau of Land Management (BLM) lands (Figure 1). Several small towns border or fall within the study area and livestock grazing and human recreation, including big game hunting, are common.

Elevation ranges from 1700 m in the desert lowlands to 3480 m in the mountains (USFWS 1996). At low elevations (< 1975 m), semi‐arid grasslands comprised of diverse native grass species, Chihuahuan desert scrub including creosote bush ( *Larrea divaricata* ) and mesquite ( *Prosopis glandulosa* ), and patches of Madrean evergreen woodlands of various *Pinus*, *Juniperus*, and *Quercus* species dominate (USFWS 1996). At mid‐range elevations (1975–2675 m) conifer forests including juniper (*Juniperus* spp.) and piñon pine ( *Pinus cembroides* ) dominate. Ponderosa pine forests ( *Pinus ponderosa* ) mixed with aspen ( *Populus tremuloides* ) and fir (*Pseudotsuga menziesii* and *Abies* spp.) occur at high elevations (> 2675 m).

Weather is mild and cool at higher elevations, with mean temperatures of 8°C (min.) to 23°C (max.) in the summer and −8°C (min.) to 5°C (max.) in the winter (PRISM 2021). The majority of precipitation occurs during monsoon season (July to September), and most snowfall occurs in the winter between December and March (USFWS 1996). Mean total precipitation is 68.7 cm at high elevations with an annual snowfall of 134.4–252 cm (Alpine and Greer, AZ; PRISM 2021; WRCC 2021). Lower elevations are warm with mean temperatures of 12°C (min.) to 30°C (max.) in the summer and −6°C (min.) to 11°C (max.) in the winter (PRISM 2021). Mean total precipitation at low elevations is 36.7 cm with annual snowfall of 32.2–55.8 cm (Show Low Airport, AZ and Hood Ranger Station, NM; PRISM 2021; WRCC 2021).

Large carnivores include Mexican gray wolves, mountain lions, black bears ( *Ursus americanus* ), bobcats ( *Lynx rufus* ), and coyotes ( *Canis latrans* ). Ungulate prey includes elk, mule deer ( *Odocoileus hemionus* ), pronghorn ( *Antilocapra americana* ), collared peccary ( *Pecari tajacu* ), Coues deer ( *Odocoileus virginianus couesi* ), and bighorn sheep (USFWS 1996).

## 3 Methods

### 3.1 Observation Data Collection

During 2019–2023, we captured adult female elk by helicopter net‐gunning, or darting from the ground using BAM (Butorphanol‐Azaparone‐Medetomidine) according to dosages recommended by Wolfe et al. (2014) and fitted elk with GPS‐Iridium collars (ATS, model G5‐2D, Isanti, MN). We captured neonatal elk opportunistically from the ground or helicopter and used partition movement code to identify potential parturition events based on GPS collar data from adult female elk during calving season (Vore and Schmidt 2001). We fitted neonates with breakaway Globalstar GPS collars (Vectronics, VERTEX MINI; Berlin, Germany) and VHF ear tag transmitters (ATS, M3430, 23 g; Isanti, MN). During 2021–2023, we observed actively foraging male and female elk and herds while traveling along roads and searching from vantage points throughout the study area. We did not target marked animals for observation but located herds by driving or hiking throughout the study area. We stratified observations across wolf density gradients and temporal scales (seasonal and diel periods). We defined a herd as all animals within 100 m of the next (Bøving and Post 1997; Fortin, Boyce, Merrill, and Fryxell 2004; Proffitt et al. 2009), and considered herds unique if we observed them more than 1 km apart on the same day or on different days (Liley and Creel 2008). We observed elk using high‐powered spotting scopes (20–60 × 85 magnification) to avoid altering the animals' behavior. Upon arriving at an observation site, if any elk appeared to be looking at field personnel or the vehicle, we waited to begin observations until they resumed foraging.

We collected behavioral data using continuous focal sampling and instantaneous scan sampling (ISS) of a herd (Altmann 1974). We selected focal individuals by generating a random cardinal quadrant (NW, NE, SW, and SE) and choosing a foraging and observable adult elk from within that quadrant of the herd. If we collected multiple focal observations from a single herd, we selected elk from a different quadrant than the first to reduce the likelihood of observing the same individual twice. We used Survey123 (Esri, Redlands, CA) to simultaneously count the steps the individual took and record the behaviors they engaged in and the times that they changed behaviors. When possible, pairs of field personnel conducted surveys with one person observing elk and verbally relaying information to a second person acting as the data recorder. Behaviors recorded included foraging, multitasking, intense vigilance, traveling, nursing, and bedded (Supporting Information S1: Table A1). If our view of an individual became obstructed during the observation or it was impossible to distinguish vigilance from multitasking, we recorded the behavior as unknown and excluded time spent in that behavior from the analysis. When possible, we observed individuals for 20 min or until they took 100 steps.

We estimated the location of focal individuals in the herd at the beginning, middle, and end of the observation period by recording the number of elk within the herd in a straight line from their head, rump, right flank, and left flank (Supporting Information S1: Figure A1:A), and the total number of adult elk within approximately five body‐lengths. We then calculated the average number of elk within five body‐lengths, the average number of sides exposed to the edge of the herd, and the average number of neighbors per side throughout the observation to classify the individual's herd position as central or peripheral without bias (Burger et al. 2000).

For each herd we also collected an ISS, in which we repeatedly scanned the entire herd and recorded the instantaneous behavior of each individual (Altmann 1974; St‐Louis and Côté 2012). To measure the effect of the location of an individual in the herd, we scanned all the peripheral animals, which were individuals on the outer edge of the herd and which lie on a line circumscribing the herd (Supporting Information S1: Figure A1:B). We then immediately scanned all the interior animals which were contained by that line (Dalmau et al. 2010). We waited 2 min between scans, until we collected five peripheral and five central scans. To increase independence among ISSs, in which the combined 10 scans form one sample, we collected one ISS per herd per day.

For each observation, we recorded the date, time, herd size, sex and age composition, and estimated the location of the elk on topographic maps. We also calculated the Euclidean distance between the observed elk herd and the observer. From the herd composition, we calculated the ratio of calves (< 1 year) to adult females (> 2 years) and the ratio of adult males to adult females. We removed any focal observations that were less than 2 min or had fewer than 10 steps taken by the individual, and any scan observations with fewer than three samples. If human disturbance (e.g., vehicle or pedestrian approaching the observed herd) occurred during the survey, we recorded the time of the event and ended the survey.

### 3.2 Temporal Covariates

We collected observations across all diel periods, defined as dawn (1 h before or after sunrise), day, dusk (1 h before or after sunset), and night (Palmer et al. 2021). We collected observations at night using a 4th‐generation thermal monocular with 7‐28× magnification (ATN Corp, Doral, FL) to provide insight into the dynamics between elk foraging behavior and risk from partly nocturnal predators (Roberts et al. 2017; Kohl et al. 2018). We categorized seasons as winter (January–April; the period with the majority of snowfall), spring (May–June; calving period), monsoon (July–August; during the majority of summer rainfall), and fall (September–December; the majority of elk hunting seasons).

### 3.3 Habitat Covariates

For all observations, we extracted values for dominant vegetation type, vegetation height class, vegetation cover, burn history and severity, human density, distances to roads, recreation sites and trails, forest ecotone, water, and private land, percent canopy cover and canopy openness, elevation, slope, northness, vector ruggedness measure (VRM), and density of roads using package *terra* in program R version 4.2.2 (Hijmans 2023, R Core Team 2022; see Supporting Information S1: Table A2 for data sources and manipulations). We classified roads as type 1 (high‐use gravel or paved roads accessible to low‐clearance vehicles), type 2 (moderate‐use dirt or gravel roads that may require moderate clearance vehicles), and type 3 (low‐use, unmaintained dirt roads requiring high‐clearance vehicles; BLM 2021; USFS 2021; USGS 2021). Additionally, we buffered the extracted points to a 200 m circular radius to account for herd movement during an observation and calculated the mean value of continuous variables and the mode of categorical variables within the buffer.

The normalized difference vegetation index (NDVI) is correlated with primary productivity (Schloss et al. 1999) and has been used as a proxy for forage quality and availability for ruminants (Boone et al. 2006). NDVI is also positively correlated with ungulate body condition where crude protein is a limiting resource (Ryan et al. 2012), though its accuracy can be affected by habitat, spatial resolution of the imagery, and diet of the target species (Creech et al. 2016). We extracted NDVI from pre‐processed MODIS satellite imagery with a spatial resolution of 250 × 250 m and a temporal resolution of eight days using the R package *MODIStsp* (Didan 2023). We also modeled the Instantaneous Rate of Green‐up using these NDVI values and the R package *irg* (Robitaille 2021). Across the study area, NDVI values typically peaked in mid‐August while troughs typically occurred between January and June (Supporting Information S1: Figure A2).

### 3.4 Predation Risk Covariates

Methods for quantifying predation risk can be categorized into three broad categories: risky places, risky times, and habitat characteristics (Moll et al. 2017). These metrics are not mutually exclusive and can represent risk at varying spatial and temporal scales. Comparing the effects of multiple risk metrics simultaneously allows researchers to quantify how each metric represents risk as it is potentially perceived by prey, as well as to test competing hypotheses (Lima and Bednekoff 1999; Moll et al. 2017; Brice et al. 2025). The perception of predation risk by prey, which may not represent “true risk”, or probability of death, is likely affected by a wide range of factors rather than by the mere presence of a predator (Hebblewhite, Merrill, and McDonald 2005). Therefore, we quantified predation risk using multiple indices for both wolves and mountain lions.

#### 3.4.1 Predator Encounter Risk

The Mexican wolf interagency field team (IFT) maintains collars on approximately 50% of the minimum population count with at least one collared individual in most packs. We used GPS collar data to estimate seasonal, yearly, and global (all years combined) Mexican wolf utilization distributions (UDs) for the wolf population and unique packs (Supporting Information S2: Figure B1; Thompson et al. 2025). We then multiplied pack UDs by pack size to account for the differences in risk associated with different pack sizes (Hebblewhite, Merrill, and McDonald 2005; Hebblewhite and Merrill 2007). We also multiplied these UDs by seasonal wolf habitat selection functions (HSFs) created by Thompson et al. (2025) to predict long‐term wolf presence based on habitat characteristics, and used this metric to represent the risk of encountering Mexican wolves (Supporting Information S2: Tables B1 and B2 and Figures B2 and B3). Prior to multiplication, we added 1 to the HSF values and 2 to the UD values to prevent one metric with a value of zero from canceling the other out, and to place greater weight on the UD values as they are a measure of true rather than predicted presence (Hebblewhite and Merrill 2007; Thompson et al. 2025). For mountain lion presence, we used the same methods to create mountain lion UDs (Supporting Information S3: Figure C2) and HSFs using data from GPS‐collared mountain lions in New Mexico deployed by the New Mexico Department of Game and Fish and multiplied these together following the same steps as for wolves, but did not incorporate group size because mountain lions are typically solitary hunters (Supporting Information S3: Tables C1 and C2; Figures C3 and C4). These steps resulted in three population risk metrics (seasonal, yearly, and global) for wolves and mountain lions, and three additional pack‐level risk metrics for wolves.

#### 3.4.2 Predator Kill Risk

We performed cluster investigations of collared Mexican gray wolves (2019–2023) and mountain lions (2021–2023) in addition to mortality investigations of collared adult female and calf elk (2019–2023; Supporting Information S4: Figure D1; Supporting Information S5: Figure E1). Because the presence of a predator does not necessarily lead to a predation event and prey may perceive the highest risk in places or times when they are most likely to be predated (Lima and Dill 1990; Hebblewhite, Merrill, and McDonald 2005; Suraci et al. 2022), we evaluated “risky places” models for wolves (Supporting Information S4: Tables D1, D2 and Figures D2, D3) and mountain lions (Supporting Information S5: Tables E1, E2 and Figures E2, E3) based on habitat characteristics of sites where elk were killed by either predator. Though wolves are cursorial predators that may pursue prey up to multiple kilometers to a final kill site (Paquet 1992; Wikenros et al. 2009), Kauffman et al. (2007) demonstrated that habitat characteristics of initial encounter sites between wolves and elk were similar to those of kill sites. Mountain lions may only chase prey approximately 50 m from the chase initiation site, but may drag carcasses up to 350 m from the kill site, and most cache sites are > 80 m from the kill site (Beier et al. 1995). Due to the potential disconnect between chase initiation sites, where elk should perceive risk, and kill sites, where carcasses are located by field personnel, we buffered kill site locations by 150 m for each predator when extracting habitat covariates. As with the presence metrics, we calculated both the wolf and mountain lion kill risk values at each observation location using habitat covariate values, and multiplied each risk metric by the wolf and mountain lion UD values with 1 added to the kill risk metric and 2 to the UD value.

#### 3.4.3 Temporal Predation Risk

For a fine‐scale temporal metric of wolf risk (risky times), we analyzed wolf collar data to estimate the distance to the nearest collared wolf at the time of each observation. Because of the variable and sometimes large fix rate (≤ 13 h) of the Mexican wolf GPS collars, we considered all wolf points within 24 h prior to an observation and identified the closest point. Research has shown that elk may detect wolves and alter their behavior when wolves are within 3 km (Liley and Creel 2008), so we also created a binary variable indicating whether a wolf was within 3 km or not in the preceding 24 h. While this is a coarse spatiotemporal resolution, we intended for this variable to account for wolf pack presence and movement in the portion of their home range that was in proximity to the observed elk herd, but undetected by collar data due to the wide fix intervals. Wolf vocalizations may be heard by humans at distances up to 2.5 km (Fuller and Sampson 1988) and by wolves up to 10 km (Harrington and Mech 1979). Because other studies found changes to prey behavior in response to predator presence over similar distances or time frames (Valeix et al. 2009; Basille et al. 2015), we hypothesized that elk may alter vigilance behavior in response to a binary variable of this resolution. We included an interaction between this binary variable and predicted wolf presence to test whether elk in this study area exhibited evidence for the risk allocation hypothesis (Lima and Bednekoff 1999; Creel et al. 2008). It was not feasible to create a similar metric for mountain lions because the population size, distribution, and proportion of mountain lions in the study area that were collared during this study were unknown.

Prey may also alter their behavior based on the diel activity of predators (Vander Vennen et al. 2016). Thompson et al. (2025) found seasonal variation in the activity levels of Mexican wolves; therefore we used cyclic generalized additive mixed‐effect models (GAMMs) to predict the seasonal activity rates of wolves and mountain lions across the diel cycle in 2‐h increments (Supporting Information S2: Figure B4; Supporting Information S3: Figure C1; Kohl et al. 2018). Using the resulting models, we predicted wolf and mountain lion activity at the times of elk observations.

### 3.5 Elk Behavior Modeling

#### 3.5.1 A Priori Model Construction

We constructed eight hypothesis‐driven a priori base model structures including combinations of temporal, social, human, and NDVI covariates (Supporting Information S6: Table F1) based on previous studies of ungulate foraging and vigilance behavior. Prior to fitting models, we assessed all covariates for multicollinearity by calculating Spearman's correlation coefficients for continuous covariates. Any covariates with a correlation coefficient > |0.6| were not included in the same model. We included variables that made the most ecological sense and resulted in improved model performance based on expected log pointwise predictive density (ELPD) score. We centered and scaled all continuous covariates to have a mean of zero and a standard deviation of one.

For each group of models, we used Pareto‐smoothed importance sampling leave‐one‐out cross‐validation (PSIS‐LOO CV) to compare individual models based on their predictive performance using the R package *loo* (Sivula et al. 2022). We identified competing models for each response variable as those with ELPD difference standard errors that overlapped zero, and then used pseudo‐Bayesian model averaging weights to determine which model or model subset comprised the majority of the weight (Table 1; See Supporting Informations S6–Tables F4–F8 for complete model selection tables; Filion et al. 2020; Lassiter et al. 2021). We calculated variance inflation factors (VIFs) for the most supported models and ensured that all VIFs for models without interaction terms were less than 2.5 (Johnston et al. 2018). After fitting models, if any covariates had no significant relationship (95% CrI overlapped zero) to the response variable across all a priori models, we removed them from all structures to increase parsimony. We identified the top‐ranked base model, and then tested whether including wolf or mountain lion predation risk metrics improved this model (Supporting Information S6: Table F2). This stepwise approach allowed us to test whether predation risk improved models without introducing an unreasonable number of competing structures.

**TABLE 1:** Competing model structures (models with standard error differences (ΔSE Estimated Log Pointwise‐Predictive Density [ELPD]) larger than ΔELPD and weights (*w*) ≥ 0.08) for all response types for elk behavior in east‐central Arizona and west‐central New Mexico in 2022–2023.

Model	ΔELPD	ΔSE	ELPD	SE	*w*
*Individual behavior*					
S + Diel + Herd + Calf + R3_D_ + Human + S × Herd + **Wolf encounter**_**1**_	0.00	0.00	5675.44	96.33	0.42
S + Diel + Herd + Calf + R3_D_ + Human + S × Herd + **Wolf kill**	−0.49	1.11	5674.95	96.32	0.25
S + Diel + Herd + Calf + R3_D_ + Human + S × Herd	−1.00	2.68	5674.45	96.16	0.15
S + Diel + Herd + Calf + R3_D_ + Human + S × Herd + **Lion encounter**_**1**_	−1.67	2.89	5673.77	96.20	0.08
*Individual proportion foraging*					
S + Diel + Herd + R3_D_ + S × Herd + **Lion encounter**_**1**_ + **Wolf kill** + **Wolf**_**D**_	0.00	0.00	184.23	18.93	0.20
S + Diel + Herd + R3_D_ + S × Herd + **Lion encounter**_**1**_	−0.32	1.89	183.91	18.56	0.14
S + Diel + Herd + R3_D_ + S × Herd	−0.43	2.40	183.80	18.52	0.13
S + Diel + Herd + R3_D_ + S × Herd + **Wolf encounter**_**1**_ + **Wolf**_**D**_	−0.45	1.20	183.78	18.84	0.13
S + Diel + Herd + R3_D_ + S × Herd + **Wolf**_**D**_	−0.58	2.03	183.64	18.63	0.11
S + Diel + Herd + R3_D_ + S × Herd + **Wolf kill**	−0.59	2.06	183.64	18.66	0.11
S + Diel + Herd + R3_D_ + S × Herd + **Wolf encounter**_**1**_	−0.73	2.07	183.50	18.64	0.10
*SPM*					
S + Diel + Herd + Calf + S × Herd + **Lion kill**	0.00	0.00	−2039.04	45.38	0.32
S + Diel + Herd + Calf + S × Herd	−0.31	2.64	−2039.35	46.77	0.23
S + Diel + Herd + Calf + S × Herd + **Lion encounter**_**2**_	−0.35	1.64	−2039.39	45.97	0.22
*Multitasking*					
S + Diel + Herd + Calf + Human + S × Calf + **Wolf**_**UDy**_ + **Wolf**_**Dcat**_ + **Lion encounter**_**3**_ + **Wolf**_**UDy**_ × **Wolf**_**Dcat**_	0.00	0.00	−1918.50	20.01	0.48
S + Diel + Herd + Calf + Human + S × Calf + **Wolf**_**UDy**_ + **Lion encounter**_**3**_	−0.56	2.02	−1919.06	19.82	0.28
S + Diel + Herd + Calf + Human + S × Calf + **Lion encounter**_**3**_	−1.76	2.48	−1920.26	19.67	0.08
*Herd behavior*					
S + Diel + Herd + Calf + R3_D_ + **Wolf encounter**_**2**_ + **Wolf**_**D**_	0.00	0.00	4300.15	80.24	0.62
S + Diel + Herd + Calf + R3_D_ + **Wolf**_**D**_	−0.66	2.48	4299.49	80.11	0.32

*Note:* Predation risk metrics are shown in bold. Universally uninformative parameters were removed from all models except when included in an interaction. See Supporting Information S6: Tables F4–F8 for complete model selection tables. Model terms: S = season; Diel = diel period; Herd = herd size; Calf = calf: cow ratio; R3_D_ = distance to unmaintained roads; Human = human density; Wolf enounter_1_ = Mexican wolf HSF weighted by yearly pack UD and average yearly pack size; Wolf kill = Mexican wolf risky places model weighted by yearly pack UD and average yearly pack size; Lion encounter_1_ = unweighted mountain lion HSF; Wolf_D_ = distance to nearest collared Mexican wolf in the past 24 h; Lion kill = mountain lion risky places model weighted by seasonal population UD; Lion encounter_2_ = mountain lion HSF weighted by seasonal population UD; Wolf_UDy_ = yearly Mexican wolf pack UD; Wolf_Dcat_ = categorical Mexican wolf distance (1 = collared wolf was within 3 km in past 24 h, 0 = no collared wolf was within 3 km in past 24 h); Lion encounter_3_ = mountain lion HSF weighted by global population UD; Wolf encounter_2_ = Mexican wolf HSF weighted by global pack UD and average pack sizes.

#### 3.5.2 Behavioral Models for Focal and Scan Samples

Both focal and herd scan samples resulted in compositional data representing the proportions of time spent in each behavior and the proportions of occurrences of each behavior, respectively. The Dirichlet distribution is appropriate for modeling the effects of covariates on compositional data summing to one with > 2 response components (Douma and Weedon 2019). For focal observations, we modeled the proportion of time spent foraging, being vigilant, multitasking, traveling, and other. Similarly, for each of the herd scans we modeled the proportion of animals engaged in each behavior out of the total number of animals in each scan. We used the alternative parameterization within the Dirichlet regression which sets one of the response variables as a reference component (“C”; Douma and Weedon 2019). This reference component is then modeled implicitly through the linear predictors for the remaining *C—1* components. We set “traveling” as the reference for the individual behavior models. Because these data contained frequent zeroes and ones, we transformed the data using the formula recommended by Douma and Weedon (2019). The Dirichlet distribution allows the prediction of the response of each behavior category to a range of values for each covariate, while holding the values of all other covariates at their scaled mean. Only a small proportion (21%) of our observations included focal samples from > 1 animal in the herd, so we did not include a random effect for unique herds.

#### 3.5.3 Individual Proportion Foraging

We also isolated the proportion of time spent foraging out of the total focal observation duration, which represents the foraging efficiency or “rate of capture” of forage (Owen‐Smith 1979). We modeled this response variable to investigate variables that affected foraging time alone. While no zeroes occurred in this dataset, true ones did exist so we transformed the data between zero and one. We modeled this response variable using a beta distribution with a logit link.

#### 3.5.4 Foraging Step Rate (SPM)

Because not all focal observations reached 100 steps, we calculated the number of foraging steps a focal animal took per minute by dividing the total number of steps taken while foraging, multitasking, or being vigilant, excluding steps taken while traveling (with head up) or engaging in other behaviors, by the time the individual spent foraging, being vigilant, or multitasking. SPM then represents the movement rate of an individual while actively foraging, and therefore the perceived abundance of forage present since elk would need to move more (take more steps) under poor perceived forage conditions (Owen‐Smith 1979, Smith and Cain III 2009). Including time spent being vigilant or multitasking in this calculation allowed us to draw conclusions based on how these interrupting behaviors affected the SPM. We then modeled step rate using a gamma distribution bounded between 0 and infinity with a log link.

#### 3.5.5 Modeling Rates of Multitasking

To model the frequency at which elk engage in multitasking versus intense vigilance, we constructed a binary response variable with each instance of vigilance behavior from focal samples coded as multitasking (1) or intensely vigilant (0). We then modeled this as a Bernoulli response variable and included a random intercept for unique individuals in the multitasking models due to multiple observations from a single individual (Zuur et al. 2009).

#### 3.5.6 Model Fitting for All Response Variables

We fit the five generalized linear and mixed model types (individual overall behavior, proportion foraging, SPM, multitasking, and herd behavior) in a Bayesian framework using the *brms* package (Burkner 2017) in program R. We ran all models with uninformative priors, using a normal distribution centered on zero for beta estimates, a student *t* distribution centered on zero for intercepts, and a gamma distribution with a shape and rate of 0.1 for precision. We ran models with five Markov Chain Monte Carlo (MCMC) chains, with 10,000 iterations per chain and a burn‐in period of 6000 iterations. We assessed models for convergence by visually inspecting MCMC plots, ensuring R‐hat values were less than 1.1, and confirmed tail effective sample sizes were > 1000 samples (Gelman and Shirley 2011; Supporting Information S6: Table F3).

## 4 Results

### 4.1 Behavioral Observations

Between January 2022 and June 2023, we collected 595 focal and 352 scan observations from 468 herds (Figure 1). We collected observations at dawn (*n* = 114 [focal]; *n* = 84 [scan]), dusk (*n* = 199 [focal]; *n* = 111 [scan]), day (*n* = 209 [focal]; *n* = 116 [scan]), and night (*n* = 73 [focal]; *n* = 41 [scan]), and during winter (*n* = 216 [focal]; *n* = 183 [scan]), calving season (*n* = 212 [focal]; *n* = 101 [scan]), monsoon season (*n* = 145 [focal]; *n* = 59 [scan]), and hunting season (*n* = 22 [focal]; *n* = 9). We observed focal individuals for a mean of 9.53 min ±6.75 (SD) and individuals took an average of 90.40 steps ±20.74 (SD). We collected a minimum of 3 scan samples for each scan observation. The mean herd size across observations was 27 ± 39 (SD) individuals. For focal observations, elk were on average foraging 72.4% ± 23.6% (SD) of the time, intensely vigilant 8.0% ± 11.8% (SD), multitasking 5.0% ± 8.1% (SD), traveling 12.1% ± 15.3% (SD), and engaging in other behaviors 2.6% ± 9.2% (SD). Their mean number of steps/min taken while foraging was 12.5 ± 21.9 (SD) steps/min. For scan samples, the mean percent of elk in the scan that were foraging was 63.9% ± 26.0% (SD), intensely vigilant 10.1% ± 13.0% (SD), multitasking 2.4% ± 4.5%, traveling 11.4% ± 14.0% (SD), and other 1.4% ± 5.8% (SD). We recorded 3054 instances of multitasking or vigilance in focal individuals, which we included in multitasking probability models.

### 4.2 Model Results

Across all model types, we found that the position of elk in the herd, individual sex, herd ratio of males to females, vegetation type, height, and cover, burn history and severity, canopy cover and openness, distances to recreation sites and trails, forest ecotone, private land, and water, elevation, slope, VRM, predator diel activity, and interactions between predation risk and diel period or season were either universally uninformative or performed worse than correlated covariates and we excluded them from final model sets.

### 4.3 Individual Behavior

**FIGURE 2:**
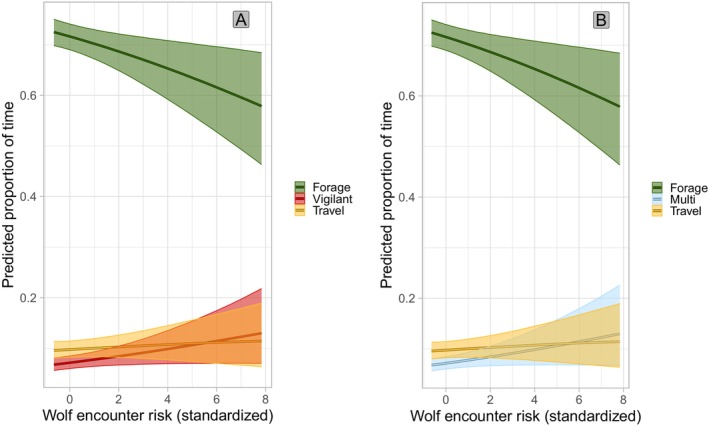
Effect of Mexican wolf encounter risk (Mexican wolf HSF weighted by yearly pack UD and average yearly pack size) in east‐central Arizona and west‐central New Mexico between 2022 and 2023 on the predicted proportion of time an individual elk spent foraging, intensely vigilant (A), multitasking (B), or traveling during a foraging bout with 95% credible intervals. Intense vigilance and multitasking overlapped directly and needed to be plotted separately. “Other” behaviors were modeled but excluded from plots for clarity.

**FIGURE 3:**
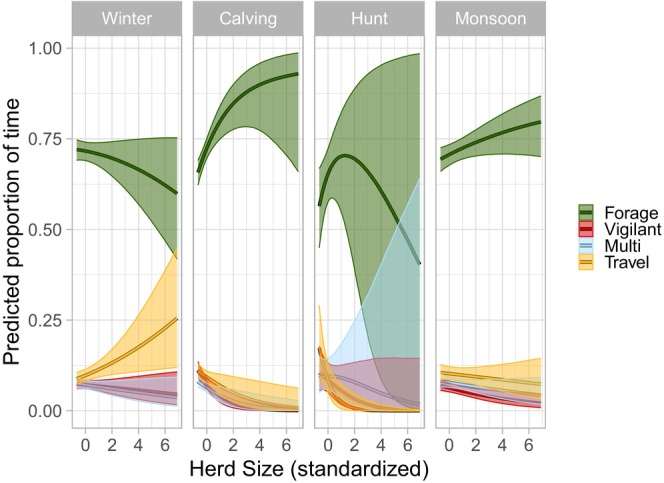
Effect of herd size on predicted proportions of time individual elk spent foraging, intensely vigilant, multitasking, and traveling during foraging bouts, conditional on season in east‐central Arizona and west‐central New Mexico between 2022 and 2023 with 95% credible intervals. “Other” behaviors were modeled but excluded from plots for clarity. Winter was defined as January to April, calving season as May to June, monsoon season as July to August, and hunting season as September to December.

The top‐ranked individual behavior model (*w* = 0.415, Table 1) included season, diel period, herd size, calf: cow ratio, distance to the nearest unmaintained road, human density, an interaction between season and herd size, and predicted wolf encounter risk (Table 1). The second‐ranked model (*w* = 0.254, Table 1) included wolf kill risk rather than wolf encounter risk. Based on the top‐ranked model, in areas where elk were more likely to encounter Mexican wolves, individuals were predicted to spend less time foraging and more time intensely vigilant and multitasking (Figure 2). In the competing model elk exhibited the same behavioral patterns in response to kill risk as to encounter risk. In winter, individuals were predicted to spend more time traveling during foraging bouts (i.e., a period of active foraging uninterrupted by resting) when in larger herds but exhibited no difference in time spent foraging, multitasking, or being intensely vigilant (Figure 3). Comparatively, in calving and monsoon season individuals were predicted to spend more time foraging and less time intensely vigilant and multitasking when in larger herds. Individuals were also predicted to spend less time traveling in larger herds in calving and hunting seasons. The predicted proportion of time an individual spent foraging was lower at dawn compared to day and predicted proportions of intense vigilance and traveling were higher at dawn compared to day (Supporting Information S6: Figure F4:A). Individual behavior at dusk and night did not differ from day. Individuals were predicted to spend less time foraging and more time traveling and intensely vigilant when further from unmaintained roads, and less time intensely vigilant in areas with higher human population densities (Supporting Information S6: Figure F4:B,C). When a higher ratio of calves to adult females was present in the herd, individuals were predicted to spend more time intensely vigilant and less time multitasking (Supporting Information S6: Figure F4:D).

**FIGURE 4:**
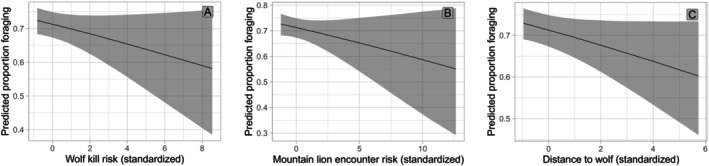
Effects of (A) Mexican wolf kill risk (Mexican wolf risky places model weighted by yearly pack UD and average yearly pack size), (B) mountain lion encounter risk (unweighted mountain lion HSF), and (C) the distance to the nearest collared Mexican wolf in the 24 h prior to an observation on the predicted proportion of time individual elk spent foraging during a foraging bout in east‐central Arizona and west‐central New Mexico. 95% credible intervals are shown in gray.

The top models predicting proportion foraging were similar to the individual behavior models, but with high uncertainty as model weight was similar across the top six models (Table 1). The top ranked model (*w* = 0.20, Table 1; Supporting Information S6: Figure F1) included season, diel period, herd size, distance to unmaintained roads, an interaction between season and herd size, predicted wolf kill risk, predicted mountain lion encounter risk, and the distance to the nearest collared Mexican wolf in the 24 h prior to the observation (Table 1). Human density and the ratio of calves to cows in the herd were uninformative in all foraging models in the initial model set, so we removed these variables from all competing models except when present in an interaction term and re‐ran these models. The competing models differed from the top‐ranked model by the inclusion of a subset of its predator covariates or the absence of any predator covariate (Table 1). The predictions for individual foraging were similar for season, diel period, herd size, distance to unmaintained road, the season and herd size interaction term, and wolf kill risk as to the multi‐category behavior models. Individuals were predicted to have a lower proportion foraging in areas with higher wolf kill risk or mountain lion encounter risk, and a higher proportion foraging when a collared Mexican wolf was in closer proximity in the preceding 24 h (Table 2; Figure 4A–C). The predicted proportion foraging was lower at dawn compared to day (Table 2; Supporting Information S6: Figure F5:A) and was greater in areas closer to unmaintained roads and in larger herds during calving and monsoon season compared to winter (Table 2; Supporting Information S6: Figures F5:B and F6). Foraging proportions did not differ between dusk or night and day.

**TABLE 2:** Beta estimates and 95% credible intervals (CrI) for top models predicting proportion foraging, foraging step rate (SPM), and multitasking probabilities for individual elk in east‐central Arizona and west‐central New Mexico in 2022–2023.

Parameter	β	SD	95% CI	Propn
*Individual proportion foraging*				
Calving season	0.17	0.11	−0.06, 0.39	0.93
Monsoon season	−0.04	0.11	−0.26, 0.18	0.65
Hunting season	−0.52	0.27	−1.03, 0.02	0.97
Dawn	**−0.25**	**0.12**	**−0.48, −0.01**	**0.98**
Dusk	0.10	0.10	−0.10, 0.30	0.82
Night	0.23	0.15	−0.07, 0.54	0.94
Herd size	−0.12	0.07	−0.25, 0.02	0.96
Dist. to unmaintained road	**−0.21**	**0.04**	**−0.29, −0.13**	**1.00**
Mountain lion encounter risk	−0.06	0.04	−0.14, 0.03	0.90
Mexican wolf kill risk	−0.07	0.05	−0.16, 0.02	0.93
Mexican wolf distance	−0.09	0.05	−0.17, 0.01	0.97
Herd size × Calving season	**0.60**	**0.19**	**0.24, 0.97**	**1.00**
Herd size × Monsoon season	**0.24**	**0.09**	**0.07, 0.43**	**1.00**
Herd size × Hunting season	0.62	0.51	−0.27, 1.74	0.91
*SPM*				
Calving season	0.02	0.10	−0.17, 0.21	0.57
Monsoon season	**0.35**	**0.10**	**0.16, 0.54**	**1.00**
Hunting season	0.20	0.23	−0.23, 0.66	0.81
Dawn	**0.28**	**0.11**	**0.07, 0.49**	**1.00**
Dusk	−0.06	0.09	−0.23, 0.11	0.76
Night	**−0.34**	**0.14**	**−0.61, −0.06**	**0.99**
Herd size	**0.26**	**0.07**	**0.13, 0.40**	**1.00**
Calf: cow ratio	−0.06	0.04	−0.13, 0.01	0.96
Mountain lion kill risk	−0.06	0.04	−0.14, 0.01	0.95
Herd size × Calving season	**−0.56**	**0.16**	**−0.87, −0.24**	**1.00**
Herd size × Monsoon season	**−0.36**	**0.08**	**−0.52, −0.21**	**1.00**
Herd size × Hunting season	−0.43	0.30	−0.97, 0.20	0.92
*Multitasking*				
Calving season	**−0.52**	**0.17**	**−0.86, −0.20**	**1.00**
Monsoon season	**0.54**	**0.18**	**0.18, 0.90**	**1.00**
Hunting season	0.81	0.41	0.00, 1.60	0.98
Dawn	**−0.42**	**0.19**	**−0.80, −0.04**	**0.99**
Dusk	−0.26	0.16	−0.58, 0.06	0.95
Night	**−0.86**	**0.24**	**−1.35, −0.40**	**1.00**
Herd size	0.06	0.06	−0.06, 0.19	0.84
Calf: cow ratio	**−0.18**	**0.08**	**−0.34, −0.04**	**0.99**
Human density	0.12	0.07	−0.02, 0.26	0.95
Wolf UD	−0.09	0.07	−0.24, 0.06	0.89
Mexican wolf w/in 3 km	0.35	0.22	−0.08, 0.78	0.94
Mountain lion encounter risk	**−0.20**	**0.07**	**−0.34, −0.06**	**1.00**
Calf ratio × Calving season	**−0.63**	**0.28**	**−1.18, −0.08**	**0.99**
Calf ratio × Monsoon season	0.21	0.27	−0.33, 0.74	0.78
Calf ratio × Hunting season	0.16	0.41	−0.64, 0.98	0.65
Wolf UD × Wolf w/in 3 km	**−0.63**	**0.23**	**−1.09, −0.18**	**1.00**

*Note:* Propn represents the proportion of posterior draws which have the same sign as the mean (β). Model terms with 95% CrIs not overlapping zero are shown in bold. Model reference categories: Season = winter; diel period = day; categorical Mexican wolf distance = no collared wolf was within 3 km in the prior 24 h.

**FIGURE 5:**
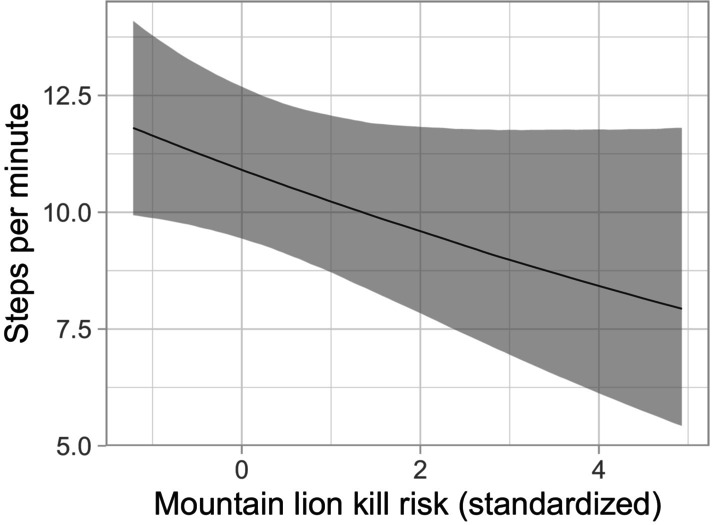
Effect of mountain lion kill risk (mountain lion risky places model weighted by seasonal population UD) on the predicted number of steps individual elk took per minute while foraging in east‐central Arizona and west‐central New Mexico. 95% credible intervals are shown in gray.

The top‐ranked model (*w* = 0.32) for SPM included season, diel period, herd size, the calf to cow ratio in the herd, an interaction between season and diel period, and mountain lion kill risk (Table 1; Supporting Information S6: Figure F2). There were two competing models: one which did not include a predation risk variable (*w* = 0.23, Table 1) and one which included mountain lion encounter risk rather than kill risk (*w* = 0.22, Table 1). In the top model, individuals were predicted to take fewer steps/min (SPM) in areas where they were more likely to be killed by mountain lions (Table 2; Figure 5). Individuals were predicted to take more SPM at dawn and fewer SPM at night compared to day (Table 2; Supporting Information S6: Figure F7:A). The predicted step rate during dusk did not differ from day. Elk were also predicted to take fewer SPM in herds with a higher ratio of calves to cows (Table 2; Supporting Information S6: Figure F7:B), and in larger herds elk were predicted to take fewer SPM during calving and monsoon season, but more SPM during the winter (Table 2; Supporting Information S6: Figure F8).

**FIGURE 6:**
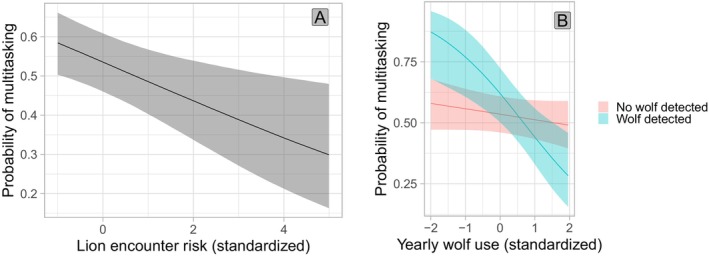
Effects of (A) mountain lion encounter risk (mountain lion HSF weighted by global population UD) and (B) yearly Mexican wolf population UD on the probability of individual elk multitasking in east‐central Arizona and west‐central New Mexico between 2022 and 2023, conditional on whether a collared Mexican wolf was detected within 3 km in the 24 h prior to an observation with 95% credible intervals.

The probability of an individual elk multitasking versus being intensely vigilant in a given instance of vigilance was predicted by relatively complex models. The top‐ranked model comprised 48.3% of the total weight and included season, diel period, herd size, the calf to cow ratio in the herd, human density, an interaction between season and the calf to cow ratio, a yearly Mexican wolf pack UD, a binary variable for whether a collared Mexican wolf was located within 3 km in the 24 h prior to the observation, an interaction between the wolf UD and wolf distance variable, and mountain lion encounter risk (Table 1; Supporting Information S6: Figure F3). Distance to unmaintained road was uninformative and was removed. One additional model comprised a similar proportion of weight (*w* = 0.28) and included the same terms except for Mexican wolf distance and the interaction term between wolf distance and the wolf UD. Based on the top‐ranked model, individual elk were less likely to multitask in areas where they were more likely to encounter mountain lions (Table 2; Figure 6A). When a collared wolf was detected within 3 km in the prior 24 h, yearly wolf use had a strong negative association with multitasking probability and therefore a strong positive association with intense vigilance (Figure 6B). Individuals were less likely to multitask at dawn and night compared to day, but dusk did not differ significantly from day (Table 2; Supporting Information S6: Figure F9:A). The probability of multitasking was higher in areas with greater human population densities and marginally higher in larger herds (Table 2; Supporting Information S6: Figure F9:B,C). Multitasking probability particularly decreased as calf to cow ratio increased during calving season, but also decreased in winter (Supporting Information S6: Figure F10). The calf to cow ratio had no effect during monsoon and hunting season.

### 4.4 Herd Behavior

**FIGURE 7:**
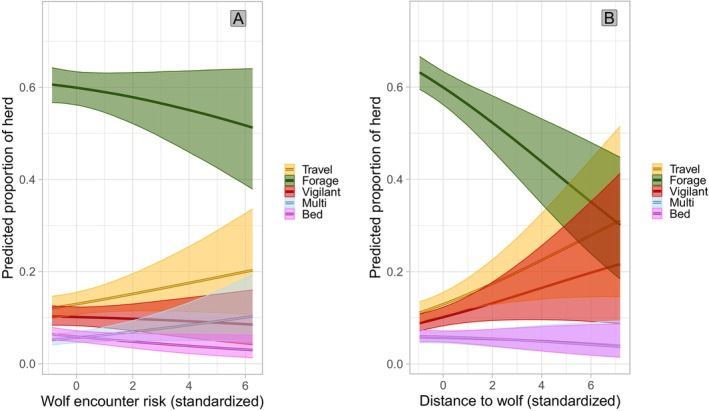
Effect of (A) Mexican wolf encounter risk (Mexican wolf HSF weighted by global pack UD and average pack sizes) and (B) distance to the nearest collared Mexican wolf in the 24 h prior to an observation on the predicted proportion of an elk herd engaged in foraging, intense vigilance, multitasking, traveling, and bedded behaviors in east‐central Arizona and west‐central New Mexico between 2022 and 2023 with 95% credible intervals. “Other” behaviors were modeled but excluded from plots for clarity.

The top‐ranked model (*w* = 0.62) predicting the average proportions of individual elk engaged in foraging, vigilance, multitasking, traveling, bedded behaviors, and other behaviors in a herd included season, diel period, herd size, calf to cow ratio, distance to the nearest unmaintained road, wolf encounter risk, and the distance to the nearest collared Mexican wolf location in the prior 24 h (Table 1). One competing model (*w* = 0.32) was identical but did not include wolf encounter risk. Human density was universally uninformative and we removed this term except when included in an interaction term. Higher probabilities of encountering a wolf did not affect the proportion of individuals foraging but were associated with higher proportions of multitasking and lower proportions of bedded individuals, and wolf encounter risk was not significantly associated with the proportion of vigilant individuals (Figure 7A). Surprisingly, when a collared Mexican wolf was in closer proximity in the past 24 h, a larger proportion of individuals were predicted to be foraging while a smaller proportion were predicted to be traveling and vigilant (Figure 7B). Fewer individuals in the herd were predicted to be foraging during hunting season compared to winter, while a greater proportion were predicted to be vigilant in hunting season compared to winter (Supporting Information S6: Figure F11:A). At dawn, the predicted proportion of the herd foraging was lower while the predicted proportion traveling was higher compared to day, and a larger proportion were bedded at night compared to day (Supporting Information S6: Figure F11:B). The predicted proportion of elk bedded increased with herd size and the predicted proportion of vigilant elk increased with the ratio of calves to cows in the herd (Supporting Information S6: Figure F11:C,D). At greater distances from unmaintained roads, a smaller proportion of individuals were predicted to be foraging while a larger proportion were predicted to be vigilant and bedded (Supporting Information S6: Figure F11: E).

## 5 Discussion

Anti‐predator and foraging behavior of actively foraging elk in this study was associated with predation risk. Individual elk in areas with a high risk of encountering Mexican wolves allocated a greater proportion of foraging bouts to intense vigilance and multitasking while reducing foraging time. These results support findings by Fortin, Boyce, Merrill, and Fryxell (2004) and Blanchard and Fritz (2007) which suggest ungulates foraging under high risk are unable to completely eliminate the tradeoff between foraging and intense vigilance by multitasking. Predation risk from Mexican wolves may increase the need for intense vigilance to detect nearby predators and require elk to trade off foraging time during foraging bouts. Elk may then compensate for reduced foraging time throughout the diel cycle by allocating a portion of their resting activity budget to foraging (Hamel and Côté 2008; Farley et al. 2024). This reduction in foraging time may explain the apparent higher probability of foraging reported by Farley et al. (2024) under higher risk from Mexican wolves during midday, which did not focus solely on actively foraging elk. Furthermore, we found that smaller proportions of herds were bedded in areas of higher Mexican wolf encounter risk which supports findings reported by Farley et al. (2024). While we did not observe a corresponding reduction in the proportion of foraging individuals in herds under increased wolf risk, Brereton et al. (2022) demonstrated that the continuous sampling method we employed for individual behavior produces less statistical bias than the pinpoint sampling method we used for herds. It is likely that instantaneously sampling the behavior of elk in the herd does not provide a complete or accurate account of their activity during an observation, and we therefore place more weight on the results of the individual behavior models. However, while the predator species and type of risk metric varied, the top supported models for each foraging and behavioral response measure included at least one predation risk metric. This variation may be due to the differing effects that coursing and stalking predators (i.e., Mexican wolves and mountain lions) have on the different aspects of elk foraging behavior measured in this study, as mountain lion, but not wolf, risk affected foraging step rates while wolf risk rather than mountain lion risk affected individual and herd behavior. Additionally, we found that higher‐performing risk metrics included a predicted use model multiplied by a UD and, for wolves, by pack size. This is consistent with previous work positing that weighting predictive risk covariates with both spatial distribution and density of predators is more representative of true risk (Hebblewhite and Merrill 2007). However, uncertainty existed between top models and therefore between specific risk variables. Only the top model for herd behavior comprised more than 50% of the cumulative model weight, and the top proportion foraging model only comprised 20% of the total model weight. However, all competing models for multitasking or herd behavior included predation risk metrics and competing models for proportion foraging, SPM, and individual behavior which did not include a risk metric comprised less than a quarter of cumulative model weight. This suggests that, while uncertainty remains among predation risk prediction variables, predation risk is likely related to elk foraging behavior in our system.

Our findings further demonstrate the importance of distinguishing between types of vigilance and evaluating multiple metrics and scales of predation risk (Moll et al. 2017; Prugh et al. 2019), and imply that elk in the MWEPA use multitasking to reduce the costs of vigilance. The effect of the annual Mexican wolf UD on the binary probability of individual elk multitasking was conditional on whether a collared wolf was located within 3 km in the 24 h prior to an observation. Our study provides further support for the use of predator presence within the past 24 h as a metric for short‐term risk (Christianson and Creel 2008; Valeix et al. 2009; Périquet et al. 2012), though the reliability of our metric is limited by the presence of uncollared wolves and other predators (e.g., mountain lions, coyotes, and bears) on the landscape and a limited fix rate from the Mexican wolf collars. While the multitasking model does not account for a tradeoff between vigilance and foraging time, the highest levels of intense vigilance were predicted to occur with the greatest long‐term and short‐term risk from Mexican wolves. This does not support predictions under the predation risk allocation hypothesis and instead supports the risky places/times hypothesis (Lima and Dill 1990). Although no other studies we are aware of investigate the tradeoff we saw between multitasking and intense vigilance in response to an interaction of short‐term and long‐term predation risk, multiple studies have found that ungulates foraging under high long‐term risk increase vigilance only when a predator is in close proximity, likely to limit the costs of reduced foraging (Dröge et al. 2017; Eccard et al. 2017). These studies did not, however, differentiate multitasking and intense vigilance. Other studies of the effects of predation risk on foraging ungulates did differentiate these types of vigilance but considered either long‐term risk (Yiu et al. 2021) or short‐term risk alone (Périquet et al. 2012). Elk exposed to lower wolf risk may also be foraging under a sufficiently low risk threshold that they are not required to engage in intense vigilance at risky times. Elk in the MWEPA have also been shown to reduce risk through spatiotemporal separation (Thompson et al. 2025), which may affect the tradeoff between foraging and vigilance at risky times.

Our models predicted that elk reduce multitasking and foraging but may increase intense vigilance under increased risk from mountain lions, and may reduce step rates where they are more likely to be killed by mountain lions (though 95% credible intervals for the effect of mountain lion risk on proportion foraging and SPM overlapped zero by 10% and 5% respectively). While we initially hypothesized that step rates would be higher in high‐risk areas because elk have been shown to increase movement under elevated predation risk (Proffitt et al. 2009), our results did not support this. With vigilance time included in step rate calculations, time spent vigilant likely reduced the overall step rate under higher risk. Because a reduced step rate may be associated with higher forage quality (Owen‐Smith 1979), the relationship between mountain lion kill risk and step rate may be related to habitat characteristics (smaller distances to roads, trails, forest edges, and water bodies) and associated forage conditions included in the kill risk model. This supports findings by Paterson et al. (2022) which identified an association between high quality forage and mountain lion risk, but not wolf risk.

Elk may also reduce their step rate in response to the stalking strategy of mountain lions and the reduced detectability of predators in obstructed visual cover, as increased movement may increase their exposure to predators (Bassing et al. 2024). Our sample size for observations in areas with the highest risk from mountain lions was also likely limited due to the difficulty of observing elk in dense, obstructive vegetation. Kohl et al. (2019) found that elk in the Greater Yellowstone ecosystem spatiotemporally avoided both wolves and mountain lions because these predators occupied distinct spatial–diel hunting domains. Similarly, research in Washington state reported that elk selected habitats to minimize the risk of mountain lion predation at night and wolf predation during the day (Ganz et al. 2024). While our results support the theory that elk likely respond to spatial risk from both wolves and mountain lions, a future study that incorporates a fine‐scale temporal metric of mountain lion presence could help explain how elk alter foraging behavior in response to temporal risk from both predators. Additionally, because data were only available from collared mountain lions in New Mexico and not Arizona, the risk metrics weighted by UDs for mountain lions were likely less accurate for elk observed in Arizona.

We also found evidence of seasonal and diel variation in elk foraging behavior, as well as strong evidence for interacting effects of season, herd size, and herd composition. The observed increase in foraging and reduction in intense vigilance and multitasking by individuals in larger herds during calving season is likely due to the relative safety found in large groups during this risky time. Young calves are more vulnerable to predation from wolves and mountain lions, as well as a suite of other predators that pose little risk during the rest of the year such as coyotes, bobcats, and black bears. Larger herds may provide safety via the dilution of risk or “many eyes” effect by increasing the likelihood of predator detection which allows lactating females to reduce vigilance time and increase forage intake. However, a study of female elk vigilance and foraging time during calving season in Yellowstone National Park reported that elk with calves increased foraging time but did not reduce vigilance in larger herds while female elk without calves did (Childress and Lung 2003). Because we did not observe marked individuals or identify individuals as mothers, it is possible that this disparity is due to undetected differences in vigilance levels between elk with and without calves in our study. Farley (2022) found an increase in vigilance of female elk with calves in response to wolf risk but no differences in individual behavior when in herds of different sizes. The MWEPA supports a diverse predator guild and large herds likely allow increased foraging time without compromising risk avoidance for vulnerable calves.

In winter, the effect of herd size on foraging time was negative, likely due to intraspecific competition as time spent traveling also increased, indicating that elk in larger herds are required to move more while foraging to obtain adequate resources when forage conditions are poor. Risk reduction associated with larger herds in calving season may become competition during a resource‐scarce period (Blanchard and Fritz 2008). The seasonal variation in the effects of herd size found in our study may suggest an explanation for the conflicting effects documented in the literature. Some studies reported a decrease in vigilance in larger herds (Creel et al. 2008), others a decrease in foraging (Focardi and Pecchioli 2005; Pecorella et al. 2019), and some found no effect of group size on vigilance or foraging time (Laundré et al. 2001), or that herd density influenced vigilance while herd size did not (Smith and Cain III 2009). However, these studies did not test for an interaction between season and herd size. The influence of herd size on ungulate foraging and vigilance appears to depend on forage conditions and reproductive state, and these confounding variables should be accounted for when investigating group size effects.

Seasonal differences in anti‐predator behavior of elk in the MWEPA may also explain the seemingly paradoxical effect of short‐term Mexican wolf proximity on herd behavior and FIR. In contrast to our hypotheses, in which we predicted elk would reduce foraging and increase vigilance when a wolf was closer in the preceding 24 h, individual elk spent more time foraging, a greater proportion of the herd was foraging, and a smaller proportion of the herd was traveling or vigilant as the distance to the nearest collared wolf decreased. Liley and Creel et al. (2008) found that elk in Yellowstone National Park increased vigilance when a wolf pack was within 3 km, which seemingly contradicts our findings. However, that study also found that elk reduced vigilance when wolves were present if a recent kill was also detected. Our results may differ due to the 13‐h location intervals of most Mexican wolf collars and our use of the prior 24‐h window for collared wolf distances, whereas Liley and Creel et al. (2008) determined whether wolves were present concurrently with observations. A post hoc analysis of our data revealed that the mean distance from observed elk to the nearest collared wolf was greatest during calving season compared to all other seasons (Olson 2024). These differences may indicate that the association between wolf distance and elk behavior in the MWEPA could vary seasonally. Thompson et al. (2025) found that female elk did not select against areas with high Mexican wolf kill risk within their home range in all diel periods of spring, suggesting that elk may tolerate higher landscape‐level risk within their home range because they are under lower temporal risk (i.e., at greater distances to wolves) during this season. It is possible that female elk relocated further from wolf risk during calving season, as female elk with calves have been reported to avoid predation risk at the home‐range scale during calving season when there is not an associated tradeoff for forage availability (Berg et al. 2021). While there is no strong evidence for these home range shifts by female elk during calving season in the MWEPA, the increased distances between observed elk and Mexican wolves during calving season documented by our study warrant further research into calving range selection by parturient females in relation to Mexican wolf risk.

While a diel interaction with predation risk was not included in any final models due to its poor performance in preliminary analysis, elk behavior did differ across diel periods. Few studies have investigated elk vigilance and foraging behavior at night using direct observations, despite the risk created by nocturnal predators (but see Roberts et al. 2017). Our study found that the probability of multitasking and predicted step rates were both lowest at night. As suggested by Blanchard and Fritz (2007), ungulates may stop multitasking to mitigate noise created by chewing, so a need for increased auditory detection of predators at night may explain the large reduction in multitasking. While few studies have investigated the nocturnal vision capabilities of elk, activity peaks documented by Thompson et al. (2025) indicate that elk in this study area are crepuscular, consistent with other elk populations that concentrate foraging activity around dawn and dusk (Green and Bear 1990; Roberts et al. 2017). Studies of color vision and eye structure in crepuscular‐adapted white‐tailed deer have shown that these ungulates may have a limited ability to discriminate long‐wavelength light at night (VerCauteren and Pipas 2003) and possess only a moderate ratio of lens thickness to eye size, metrics typically correlated with nocturnal vision ability (D'Angelo et al. 2008). A reduced ability to visually detect predators at night, when wolves and mountain lions increase activity in this study area, may therefore require elk to cease processing forage while vigilant to more effectively monitor their environment for threats via multiple senses. Detection may also be affected by ambient light at night, which is related to moon phases and light pollution as seen in Palmer et al. (2017) and Barrientos et al. (2023) and warrants further investigation.

We found some evidence of a human refuge effect on foraging elk overall, but a potential risk effect during hunting season. Elk were more likely to multitask in areas of higher human population densities in all seasons, which could indicate that they perceive less intense risk in these areas. We also primarily observed elk from roads, which may have biased our data toward elk that are habituated to human presence. However, during hunting season individuals spent less time foraging regardless of herd size, and herds reduced foraging while increasing vigilance. Prey may use human presence as a buffer against predators that avoid humans (Hebblewhite, White, et al. 2005; Shannon et al. 2014; Ganz et al. 2024), while the intense and predictable predation risk created by hunting seasons has been shown to elicit strong behavioral responses in prey (Proffitt et al. 2009). Such a response was seen in our study, where elk fled quickly when observers approached during hunting seasons, resulting in small sample sizes in fall. Most fall observations occurred at night when elk were less likely to flee from observers. This prevented us from assessing interactions between human activity and season, and we were unable to disentangle the influence of human hunting risk and associated behavioral responses. Future work targeting elk behavior during hunting season alone and incorporating more detailed metrics of hunter activity could clarify the mechanisms driving the differences in behavior we observed. The increase in foraging for herds and individuals closer to unmaintained roads may have less to do with human presence and be more associated with forage conditions near these roads, as they are characterized by low human use. Additionally, because we primarily observed elk from roads in our study, bias may have been introduced to the effect of roads on elk behavior.

Our study provides an improved understanding of the mechanisms that drive elk foraging behavior in the MWEPA, documenting a reduction in individual foraging under increased wolf risk with increased vigilance during a foraging bout. This may be compensated for by trading resting time for foraging in the daily activity budget (Farley et al. 2024), as well as foraging in higher‐quality, high‐risk areas at low‐risk times (Thompson et al. 2025). We also provide evidence for the impacts of mountain lion risk on elk foraging behavior in the recovery range of Mexican wolves. Unlike Mexican wolves, mountain lions were never extirpated from the region and their influence on the ecosystem cannot be discounted, as they account for a significant portion of elk mortality in the MWEPA (Smith et al. 2023; Martinez 2024). The interacting effects of long‐term spatial wolf risk and short‐term wolf proximity on anti‐predator behavior documented in this study also suggest the ability of elk in this system to respond to risk dynamically with changes to vigilance and foraging. This supports previous evidence that elk in the MWEPA alter their use of areas with increased Mexican wolf risk based on multiple scales of temporal risk, potentially mitigating avoidance of high‐quality, high‐risk habitat (Thompson et al. 2025).

As the Mexican wolf population continues to grow, it will continue to be crucial to understand how this subspecies affects elk populations directly and indirectly. Mexican wolves, mountain lions, and humans all appear to influence elk behavior in the southwestern United States, and these differences in behavior could affect nutritional condition, especially in areas with the highest combined risk from these predators. However, our study was limited to immediate behavioral effects and did not investigate relationships between these behavioral differences and nutrition. Future research into a potential link between these non‐consumptive effects on prey behavior and subsequent effects on individual fitness and, ultimately, population dynamics is necessary to gain a better understanding of the consequences of predation risk for elk in the MWEPA. Studies of the influence of predation risk on stress hormones, individual nutritional condition, and calf recruitment rates could provide greater insight into the physical and demographic effects of non‐lethal risk (Creel et al. 2007; Christianson and Creel 2010). Incorporating knowledge of the effects of wolves and competing predators on elk behavior, habitat use, and demographics, as well as an understanding of how risk from these predators is impacted by human risk would allow for more informed management decisions.

## Author Contributions

**Julia E. Olson:** conceptualization (equal), data curation (equal), formal analysis (lead), investigation (lead), methodology (equal), writing – original draft (lead), writing – review and editing (lead). **Cara J. Thompson:** conceptualization (equal), formal analysis (equal), writing – original draft (equal), writing – original draft (equal). **Zachary J. Farley:** conceptualization (equal), writing – original draft (equal). **Samuel I. Martinez:** investigation (equal), writing – original draft (equal). **Scott T. Boyle:** investigation (equal), writing – original draft (equal). **Nicole M. Tatman:** conceptualization (equal), funding acquisition (equal), resources (equal), writing – original draft (equal). **James C. DeVos:** conceptualization (equal), funding acquisition (equal), resources (equal), writing – original draft (equal). **Stewart D. Liley:** conceptualization (equal), funding acquisition (equal), resources (equal), writing – original draft (equal). **James W. Cain III:** conceptualization (equal), data curation (equal), formal analysis (equal), funding acquisition (lead), investigation (equal), methodology (lead), project administration (lead), resources (lead), supervision (lead), writing – original draft (equal), writing – review and editing (equal).

## Conflicts of Interest

The authors declare no conflicts of interest.

## Supporting information


**Data S1:** ece372520‐sup‐0001‐Supinfo01.docx.

## Data Availability

Data are not uploaded as Supporting Information but will be available here as soon as USGS approves the data release: https://doi.org/10.5061/dryad.0000000hx.
