# Supplementary material for: Dynamic Risk From Mexican Wolves and Mountain Lions Influences Elk Foraging Behavior
Source: Ecol Evol. 2025 Dec 17;15(12):e72520. doi: 10.1002/ece3.72520 (PMC12712231; doi:10.1002/ece3.72520)
Supplement: Supplementary file 1 — Data S1: ece372520‐sup‐0001‐Supinfo01.docx. [file ECE3-15-e72520-s001.docx]

Supplementary Material for:

Olson, J.E., C.J. Thompson, Z.J. Farley, S.I. Martinez, S.T. Boyle, N.M. Tatman, J.C. deVos, Jr S.G. Liley, and J.W. Cain III. Dynamic risk from Mexican wolves and mountain lions influences elk foraging behavior. Ecology and Evolution.

Any use of trade, firm, or product names is for descriptive purposes only and does not imply endorsement by the U.S. Government.

SUPPLEMENTARY MATERIAL 1

BEHAVIOR AND COVARIATE DESCRIPTIONS

*Tables*

Table A1: Definitions of behavior categories for observation surveys.

| **Behavior** | **Description** |
| --- | --- |
| Foraging | Head is down, actively collecting food while grazing (feeding on grass or low-growing vegetation) or browsing (feeding on shrubs, trees, or other taller woody vegetation) and not lifting head while walking between bites |
| Intense vigilance | Head is up, ears forward, eyes focused, and not chewing or otherwise actively handling food |
| Multitasking | Vigilant with head up, ears forward, and eyes focused but also chewing |
| Traveling | Walking or running with head up and not vigilant nor collecting food |
| Bedded: Resting | Laying down with mandible on ground, not chewing or vigilant |
| Other | Other behaviors not listed that occur infrequently, including grooming, socializing, fighting, or others. |

| Table A2: Covariates included elk behavioral models and predation risk models with data sources and manipulations.   \| Variable \| Data source \| Data manipulation \| \| --- \| --- \| --- \| \| Herd size \| Observations \| None \| \| Calf:cow ratio \| Observations \| None \| \| Bull:cow ratio \| Observations \| None \| \| Sex (ref. = female) \| Observations \| None \| \| Exposure to edge of herd \| Observations \| Averaged the number of sides of an individual that were exposed to the edge of the herd. \| \| Season (ref. = winter) \| NA \| Winter was defined as Jan-Apr, calving season as May-Jun, monsoon as Jul-Aug, and hunting season as Sep-Dec. \| \| Diel period (ref. = day) \| NA \| Night was defined as between one hour after sunset and one hour before sunrise, and crepuscular periods (dawn and dusk) as one hour before or after sunrise and sunset. \| \| Vegetation type (ref. = grassland) \| LANDFIRE \| Reclassified into grassland, aspen, oak/shrub, pinon-juniper, ponderosa, mixed conifer, and wet meadow or pasture. \| \| Vegetation height (ref. = < 1 m) \| LANDFIRE \| Reclassified as unobstructed (< 0.5 m) and obstructed (> 0.5 m). \| \| Vegetation cover (ref. = <30%) \| LANDFIRE \| Reclassified as open (<30% cover) and closed (>30% cover). \| \| Horizontal cover (ref. = no cover) \| LANDFIRE \| Multiplied vegetation height by vegetation cover. \| \| Burned (ref. = unburned) \| NIFC, MTBS, and BAER \| Binary variable indicating whether the area burned in the past 17 years or not. \| \| Maximum burn severity \| MTBS and BAER \| Reclassified as unburned to high severity and converted to continuous variable. \| \| Fire history (ref. = unburned in past 17 years) \| NIFC, MTBS, and BAER \| Combined burn severity and years since fire into a single metric with classes representing age and severity: 0 (unburned in past 17 years), 1 (low-severity within 5 years), 2 (mod-high severity within 5 years), 3 (low-severity within 6-17 years), and 4 (mod-high severity within 6-17 years). \| \| Density of roads (3 types) \| USFS, BLM, AZDOT \| Quantity of roads in a 1.5 km x 1.5 km area for three road types: 1 (paved and high-use), 2 (dirt roads with moderate use), and 3 (unmaintained high-clearance dirt roads), and for all road types combined. \| \| Distance to roads (3 types) \| USFS, BLM, AZDOT \| Distance rasters for the three road types and all roads. \| \| Human density \| NASA SEDAC \| None \| \| Distance to recreation sites or trails \| USFS, BLM \| Distance raster to a ski resort, Sipe White Mountain wildlife viewing area, popular boating and fishing sites, campgrounds, and OHV and hiking trails. \| \| Distance to forest ecotone \| LANDFIRE \| Distance raster for where treed and open cover types met, buffered by 50 m. \| \| Percent canopy cover \| NLCD \| None \| \| Canopy openness \| NLCD \| Reclassified canopy cover raster as open (< 30% cover) and closed (≥ 30% cover) and summed the number of open pixels within 3 x 3, 8 x 8, and 16 x 16 moving windows. \| \| Distance to private land \| BLM \| Distances to polygons of private land. \| \| Elevation \| LANDFIRE \| None \| \| Slope \| LANDFIRE \| None \| \| Northness \| LANDFIRE \| Calculated aspect values from DEM and transformed to index of northness using the equation cos(x*(π/180)). Flat areas were given mean value. \| \| Topographic Ruggedness Index \| LANDFIRE \| Calculated from DEM using R spatialEco package. \| \| Topographic Position Index \| LANDFIRE \| Calculated from DEM using R spatialEco package. \| \| Normalized Difference Vegetation Index (NDVI) \| MODIS \| Extracted NDVI values from the image closest to the date of each observation. For observations that fell directly between two NDVI raster dates, we averaged values from the two rasters. \| \| Instantaneous Rate of Green-up (IRG) \| MODIS \| Modeled Instantaneous Rate of Green-up from raw daily NDVI values. \| |
| --- | --- | --- | --- | --- | --- | --- | --- | --- | --- | --- | --- | --- | --- | --- | --- | --- | --- | --- | --- | --- | --- | --- | --- | --- | --- | --- | --- | --- | --- | --- | --- | --- | --- | --- | --- | --- | --- | --- | --- | --- | --- | --- | --- | --- | --- | --- | --- | --- | --- | --- | --- | --- | --- | --- | --- | --- | --- | --- | --- | --- | --- | --- | --- | --- | --- | --- | --- | --- | --- | --- | --- | --- | --- | --- | --- | --- | --- | --- | --- | --- | --- | --- | --- | --- | --- | --- | --- | --- | --- | --- |


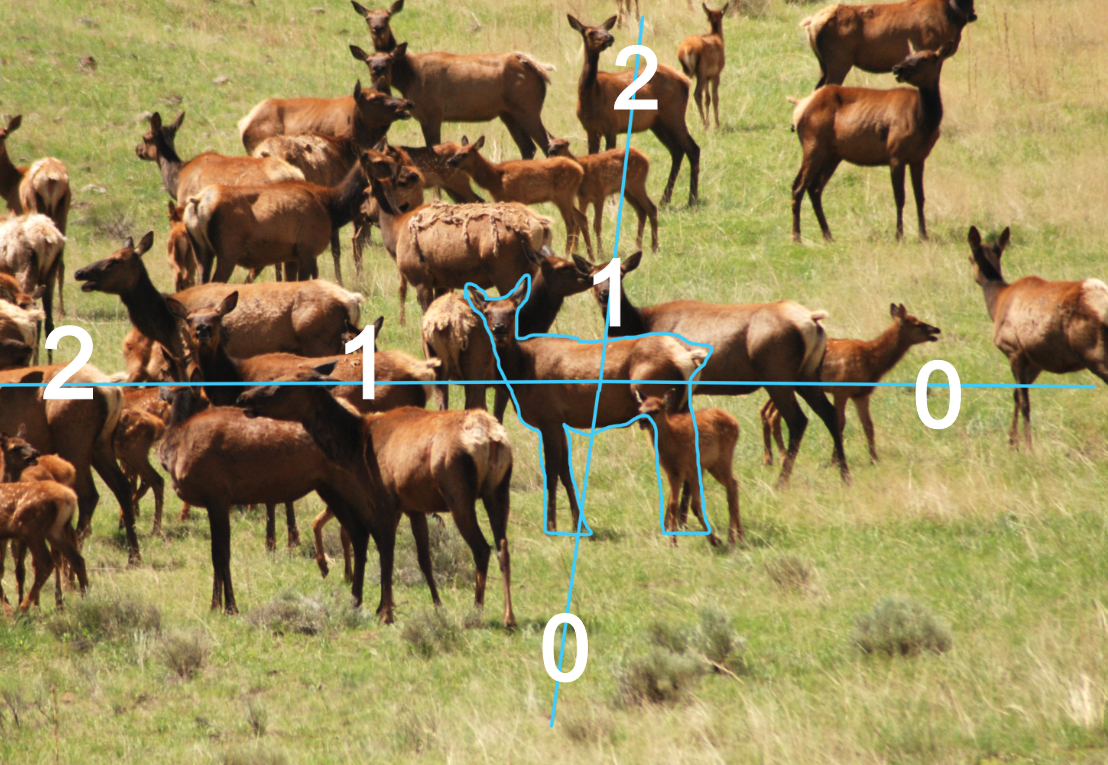


A


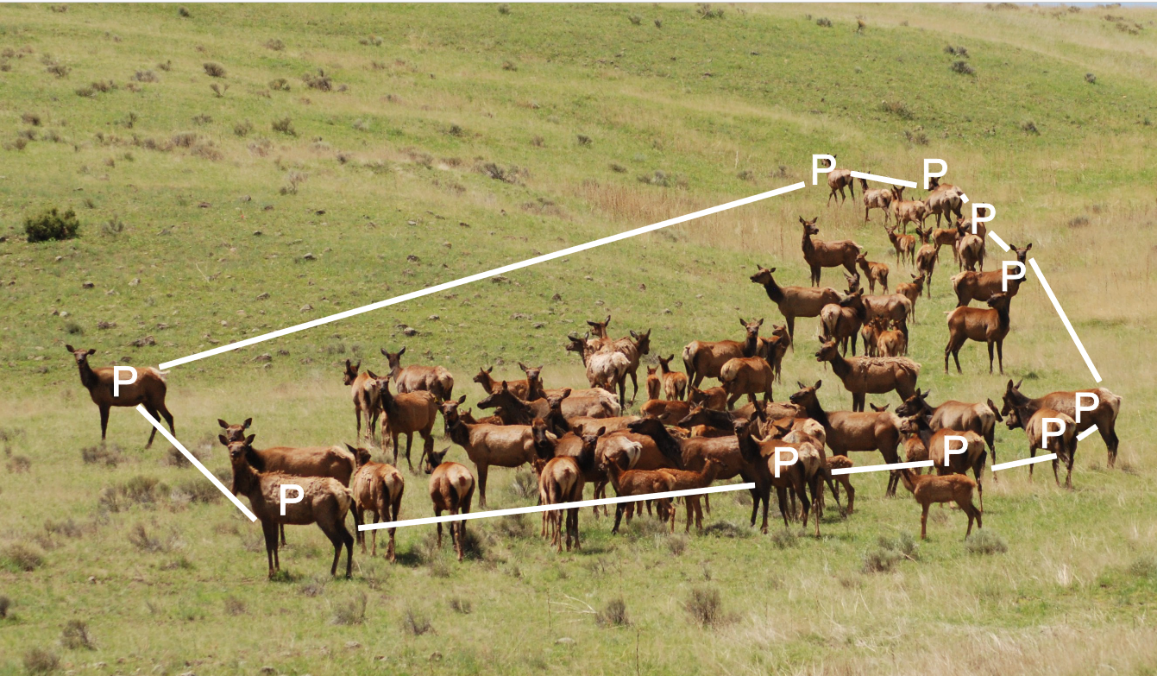


B

Figure A1. (A) Example of counting the number of neighboring elk on four sides of focal individual to the edge of the herd and (B) example of a circumscribing line (white) defining peripheral elk in a herd (labeled P). Calves were not counted as peripheral animals (Photos by J. Olson).


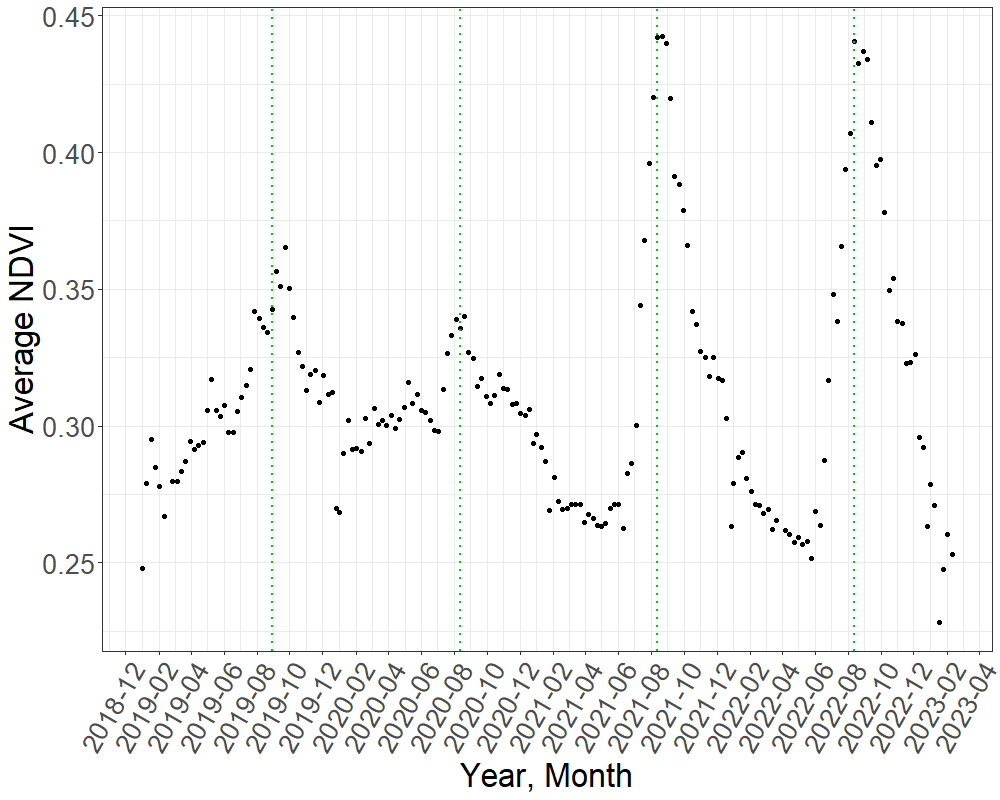


Figure A2. Average NDVI values across east-central Arizona and west-central New Mexico in 2019-2023. Dashed vertical lines represent approximate yearly maximum values.

SUPPLEMENTAL MATERIAL 2

MEXICAN WOLF PRESENCE INDEX AND ACTIVITY METRIC

*Analysis*

Thompson et al. (2025) developed third-order habitat selection functions for Mexican wolves (HSFs; Manly et al. 2002) using GPS collar locations collected from 137 wolves from January 2017 to June 2021. Collars with 1-2 hour fix rates were resampled to 6-hour fixes because the majority of wolf collars had fix intervals of 6- or 13-hours. Wolf location clusters were identified and only the first point from each cluster was retained to prevent bias towards den, rendezvous, bedding, and kill sites. Locations from individuals exhibiting transitory or dispersal movements, individuals with data for less than 60% of a season, and locations from within six days after a capture event or one day prior to an individual’s death or collar failure were removed. These cleaned data were used to create Utilization Distributions (UDs) as kernel density estimates (KDEs) in the R package *adehabitatHR* for wolf packs and the combined population across seasons and years, using the *href* smoothing method and a grid size of 3000 (Figure B1; Calenge 2006). We combined the points used to create these distributions with more recent GPS collar data collected from 129 wolves between June 2021 and June 2023. We cleaned these data using the same methods to create updated global, yearly, and seasonal UDs at the population level to represent long-term wolf risk and for each unique pack weighted by average and maximum pack size, and to update seasonal HSFs (Figure B2). Pack sizes were provided by the Mexican wolf IFT.

We derived random alternative points at a 10:1 available to used ratio from 100% minimum convex polygon (MCP) of the wolf population with bodies of water and slopes ≥ 70% excluded. Habitat covariates including elevation, slope, aspect, VRM, TRI, TPI, dominant vegetation type, vegetation cover, vegetation height, distance to forest cover, canopy cover, percent of open canopy pixels, distance to water bodies, human density, distance to recreation sites or trails, distance to roads of three types, densities of roads of three types, burn severity, and years since last fire were extracted at each point (Supplementary Material 1 – Table A2). We tested covariates for multicollinearity and scaled and centered the continuous covariates around zero. We then constructed *a priori* seasonal mixed logistic regression models with a random term for wolf pack (Table B1). We identified the top-performing models for each season by AIC score and validated them using 10-fold cross validation with 100 repetitions.

We also used generalized additive mixed-effect models (GAMMs) to predict the seasonal activity rates of wolves across the diel cycle in 2-hour increments (Kohl et al. 2018, Thompson et al. 2025). Out of Mexican wolf locations that were produced during the same time periods as observations were collected (2022-2023), we filtered the data to fix rates of two hours or less and resampled these data to a uniform two-hour fix rate, removed spatial autocorrelation among individuals, and removed dispersal movements. We then calculated the distance traveled in kilometers between successive points for individuals and modeled GAMMs seasonally on a negative binomial distribution as a function of time of day and year with individual wolf ID as a random effect. Using the resulting models, we predicted wolf activity estimates at the times of observations.

*Results*

Mexican wolves across seasons selected for areas closer to water bodies, recreation sites and trails, but further from private land (Table B2; Figure B3). In monsoon season wolves selected for higher densities of moderate-use roads, and in all other seasons selected for areas closer to primitive roads. Wolves also selected for intermediate elevations and young burn sites (≤ 5 years) and against older burn sites (11-17 years) and moderate-age burn sites (6-10 years) (Figure B3). The addition of a covariate for diel period did not improve model AIC score. Spearman’s rho estimated from the 10-fold cross validation was 0.99 for the top model in each season.

Diel activity patterns of Mexican wolves remained relatively constant across years (2021-2023) and seasons and followed expected patterns, with movement rates peaking around dawn and dusk (Figure B4). There was some evidence of a third activity peak in late afternoon during monsoon season and fall as seen by Thompson (2022), but it was not as distinct as with previous years of data, and there were smaller activity peaks prior to midnight in spring and winter. The final seasonal activity models included 7,718 locations from 30 collared wolves.

*Literature cited*

Calenge, C. 2006. The package adehabitat for the R software: a tool for the analysis of space and habitat use by animals. Ecological Modelling 197:516–519.

Kohl, M. T., D. R. Stahler, M. C. Metz, J. D. Forester, M. J. Kauffman, N. Varley, P. J. White, D. W. Smith, and D. R. MacNulty. 2018. Diel predator activity drives a dynamic landscape of fear. Ecological Monographs 88:638–652.

Manly, B. F. J., L. L. McDonald, D. L. Thomas, T. L. McDonald, and W. P. Erickson. 2002. Resource selection by animals: statistical design and analysis for field studies. Second edition. Springer Science & Business Media.

Thompson, C. 2022. Elk habitat selection in response to predation risk from Mexican gray wolves. MS Thesis, New Mexico State University, Las Cruces, USA.

*Tables*

Table B1. A priori model structures tested for the Mexican habitat selection function using locations from GPS-collared Mexican wolves in east-central Arizona and west-central New Mexico between 2017-2023. All models included a random effect for unique pack ID.

| Model Structure | |
| --- | --- |
| 1 | Priv_D_ + El + El^2^ + R3_D_ + W_D_ + YSF |
| 2 | Priv_D_ + El + El^2^ + RT_D_ + W_D_ + YSF |
| 3 | Priv_D_ + El + El^2^ + W_D_ + Fire |
| 4 | Priv_D_ + El + El^2^ + RT_D_ + W_D_ + CC |
| 5 | Priv_D_ + El + CC + CC^2^ + W_D_ + RT_D_ |
| 6 | Priv_D_ + El + El^2^ + RT_D_ + CC + CC^2^ |
| 7 | Priv_D_ + El + El^2^ + RT_D_ + W_D_ + Slope |
| 8 | Priv_D_ + El + El^2^ + R3_D_ + W_D_ + Slope |
| 9 | Priv_D_ + El + El^2^ + RT_D_ + YSF + Slope |
| 10 | Priv_D_ + El + El^2^ + W_D_ + YSF + Slope |
| 11 | Priv_D_ + El + El^2^ + R3_D_ + YSF + Slope |
| 12 | El + El^2^ + RT_D_ + W_D_ + R3_D_ + YSF |
| 13 | Priv_D_ + El + El^2^ + R3_D_ + RT_D_ + YSF |
| 14 | Priv_D_ + El + El^2^ + R3_D_ + RT_D_ + W_D_ + YSF |
| 15 | RT_D_ + El + El^2^ + W_D_ + Fire |
| 16 | Priv_D_ + El + El^2^ + W_D_ + CC + CC*El |
| 17 | Priv_D_ + El + El^2^ + W_D_ + CC + CC*El^2^ |
| 18 | Priv_D_ + El + El^2^ + W_D_ + Diel + Priv_D_*Diel |
| 19 | Priv_D_ + El + El^2^ + W_D_ + R2_DEN_ + RT_D_ |
| 20 | Priv_D_ + El + El^2^ + W_D_ + R3_D_ + RT_D_ |
| 21 | Priv_D_ + El + El^2^ + W_D_ + Diel + Diel*El |
| 22 | Priv_D_ + El + El^2^ + RT_D_ + Diel + Diel*RT_D_ |
| 23 | Priv_D_ + El + El^2^ + W_D_ + Diel + Diel*El |
| 24 | Priv_D_ + El + El^2^ + RT_D_ + W_D_ + Burn |
| 25 | Priv_D_ + El + El^2^ + RT_D_ + W_D_ + Burn + Diel + Burn*Diel |
| 26 | Priv_D_ + El + El^2^ + RT_D_ + W_D_ + R3_DEN_ + YSF |
| 27 | Priv_D_ + El + R3_D_ + RT_D_ + W_D_ + Burn |
| 28 | Priv_D_ + Veg + W_D_ + Fire |
| 29 | Priv_D_ + Veg + W_D_ + Fire + RT_D_ |
| 30 | Priv_D_ + Veg + W_D_ + RT_D_ + Diel + Priv_D_*Diel |
| 31 | Priv_D_ + El + El^2^ + RT_D_ + W_D_ + R2_DEN_ + YSF |
| 32 | Priv_D_ + El + El^2^ + RT_D_ + W_D_ + Open_500_ |
| 33 | Human_DEN_ + El + El^2^ + W_D_ + Diel + Human_DEN_*Diel |
| 34 | Human_DEN_ + El + El^2^ + RT_D_ + W_D_ + YSF |
| 35 | Priv_D_ + El + Open_500_ + Open_500_^2^ + W_D_ + RT_D_ |
| 36 | Priv_D_ + El + El^2^ + Open_500_ + Open_500_^2^ + RT_D_ |
| 37 | Priv_D_ + El + El^2^ + Open_500_ + W_D_ + Open_500_*El |
| 38 | Priv_D_ + El + El^2^ + Open_500_ + Diel + Open_500_*Diel |
| 39 | W_D_ + El + El^2^ + Open_500_ + Diel + Open_500_*Diel |
| 40 | RT_D_ + El + El^2^ + W_D_ + R3_D_ + Human_DEN_ |

Model terms: Priv_D_ = distance to private land; El = elevation; R_D_ = distance to road classes 1, 2, 3 or all classes; W_D_ = distance to natural and artificial water bodies; YSF = years since most recent fire (reference = > 17 years/unburned); RT_D_ = distance to recreation sites and trails; Fire = fire history combining burn severity and time since fire (reference = > 17 years/unburned); CC = percent canopy cover; Diel = diel period; R_DEN_ = density of road classes 1, 2, 3, or all classes; Burn = burned in past 17 years (reference = unburned); Veg = dominant vegetation type (reference = grassland); Open_500_ = number of open pixels in a 500 m rolling window; Human_DEN_ = human population density.

Table B2. Model comparisons by season for Mexican wolf HSFs in east central Arizona and west-central New Mexico between 2017-2023, showing the five top-ranked models for each period. All models included a random intercept for individual ID. K = degrees of freedom, AIC = Akaike’s Information Criterion score, ΔAIC = difference in AIC score relative to the top-ranked model, *w* = individual model weight, and LL = log likelihood.

| **Model** | **K** | **AIC_C_** | **ΔAIC_C_** | ***w*** | **LL** |
| --- | --- | --- | --- | --- | --- |
| **Fall** | | | | | |
| Priv_D_ + El + El^2^ + RT_D_ + W_D_ + R3_D_ + YSF | 11 | 172561.45 | 0 | 1.00 | -86269.73 |
| Priv_D_ + El + El^2^ + RT_D_ + W_D_ + R2_DEN_ + YSF | 11 | 172694.88 | 133.42 | 0 | -86336.44 |
| Priv_D_ + El + El^2^ + RT_D_ + W_D_ + R3_DEN_ + YSF | 11 | 172760.80 | 199.34 | 0 | -86369.40 |
| Priv_D_ + El + El^2^ + RT_D_ + W_D_ + YSF | 10 | 172975.09 | 413.63 | 0 | -86477.54 |
| Priv_D_ + El + El^2^ + W_D_ + Slope + YSF | 10 | 173559.78 | 998.32 | 0 | -86769.89 |
| **Winter** | | | | | |
| Priv_D_ + El + El^2^ + RT_D_ + W_D_ + R3_D_ + YSF | 11 | 431309.80 | 0 | 1.00 | -215643.9 |
| Priv_D_ + El + El^2^ + RT_D_ + W_D_ + R3_DEN_ + YSF | 11 | 432586.37 | 1276.57 | 0 | -216282.19 |
| Priv_D_ + El + El^2^ + RT_D_ + W_D_ + R2_DEN_ + YSF | 11 | 433433.47 | 2123.67 | 0 | -216705.73 |
| Priv_D_ + El + El^2^ + RT_D_ + W_D_ + YSF | 10 | 433540.80 | 2231.00 | 0 | -216760.40 |
| Priv_D_ + El + El^2^ + R3_D_ + W_D_ + YSF | 10 | 433817.76 | 2507.96 | 0 | -216898.88 |
| **Spring** | | | | | |
| Priv_D_ + El + El^2^ + RT_D_ + W_D_ + R3_D_ + YSF | 11 | 362800.82 | 0 | 1.00 | -181389.41 |
| Priv_D_ + El + El^2^ + RT_D_ + W_D_ + R3_DEN_ + YSF | 11 | 363690.26 | 889.44 | 0 | -181834.13 |
| Priv_D_ + El + El^2^ + RT_D_ + W_D_ + R2_DEN_ + YSF | 11 | 363911.34 | 1110.52 | 0 | -181944.67 |
| Priv_D_ + El + El^2^ + RT_D_ + W_D_ + YSF | 10 | 364007.61 | 1206.79 | 0 | -181993.80 |
| Priv_D_ + El + El^2^ + R3_D_ + W_D_ + YSF | 10 | 364202.86 | 1402.04 | 0 | -182091.43 |
| **Monsoon** | | | | | |
| Priv_D_ + El + El^2^ + RT_D_ + W_D_ + R2_D_ + YSF | 11 | 239635.43 | 0 | 1.00 | -119806.72 |
| Priv_D_ + El + El^2^ + RT_D_ + W_D_ + R3_D_ + YSF | 11 | 239693.71 | 58.28 | 0 | -119835.85 |
| Priv_D_ + El + El^2^ + RT_D_ + W_D_ + R3_DEN_ + YSF | 11 | 239695.45 | 60.01 | 0 | -119836.72 |
| Priv_D_ + El + El^2^ + RT_D_ + W_D_ + YSF | 10 | 239799.00 | 163.57 | 0 | -119889.50 |
| Priv_D_ + El + El^2^ + W_D_ + Slope + YSF | 10 | 239910.67 | 275.24 | 0 | -119945.34 |

Model terms: Priv_D_ = distance to private land; El = elevation; RT_D_ = distance to recreation sites and trails; W_D_ = distance to natural and artificial water bodies; R_D_ = distance to types 2 or 3 roads; R_DEN_ = density of types 2 or 3 roads; YSF = years since most recent fire (reference = > 17 years/unburned).

*Figures*


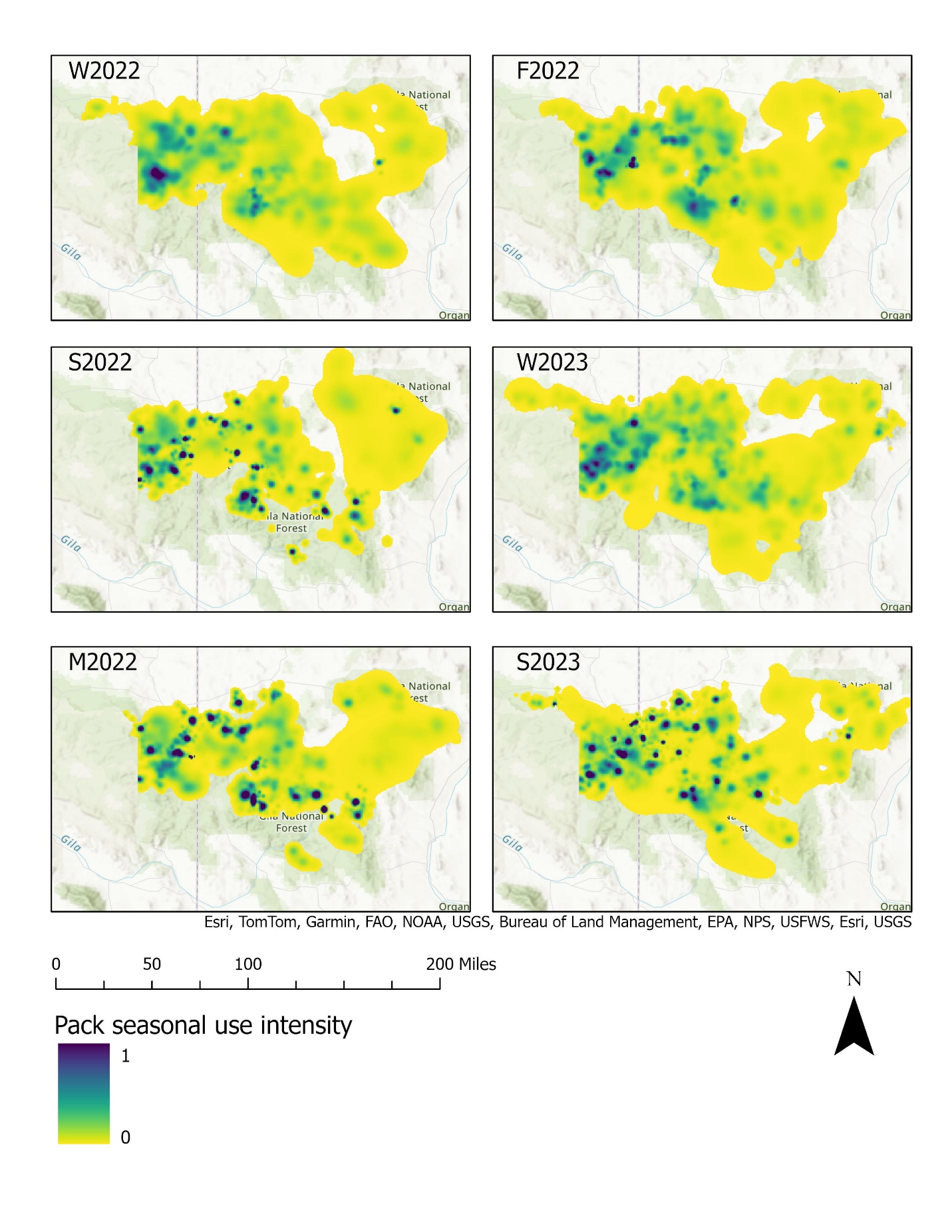


Figure B1. Mexican wolf pack UDs partitioned seasonally into winter (W), spring (S), monsoon M), and fall (F) between 2022-2023 in east-central Arizona and west-central New Mexico with dark blue representing higher intensities of use. Data from tribal lands are excluded from figures based on an agreement between local tribes and USFWS.

*
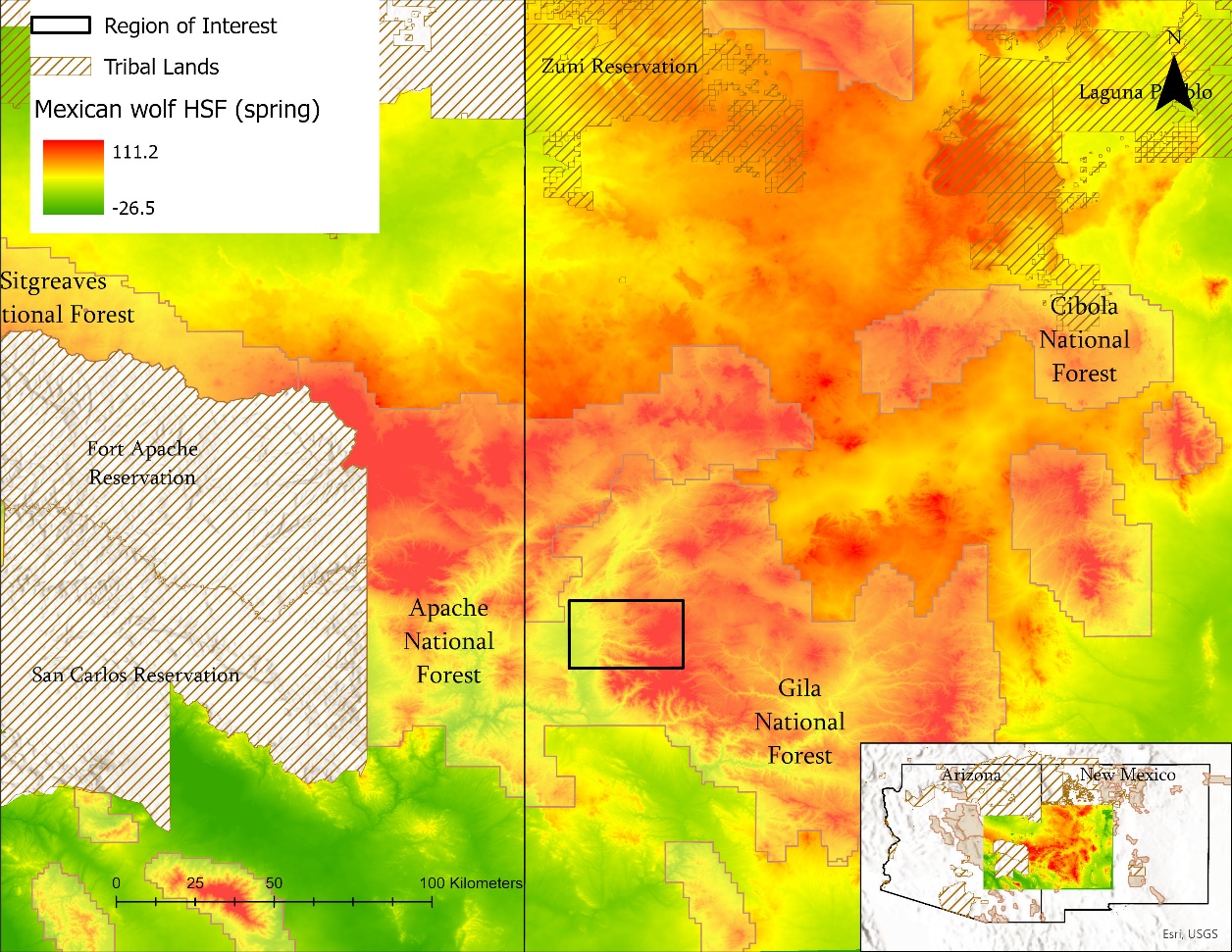
*

*
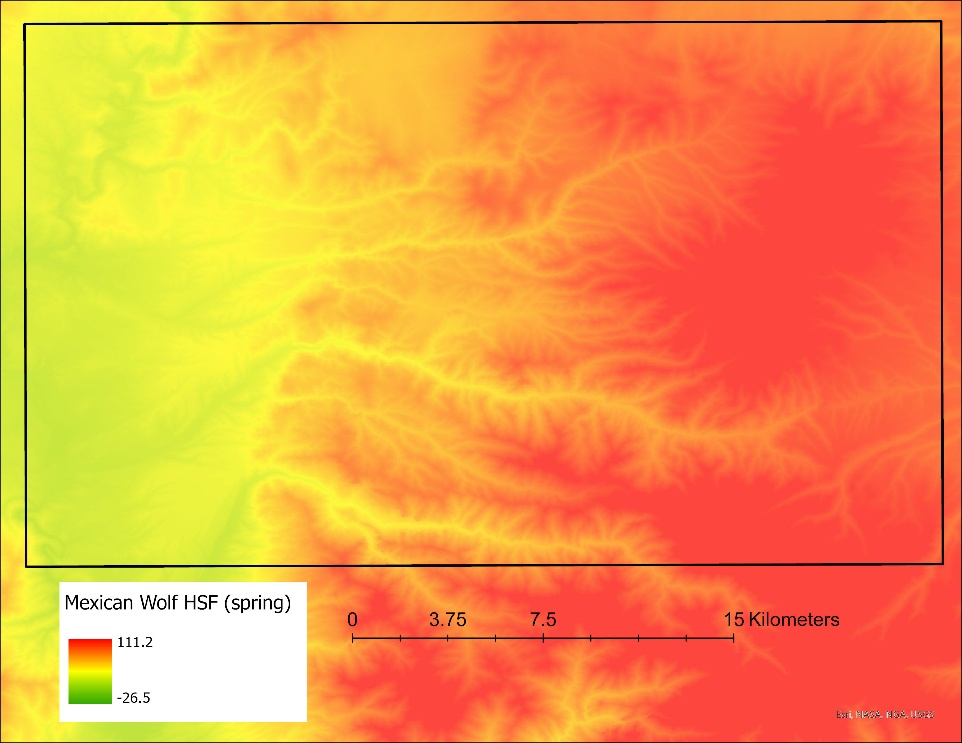
*

Figure B2. Mexican wolf spring habitat selection function (HSF) for 2017-2023 in east-central Arizona and west-central New Mexico with red indicating higher probabilities of selection and green indicating lower probabilities of selection. The region of interest shows a portion of the study area in the Gila National Forest, NM. Data from tribal lands are excluded from figures based on an agreement between local tribes and USFWS.


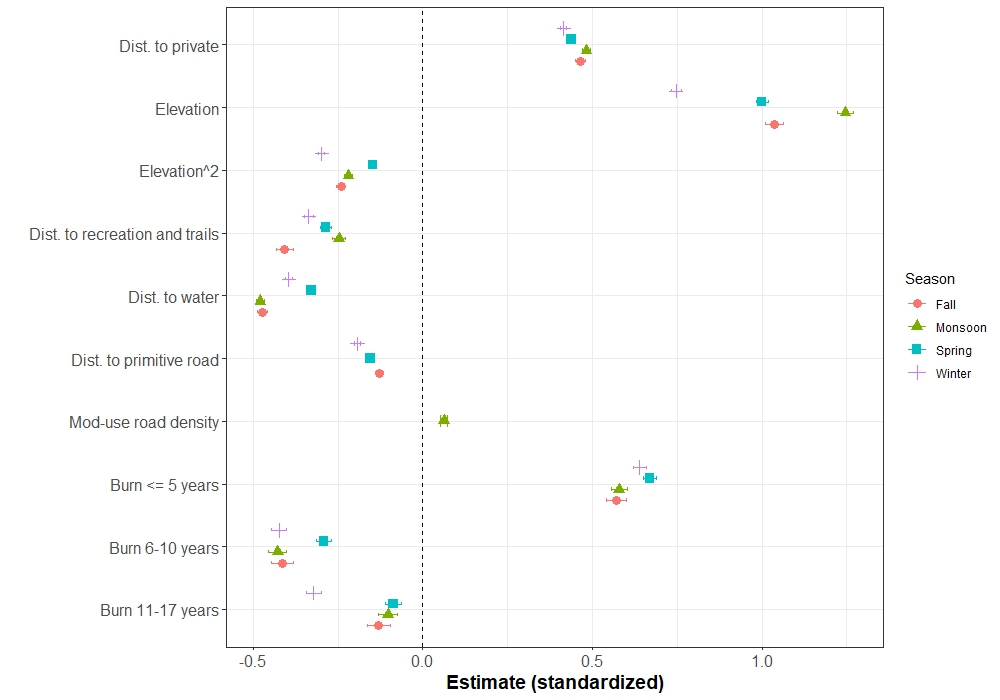


Figure B3. Standardized regression coefficients with 95% confidence intervals for top seasonal HSFs for Mexican wolves in west-central Arizona and east-central New Mexico between 2017-2023. The reference category used for burn age was unburned within 17 years.


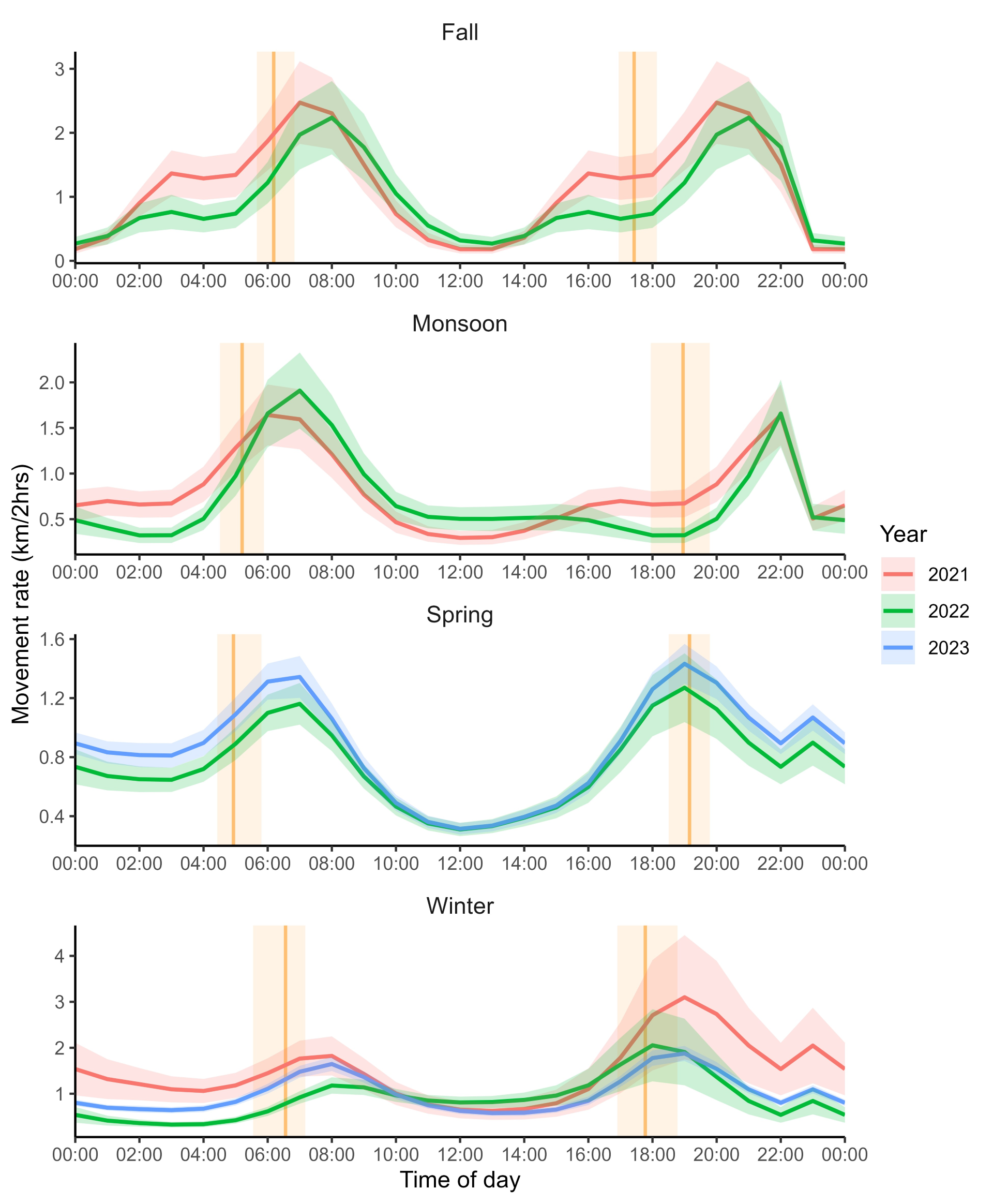


Figure B4. Diel patterns of movement rates (km/2 hr) of Mexican wolves in the MWEPA between Fall 2021 and Spring 2023 with 95% CI. Seasonal civil twilight is shown in vertical bands with the average shown in the darker vertical line.

SUPPLEMENTAL MATERIAL 3

MOUNTAIN LION PRESENCE INDEX

*Analysis*

We used GPS locations from 23 collared mountain lions captured in New Mexico between April 2021 and June 2023 to construct seasonal and diel habitat selection functions (HSFs). Lions were captured and collared by NMDGF. All GPS collars were set to 1-hour fix rates year-round, and these data were resampled to 3-hour fix rates to reduce the spatial and temporal autocorrelation common to GPS location data. We additionally identified all location clusters using the R package *GPSeqClus* (Clapp et al. 2021, Cluff and Mech 2023; see Supplementary Material 4 for cluster identification details) and retained only the first point from each cluster. Individuals with data for less than 60% of a season were removed from that season. We checked individual movement trajectories for exploratory or dispersal movements, but no lions in this sample exhibited significant dispersal movements. This resulted in 24,894 used locations after data cleaning.

To construct individual home ranges for mountain lions, we kept only collar points at least five days after an individual’s capture or recollar date and one day before their known death or collar failure. We calculated kernel density estimates (KDEs) for each individual and extracted polygons for the 99% isopleth using the R package *adehabitatHR* with the *ad hoc* smoothing method (Calenge 2006). We also constructed home range subsets for the collared individuals using only locations from the time that we were actively following those individuals to document kill sites (see Supplementary Material 5). We buffered each individual lion’s home range by the average distance lions travelled in one hour (712 m) and removed perennial lakes as these are not available areas to mountain lions. We then sampled random locations at 20:1 ratio of available to used points from within each individual mountain lion’s home range polygon and ensured that no duplicate locations existed, targeting within-home-range habitat selection by individual lions (Manly et al. 2002).

We extracted the dominant vegetation class (oak/shrubland, aspen, piñon-juniper, ponderosa, grassland, mixed conifer, wet meadow/pasture, and other) and reclassified the vegetation type “other” as grassland given that it comprised primarily grassy riparian areas. We combined aspen with mixed conifer areas due to the small sample size in aspen and assigned grassland as the reference category because it was the most ubiquitous vegetation across the study area. We also extracted vegetation height and reclassified it into a binary variable representing unobstructed views for an elk (vegetation < 1 m or trees > 5 m) or obstructed view (vegetation and shrubs > 1 m or trees between 1-5 m), as well as vegetation cover class and distance to an ecotone (defined as a transition between treed and open habitat buffered by 50 m) from rasters developed by Thompson (2022) using LANDFIRE (2016) datasets. We extracted percent canopy cover from National Land Cover Database tree canopy data (MRLC 2021) and created a multi-scale measure of openness by reclassifying pixels as open (< 30% canopy cover) or closed (30% or greater canopy cover) and subsequently summing the number of open pixels in rolling windows of sizes 90, 240, and 480 m around a focal pixel. We extracted topographic measurements including elevation, slope, aspect, ruggedness (Vector Ruggedness Measure and Topographic Ruggedness Index), and topographic position (TPI) using a Digital Elevation Model obtained from LANDFIRE (2016), as well as the distance to and 1.5 km^2^ density of road types 1 (high-use gravel or paved roads accessible to low-clearance vehicles), 2 (moderate-use dirt or gravel roads that may require moderate clearance vehicles), and 3 (low-use, unmaintained dirt roads requiring high-clearance vehicles), distance to recreation sites and trails, human population density, and distance to natural and artificial water bodies using distance and density rasters created by Thompson (2022).

To create multiple measures of fire history, we first obtained wildfire perimeter polygons from the National Interagency Fire Center (NIFC 2023) and classified years since the most recent fire at each observation location as 0 (unburned within the past 17 years), 1 (burned within 0-5 years), 2 (burned within 5-13 years), or 3 (burned within 13-17 years). We combined burn severity data from both the Monitoring Trends in Burn Severity (MTBS 2021) and Burned Area Emergency Response (BAER 2022) databases and reclassified these data as 0 (unburned or increased greenness), 1 (low-severity), 2 (moderate severity), and 3 (high-severity). We converted the burn severity metric to a continuous variable as the classes were ordinal, tested a binary burn variable representing areas that burned within the past 17 years, and created a combined categorical metric of fire history using both time since fire and burn severity as 0 (unburned or burned with increased greenness within past 17 years), 1 (low-severity burn within 5 years), 2 (moderate/high severity burn within 5 years), 3 (low-severity burn within 6-17 years), and 4 (moderate/high severity burn within 6-17 years). The combined metric was intended to provide a complete picture of fire history given that the effects of fire on vegetation structure and animal behavior depend on both severity and time since fire (Jin et al. 2012, Cherry et al. 2017, Williamson and Weckerly 2020).

We assessed all linear covariates for collinearity using a Spearman’s rank correlation test. If any covariates were collinear with each other (*r* ≥ 0.6), we did not include them in the same model and instead either selected the most ecologically relevant variable of a collinear group or swapped out collinear predictors and retained the one that explained the greatest amount of variance according to Akaike’s Information Criterion (AIC) score when both were equally ecologically relevant. Prior to modeling, we resampled each individual’s random alternative locations from 20 to 10 times the number of used locations, created seasonal and seasonal-diel subsets of the data, and centered and scaled the complete data and its subsets. We assigned data to two diel periods (day and night) based on average seasonal activity rates modeled with generalized additive mixed models (GAMMs) following methods used by Thompson (2022) and Kohl et al. (2018) and civil twilight times (Figure C1). We then constructed 26 *a priori* mixed logistic regression models based on a literature review of mountain lion habitat selection studies in similar ecosystems (Table C1; Beier et al. 1995, Nicholson et al. 2014, Robinson et al. 2015, Benson et al. 2016, Blake and Gese 2016, Dellinger et al. 2020, Peterson et al. 2021) and modeled them on the seasonal and seasonal-diel subsets to determine whether model rankings differed between diel periods and seasons. Because the purpose of these models was to provide the best predictive measure of mountain lion habitat use, we also removed uninformative parameters and parameters with high Variance Inflation Factor (VIF) scores and selected the top model for each data subset based on AIC score. We assessed the performance of the top models using 10-fold cross validation with 50 repetitions and 20 bins (Boyce et al. 2002). Additionally, we tested 3 additional available:used point ratios (5:1, 15:1, 20:1) to ensure that coefficient estimates were stable at different ratios.

To create a more robust measure of mountain lion presence, we also used kernel density estimators (KDEs) to construct probability density functions across years and seasons as described for Mexican wolves in Supplementary Material 2 (Figure C2; Hebblewhite and Merrill 2007). Unlike the Mexican wolf population in this study area, in which approximately 60% of known individuals are collared and at least one collar is deployed in most wolf packs, the proportion of the mountain lion population that were fitted with GPS collars is unknown due to a lack of population abundance or density estimates for the project area.

*Results*

Due to significant differences in coefficient estimates across seasonal and diel periods, we selected top models for each seasonal-diel period. Across models, VIF scores were less than 2 for all model terms that were not included in an interaction. These models performed well with 10-fold cross validations resulting in Spearman’s rho values ranging from 0.987-0.994 across seasonal-diel models. Mountain lions selected for moderately rugged terrain, higher topographic position indices, obstructive vegetation taller than 1 m, and areas closer to forest edges across seasons and diel periods, though effect sizes did vary (Figure C3 & C4). Lions selected for intermediate elevations during both fall diel periods, for higher elevation during both spring and monsoon diel periods, and for lower elevation during both winter diel periods. In all diel periods of fall, winter, and spring, lions selected for lower densities of all road types while in monsoon season road density had no effect, and in all diel periods of fall, spring, and monsoon season lions selected for increased canopy cover while in winter canopy cover had no effect (Figure C4). Lions selected areas closer to water in fall and monsoon season, but further from water in spring and did not exhibit significant selection for water in winter. Lions also selected against north-facing slopes in fall and winter, but northness had no effect on selection in spring and monsoon season (Figure C4). The top models for both winter diel periods also included dominant vegetation type and distance to recreation sites and trails, with lions selecting for oak/shrubland, pinon-juniper, ponderosa, and mixed conifer compared to grasslands and pastures, and for closer distances to recreation sites/trails (Figure C4).

In nearly all diel periods of all seasons, lions selected against areas burned at low severity within the past five years compared to unburned areas, though the effect was insignificant during the day in winter (Figure C4). In most seasons and diel periods percent canopy cover did not influence the effect of this burn category, though during the day in monsoon season lions selected for less canopy cover when in these low severity, recent burns. In both diel periods of monsoon season and winter and during the day in spring, lions strongly selected against moderate-to-high severity burns that occurred within the past five years. However, lions did select for increased canopy cover during the day in spring and during the night in winter while in moderate-to-high severity, recent burns. Lions selected against low-severity burns that occurred within the past 6-17 years during all seasons and diel periods, and for lower canopy cover when in these areas during both diel periods in spring and winter, and at night in fall and monsoon season. Finally, lions strongly selected against moderate-to-high severity burns that occurred within the past 6-17 years during spring and winter, and for lower canopy cover when in these areas during both diel periods in monsoon season, at night in spring, and during the day in fall.

*Literature Cited*

Beier, P., D. Choate, and R. H. Barrett. 1995. Movement patterns of mountain lions during different behaviors. Journal of Mammalogy 76:1056–1070.

Benson, J. F., J. A. Sikich, and S. P. D. Riley. 2016. Individual and population level resource selection patterns of mountain lions preying on mule deer along an urban-wildland gradient. D. Russo, editor. PLOS ONE 11:e0158006.

Blake, L. W., and E. M. Gese. 2016. Resource selection by cougars: Influence of behavioral state and season: Cougar Resource Selection. Journal of Wildlife Management 80:1205–1217.

Börger, L., N. Franconi, G. De Michele, A. Gantz, F. Meschi, A. Manica, S. Lovari, and T. Coulson. 2006. Effects of sampling regime on the mean and variance of home range size estimates. Journal of Animal Ecology 75:1393–1405.

Boyce, M. S., P. R. Vernier, S. E. Nielsen, and F. K. A. Schmiegelow. 2002. Evaluating resource selection functions. Ecological Modelling 157:281–300.

Burned Area Emergency Response (BAER). 2022. Burned Area Emergency Response Imagery Support. <https://burnseverity.cr.usgs.gov/baer/baer-imagery-support-data-download>. Accessed 18 Sep 2023.

Calenge, C. 2006. The package adehabitat for the R software: a tool for the analysis of space and habitat use by animals. Ecological Modelling 197:516–519.

Cherry, M. J., R. J. Warren, and L. M. Conner. 2017. Fire‐mediated foraging tradeoffs in white‐tailed deer. Ecosphere 8:e01784.

Clapp, J. G., J. D. Holbrook, and D. J. Thompson. 2021. GPSeqClus: An R package for sequential clustering of animal location data for model building, model application and field site investigations. S. Windecker, editor. Methods in Ecology and Evolution 12:787–793.

Cluff, H. D., and L. D. Mech. 2023. A field test of R package GPSeqClus: For establishing animal location clusters. Ecological Solutions and Evidence 4:e12204.

Dellinger, J. A., B. Cristescu, J. Ewanyk, D. J. Gammons, D. Garcelon, P. Johnston, Q. Martins, C. Thompson, T. W. Vickers, C. C. Wilmers, H. U. Wittmer, and S. G. Torres. 2020. Using mountain lion habitat selection in management. Journal of Wildlife Management 84:359–371.

Hebblewhite, M., and E. H. Merrill. 2007. Multiscale wolf predation risk for elk: does migration reduce risk? Oecologia 152:377–387.

Jin, Y., J. T. Randerson, S. J. Goetz, P. S. A. Beck, M. M. Loranty, and M. L. Goulden. 2012. The influence of burn severity on postfire vegetation recovery and albedo change during early succession in North American boreal forests. Journal of Geophysical Research: Biogeosciences 117.

Kohl, M. T., D. R. Stahler, M. C. Metz, J. D. Forester, M. J. Kauffman, N. Varley, P. J. White, D. W. Smith, and D. R. MacNulty. 2018. Diel predator activity drives a dynamic landscape of fear. Ecological Monographs 88:638–652.

LANDFIRE. 2016. Existing Vegetation Type (EVT), National Vegetation Classification (NVC), Existing Vegetation Cover (EVC), Existing Vegetation Height (EVH). <https://landfire.gov/>. Accessed 11 Oct 2020.

Manly, B. F. J., L. L. McDonald, D. L. Thomas, T. L. McDonald, and W. P. Erickson. 2002. Resource selection by animals: statistical design and analysis for field studies. Second edition. Springer Science & Business Media.

Monitoring Trends in Burn Severity (MTBS). 2021. Burn Severity Mosaics. <https://www.mtbs.gov/direct-download>. Accessed 14 Sep 2023.

Multi-Resolution Land Characteristics Consortium (MRLC). 2021. NLCD 2021 Tree Canopy Cover (CONUS). <https://www.mrlc.gov/data/nlcd-2021-tree-canopy-cover-conus>. Accessed 24 Sep 2023.

National Interagency Fire Center (NIFC). 2023. WFIGS Interagency Fire Perimeters. <https://data-nifc.opendata.arcgis.com/datasets/nifc::wfigs-interagency-fire-perimeters/explore>. Accessed 22 May 2023.

Nicholson, K. L., P. R. Krausman, T. Smith, W. B. Ballard, and T. McKinney. 2014. Mountain lion habitat selection in Arizona. The Southwestern Naturalist 59:372–380.

Nilsen, E. B., S. Pedersen, and J. D. C. Linnell. 2008. Can minimum convex polygon home ranges be used to draw biologically meaningful conclusions? Ecological Research 23:635–639.

Peterson, C. J., M. S. Mitchell, N. J. DeCesare, C. J. Bishop, and S. S. Sells. 2021. Habitat selection by wolves and mountain lions during summer in western Montana. W. D. Walter, editor. PLOS ONE 16:e0254827.

Robinson, H. S., T. Ruth, J. A. Gude, D. Choate, R. DeSimone, M. Hebblewhite, K. Kunkel, M. R. Matchett, M. S. Mitchell, K. Murphy, and J. Williams. 2015. Linking resource selection and mortality modeling for population estimation of mountain lions in Montana. Ecological Modelling 312:11–25.

Thompson, C. 2022. Elk habitat selection in response to predation risk from Mexican gray wolves. MS Thesis, New Mexico State University, Las Cruces, USA.

Williamson, L. H., and F. W. Weckerly. 2020. Elk forage response to prescribed fire in Boyes meadow, Prairie Creek Redwoods State Park, California. California Fish and Wildlife Journal 106: 93-102.

*Tables*

Table C1. A priori model structures evaluated for mountain lion seasonal/diel HSFs in west-central New Mexico between 2021 and 2023. All models included a random intercept for individual ID.

| Model Structure | |
| --- | --- |
| 1 | TRI + TRI^2^ + TPI + AllR_DEN_ + CC + TPI*CC + AllR_DEN_*CC |
| 2 | TRI + TRI^2^ + TPI + AllR_DEN_ + CC + VH + TPI*VH + AllR_DEN_*VH |
| 3 | El + El^2^ + TRI + TRI^2^ + AllR_DEN_ + W_D_ + N + Veg |
| 4 | Human_DEN_ + Veg |
| 5 | AllR_DEN_ + Veg |
| 6 | El + TRI + TRI^2^ + AllR_DEN_ + CC + VH |
| 7 | El + El^2^ + TRI + TRI^2^ + AllR_DEN_ + Fire |
| 8 | El + TRI + TRI^2^ + AllR_DEN_ + Fire |
| 9 | El + TRI + TRI^2^ + AllR_DEN_ + Fire + Fire*AllR_DEN_ |
| 10 | El + TRI + TRI^2^ + Edge_D_ + Fire |
| 11 | El + TRI + TRI^2^ + Human_DEN_ |
| 12 | El + TRI + TRI^2^ + Human_DEN_ + Fire |
| 13 | El + TRI + TRI^2^ + TPI + AllR_DEN_ + CC + W_D_ + VH + N + Edge_D_ + Fire + AllR_DEN_*VH |
| 14 | El + TRI + TRI^2^ + TPI + AllR_DEN_ + CC + W_D_ + VH + N + Edge_D_ + Fire + CC*Fire |
| 15 | El + El^2^ + TRI + TRI^2^ + AllR_DEN_ + W_D_ + N + Veg + RT_D_ |
| 16 | El + El^2^ + TRI + TRI^2^ + AllR_DEN_ + W_D_ + N + Veg + RT_D_ + Fire |
| 17 | El + TRI + TRI^2^ + AllR_DEN_ |
| 18 | El + AllR_DEN_ + TPI + W_D_ |
| 19 | TRI + TRI^2^ + AllR_DEN_ + W_D_ + VH + AllR_DEN_*VH |
| 20 | El + TRI + TRI^2^ + TPI + AllR_DEN_ + CC + W_D_ + VH + N + Edge_D_ + Fire |
| 21 | El + El^2^ + TRI + TRI^2^ + TPI + AllR_DEN_ + CC + W_D_ + VH + N + Edge_D_ + Fire + CC*Fire |
| 22 | El + El^2^ + TRI + TRI^2^ + TPI + AllR_DEN_ + CC + W_D_ + VH + N + Edge_D_ + Fire |
| 23 | El + El^2^ + TRI + TRI^2^ + TPI + AllR_DEN_ + Veg + RT­_D_ + N + Edge_D_ + Fire |
| 24 | El + El^2^ + TRI + TRI^2^ + TPI + AllR_DEN_ + CC + Veg + RT­_D_ + N + Edge_D_ + Fire + CC*Fire |
| 25 | El + TRI + TRI^2^ + TPI + AllR_DEN_ + CC + W_D_ + VH + N + Edge_D_ + Fire |
| 26 | El + TRI + TRI^2^ + TPI + AllR_DEN_ + CC + W_D_ + VH + N + Edge_D_ + Fire + CC*Fire |
| 27 | El + El^2^ + TRI + TRI^2^ + TPI + CC + W_D_ + VH + Edge_D_ + Fire |

Model terms: TRI = topographic ruggedness index; TPI = topographic position index; AllR_DEN_ = density of all road classes; CC = percent canopy cover; VH = classified vegetation height (reference = 0-1 m); El = elevation; W_D_ = distance to natural and artificial water bodies; N = northness index; Veg = dominant vegetation type (reference = grassland); Human_DEN_ = human density; Fire = fire history class (reference = unburned in over 17 years); Edge_D_ = distance to forest edge; RT_D_ = distance to recreation sites and trails.

Table C2. Model comparisons by season and diel period for mountain lion HSFs in west-central NM between 2021 and 2023, showing the five top-ranked models for each period. All models included a random intercept for individual ID. K = degrees of freedom, AIC = Akaike’s Information Criterion score, ΔAIC = difference in AIC score relative to the top-ranked model, *w* = individual model weight, and LL = log likelihood.

| **Model** | **K** | **AIC** | **ΔAIC** | ***w*** | **LL** |
| --- | --- | --- | --- | --- | --- |
| **Fall: Day** | | | | | |
| El + El^2^ + TRI + TRI^2^ + TPI + AllR_DEN_ + CC + W_D_ + VH + N + Edge_D_ + Fire + CC*Fire | 21 | 8016.30 | 0.00 | 0.62 | -3987.12 |
| El + El^2^ + TRI + TRI^2^ + TPI + AllR_DEN_ + CC + W_D_ + VH + N + Edge_D_ + Fire | 17 | 8017.27 | 0.97 | 0.38 | -3991.61 |
| El + El^2^ + TRI + TRI^2^ + TPI + AllR_DEN_ + CC + Veg + RT­_D_ + N + Edge_D_ + Fire + CC*Fire | 24 | 8031.60 | 15.30 | 0.00 | -3991.76 |
| El + El^2^ + TRI + TRI^2^ + TPI + AllR_DEN_ + Veg + RT­_D_ + N + Edge_D_ + Fire | 19 | 8037.23 | 20.93 | 0.00 | -3999.59 |
| El + TRI + TRI^2^ + TPI + AllR_DEN_ + CC + W_D_ + VH + N + Edge_D_ + Fire + CC*Fire | 20 | 8039.85 | 23.55 | 0.00 | -3999.90 |
| **Fall: Night** | | | | | |
| El + El^2^ + TRI + TRI^2^ + TPI + AllR_DEN_ + CC + W_D_ + VH + N + Edge_D_ + Fire + CC*Fire | 21 | 19191.40 | 0.00 | 1.00 | -9574.69 |
| El + El^2^ + TRI + TRI^2^ + TPI + AllR_DEN_ + CC + W_D_ + VH + N + Edge_D_ + Fire | 17 | 19203.61 | 12.21 | 0.00 | -9584.79 |
| El + El^2^ + TRI + TRI^2^ + TPI + AllR_DEN_ + CC + Veg + RT­_D_ + N + Edge_D_ + Fire + CC*Fire | 24 | 19241.04 | 49.64 | 0.00 | -9596.50 |
| El + TRI + TRI^2^ + TPI + AllR_DEN_ + CC + W_D_ + VH + N + Edge_D_ + Fire + CC*Fire | 20 | 19246.57 | 55.17 | 0.00 | -9603.27 |
| El + El^2^ + TRI + TRI^2^ + TPI + AllR_DEN_ + Veg + RT­_D_ + N + Edge_D_ + Fire | 19 | 19253.80 | 62.40 | 0.00 | -9607.89 |
| **Winter: Day** | | | | | |
| El + El^2^ + TRI + TRI^2^ + TPI + AllR_DEN_ + CC + Veg + RT­_D_ + N + Edge_D_ + Fire + CC*Fire | 24 | 7540.01 | 0.00 | 0.69 | -3745.96 |
| El + El^2^ + TRI + TRI^2^ + TPI + AllR_DEN_ + Veg + RT­_D_ + N + Edge_D_ + Fire | 19 | 7541.60 | 1.59 | 0.31 | -3751.77 |
| El + El^2^ + TRI + TRI^2^ + AllR_DEN_ + W_D_ + N + Veg + RT_D_ + Fire | 18 | 7559.75 | 19.74 | 0.00 | -3761.85 |
| El + El^2^ + TRI + TRI^2^ + TPI + AllR_DEN_ + CC + W_D_ + VH + N + Edge_D_ + Fire + CC*Fire | 21 | 7606.79 | 66.78 | 0.00 | -3782.36 |
| El + El^2^ + TRI + TRI^2^ + TPI + AllR_DEN_ + CC + W_D_ + VH + N + Edge_D_ + Fire | 17 | 7610.17 | 70.17 | 0.00 | -3788.06 |
| **Winter: Night** | | | | | |
| El + El^2^ + TRI + TRI^2^ + TPI + AllR_DEN_ + CC + Veg + RT­_D_ + N + Edge_D_ + Fire + CC*Fire | 24 | 24463.22 | 0.00 | 0.96 | -12207.60 |
| El + El^2^ + TRI + TRI^2^ + TPI + AllR_DEN_ + Veg + RT­_D_ + N + Edge_D_ + Fire | 19 | 24469.78 | 6.57 | 0.04 | -12215.88 |
| El + El^2^ + TRI + TRI^2^ + AllR_DEN_ + W_D_ + N + Veg + RT_D_ + Fire | 18 | 24568.96 | 105.74 | 0.00 | -12266.47 |
| El + El^2^ + TRI + TRI^2^ + TPI + AllR_DEN_ + CC + W_D_ + VH + N + Edge_D_ + Fire + CC*Fire | 21 | 24636.58 | 173.37 | 0.00 | -12297.28 |
| El + El^2^ + TRI + TRI^2^ + TPI + AllR_DEN_ + CC + W_D_ + VH + N + Edge_D_ + Fire | 17 | 24643.23 | 180.02 | 0.00 | -12304.61 |
| **Spring: Day** | | | | | |
| El + TRI + TRI^2^ + TPI + AllR_DEN_ + CC + W_D_ + VH + N + Edge_D_ + Fire + CC*Fire | 20 | 14358.38 | 0.00 | 1.00 | -7159.17 |
| El + TRI + TRI^2^ + TPI + AllR_DEN_ + CC + W_D_ + VH + N + Edge_D_ + Fire | 16 | 14373.55 | 15.16 | 0.00 | -7170.76 |
| El + El^2^ + TRI + TRI^2^ + TPI + AllR_DEN_ + CC + W_D_ + VH + N + Edge_D_ + Fire | 17 | 14375.34 | 16.96 | 0.00 | -7170.66 |
| El + TRI + TRI^2^ + TPI + AllR_DEN_ + CC + W_D_ + VH + N + Edge_D_ + Fire + AllR_DEN_*VH | 17 | 14375.55 | 17.17 | 0.00 | -7170.76 |
| El + El^2^ + TRI + TRI^2^ + TPI + CC + W_D_ + VH + Edge_D_ + Fire + CC*Fire | 19 | 14382.57 | 24.19 | 0.00 | -7172.27 |
| **Spring: Night** | | | | | |
| El + TRI + TRI^2^ + TPI + AllR_DEN_ + CC + W_D_ + VH + N + Edge_D_ + Fire + CC*Fire | 20 | 34039.20 | 0.00 | 0.72 | -16999.59 |
| El + TRI + TRI^2^ + TPI + AllR_DEN_ + CC + W_D_ + VH + N + Edge_D_ + Fire | 16 | 34043.35 | 4.15 | 0.18 | -17005.67 |
| El + TRI + TRI^2^ + TPI + AllR_DEN_ + CC + W_D_ + VH + N + Edge_D_ + Fire + AllR_DEN_*VH | 17 | 34044.23 | 5.03 | 0.06 | -17005.11 |
| El + El^2^ + TRI + TRI^2^ + TPI + AllR_DEN_ + CC + W_D_ + VH + N + Edge_D_ + Fire | 17 | 34044.92 | 5.71 | 0.04 | -17005.45 |
| El + El^2^ + TRI + TRI^2^ + TPI + CC + W_D_ + VH + Edge_D_ + Fire + CC*Fire | 19 | 34069.32 | 30.12 | 0.00 | -17015.65 |
| **Monsoon: Day** | | | | | |
| El + El^2^ + TRI + TRI^2^ + TPI + CC + VH + N + Edge_D_ + Fire + CC*Fire | 19 | 15561.71 | 0.00 | 0.98 | -7761.84 |
| El + TRI + TRI^2^ + TPI + AllR_DEN_ + CC + W_D_ + VH + N + Edge_D_ + Fire + CC*Fire | 20 | 15571.51 | 9.79 | 0.01 | -7765.74 |
| El + El^2^ + TRI + TRI^2^ + TPI + AllR_DEN_ + CC + W_D_ + VH + N + Edge_D_ + Fire | 17 | 15572.66 | 10.94 | 0.00 | -7769.32 |
| El + TRI + TRI^2^ + TPI + AllR_DEN_ + CC + W_D_ + VH + N + Edge_D_ + Fire + AllR_DEN_*VH | 17 | 15573.70 | 11.98 | 0.00 | -7769.84 |
| El + El^2^ + TRI + TRI^2^ + TPI + CC + VH + N + Edge_D_ + Fire | 15 | 15574.25 | 12.53 | 0.00 | -7772.12 |
| **Monsoon: Night** | | | | | |
| El + El^2^ + TRI + TRI^2^ + TPI + CC + VH + N + Edge_D_ + Fire + CC*Fire | 19 | 34693.50 | 0.00 | 1.00 | -17327.74 |
| El + El^2^ + TRI + TRI^2^ + TPI + CC + VH + N + Edge_D_ + Fire | 15 | 34709.58 | 16.08 | 0.00 | -17339.79 |
| El + El^2^ + TRI + TRI^2^ + TPI + AllR_DEN_ + CC + W_D_ + VH + N + Edge_D_ + Fire | 17 | 34713.22 | 19.72 | 0.00 | -17339.61 |
| El + TRI + TRI^2^ + TPI + AllR_DEN_ + CC + W_D_ + VH + N + Edge_D_ + Fire + CC*Fire | 20 | 34718.25 | 24.75 | 0.00 | -17339.12 |
| El + TRI + TRI^2^ + TPI + AllR_DEN_ + CC + W_D_ + VH + N + Edge_D_ + Fire + AllR_DEN_*VH | 17 | 34729.88 | 36.38 | 0.00 | -17347.93 |

Model terms: El = elevation; TRI = topographic ruggedness index; TPI = topographic position index; AllR_DEN_ = density of all road classes; CC = percent canopy cover; W_D_ = distance to natural and artificial water bodies; VH = classified vegetation height (reference = 0-1 m); N = northness index; Edge_D_ = distance to forest edge; Fire = fire history class (reference = unburned in over 17 years); Veg = dominant vegetation type (reference = grassland); RT_D_ = distance to recreation sites and trails.

*Figures*


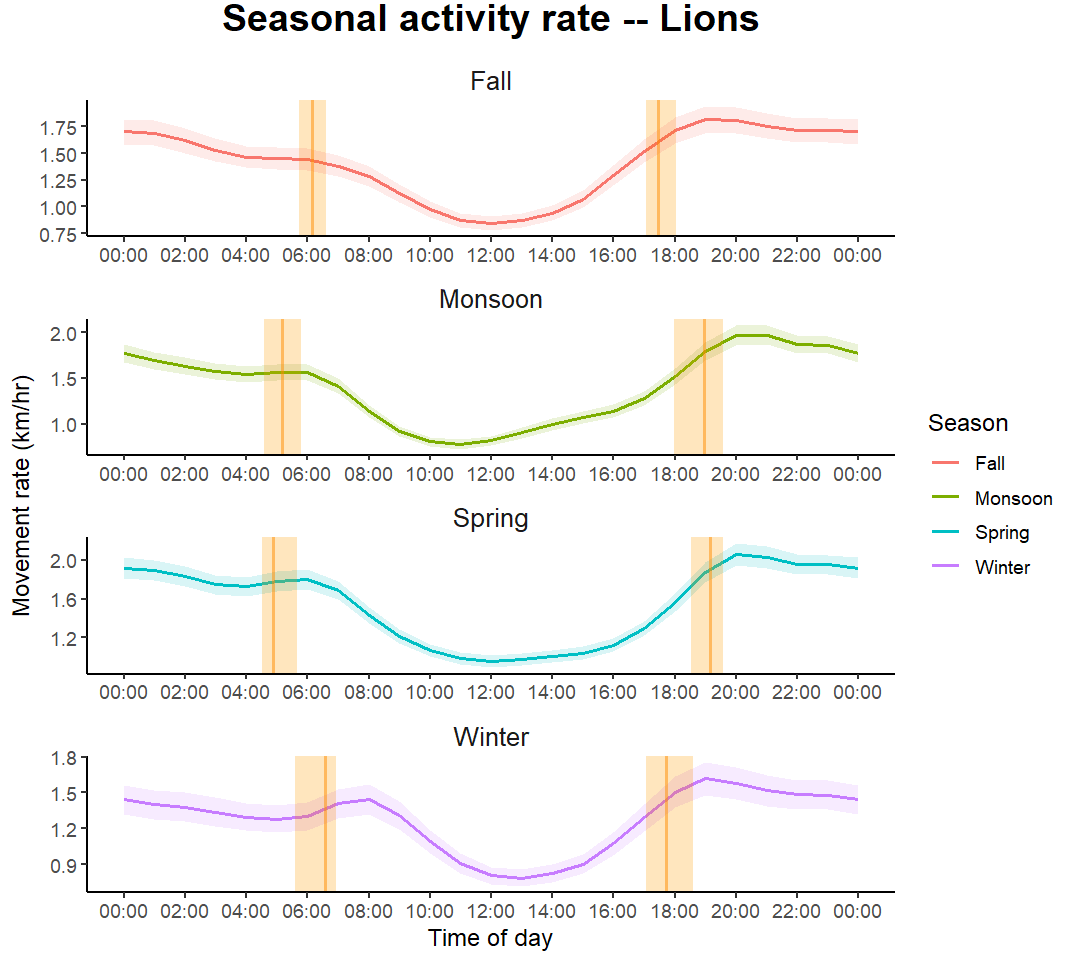


Figure C1. Seasonal activity rates (km/hr) for collared mountain lions in west-central New Mexico between 2021 and 2023 modeled with generalized additive mixed models. Vertical bars represent civil twilight with the darker line showing the average for that season.


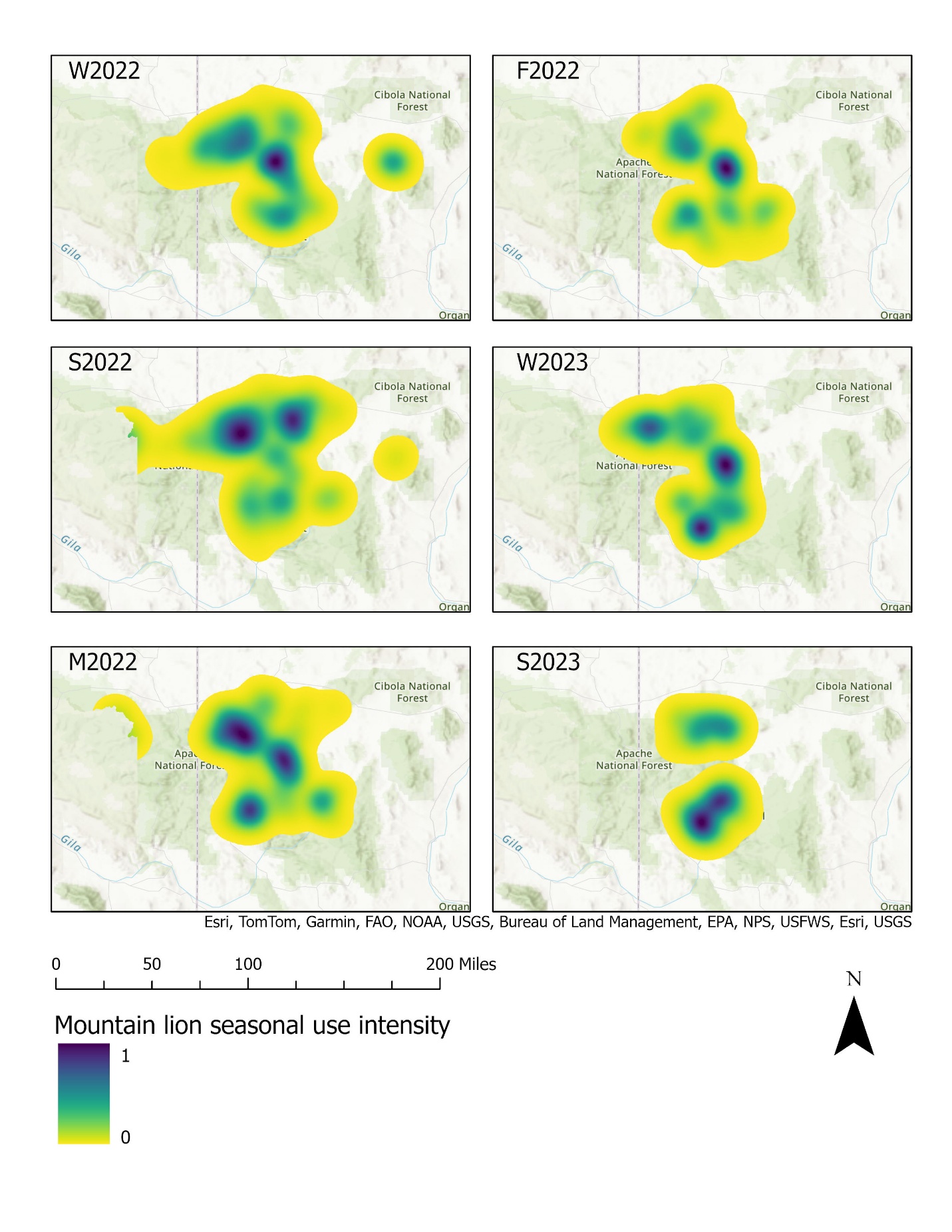


Figure C2. Mountain lion population UDs partitioned seasonally into winter (W), spring (S), monsoon M), and fall (F) between 2022-2023 in east-central Arizona and west-central New Mexico with dark blue representing higher intensities of use. Data from tribal lands are excluded from figures based on an agreement between local tribes and USFWS.


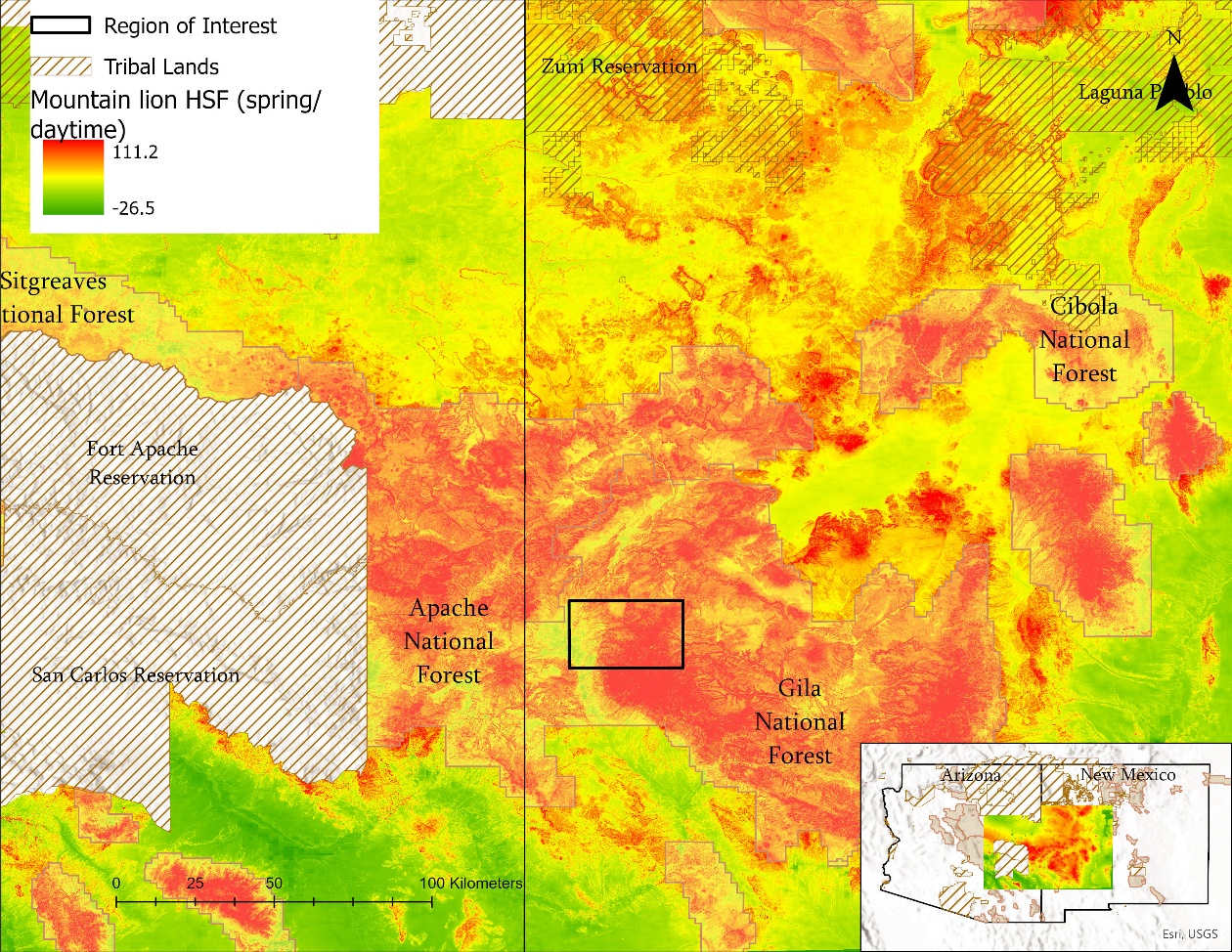

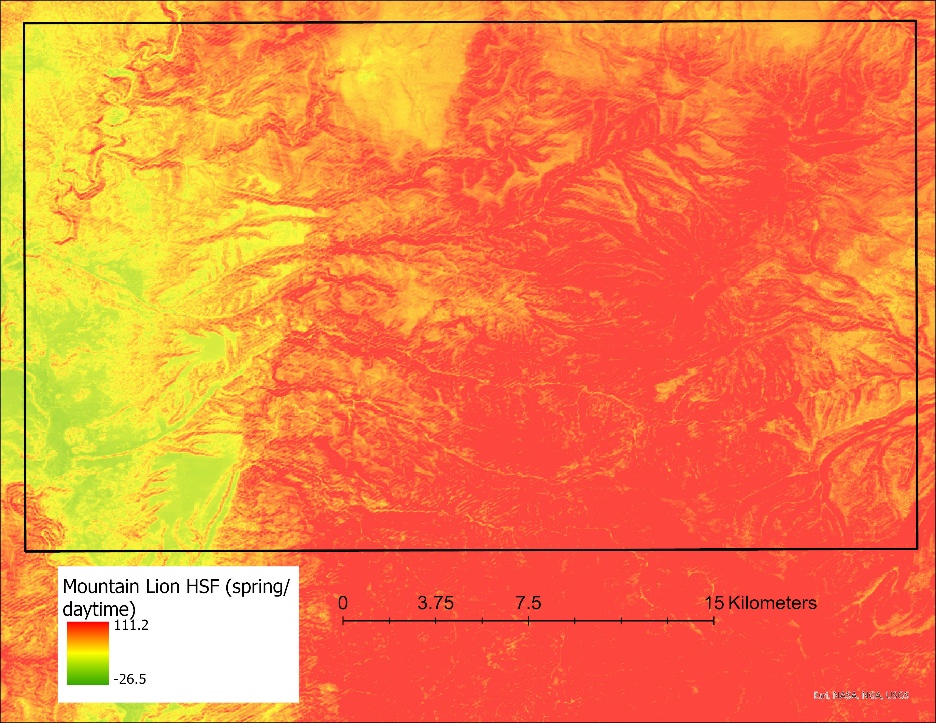


Figure C3. Mountain lion spring/daytime habitat selection function (HSF) for 2021-2023 in east-central Arizona and west-central New Mexico with red indicating higher probabilities of selection and green indicating lower probabilities of selection. The region of interest shows a portion of the study area in the Gila National Forest, NM. Data from tribal lands are excluded from figures based on an agreement between local tribes and USFWS.


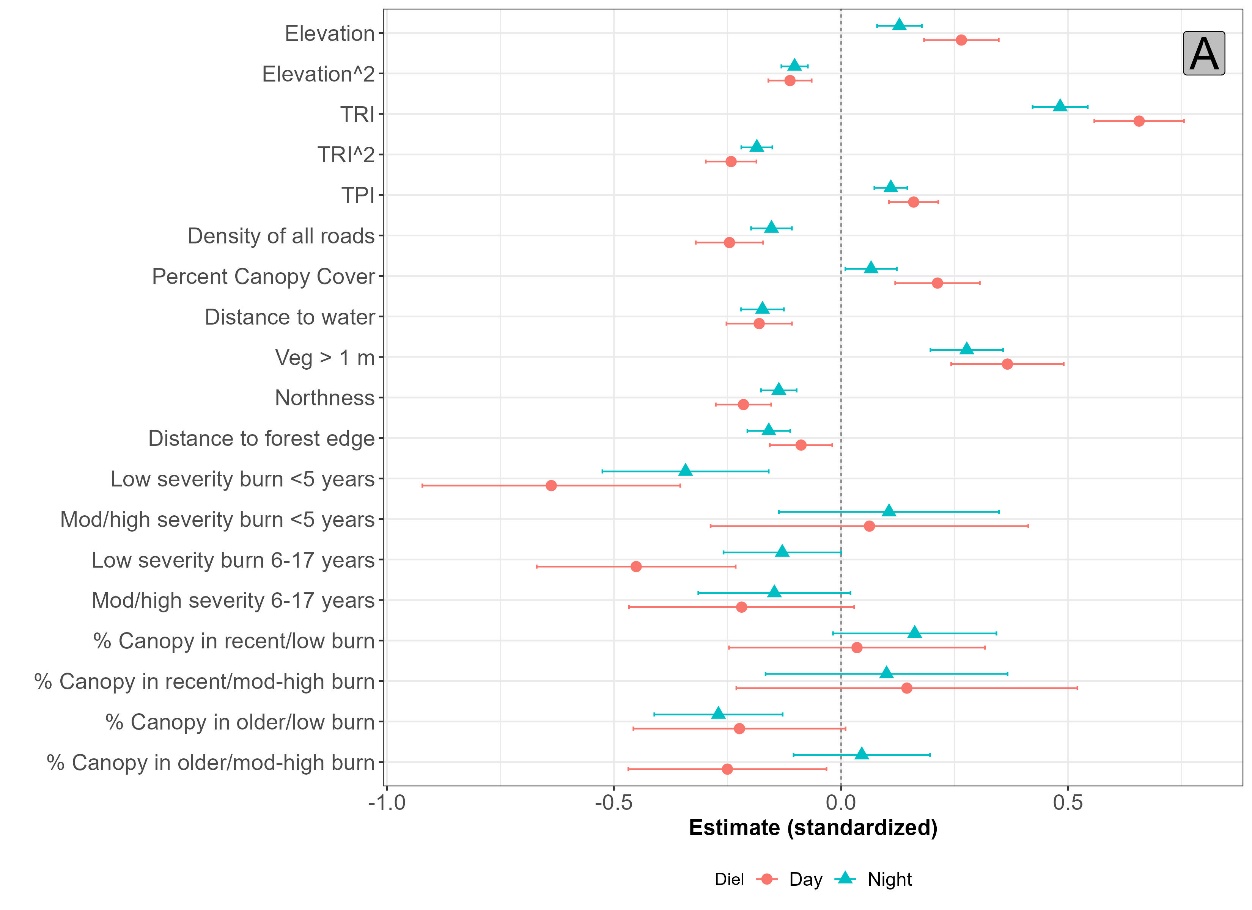

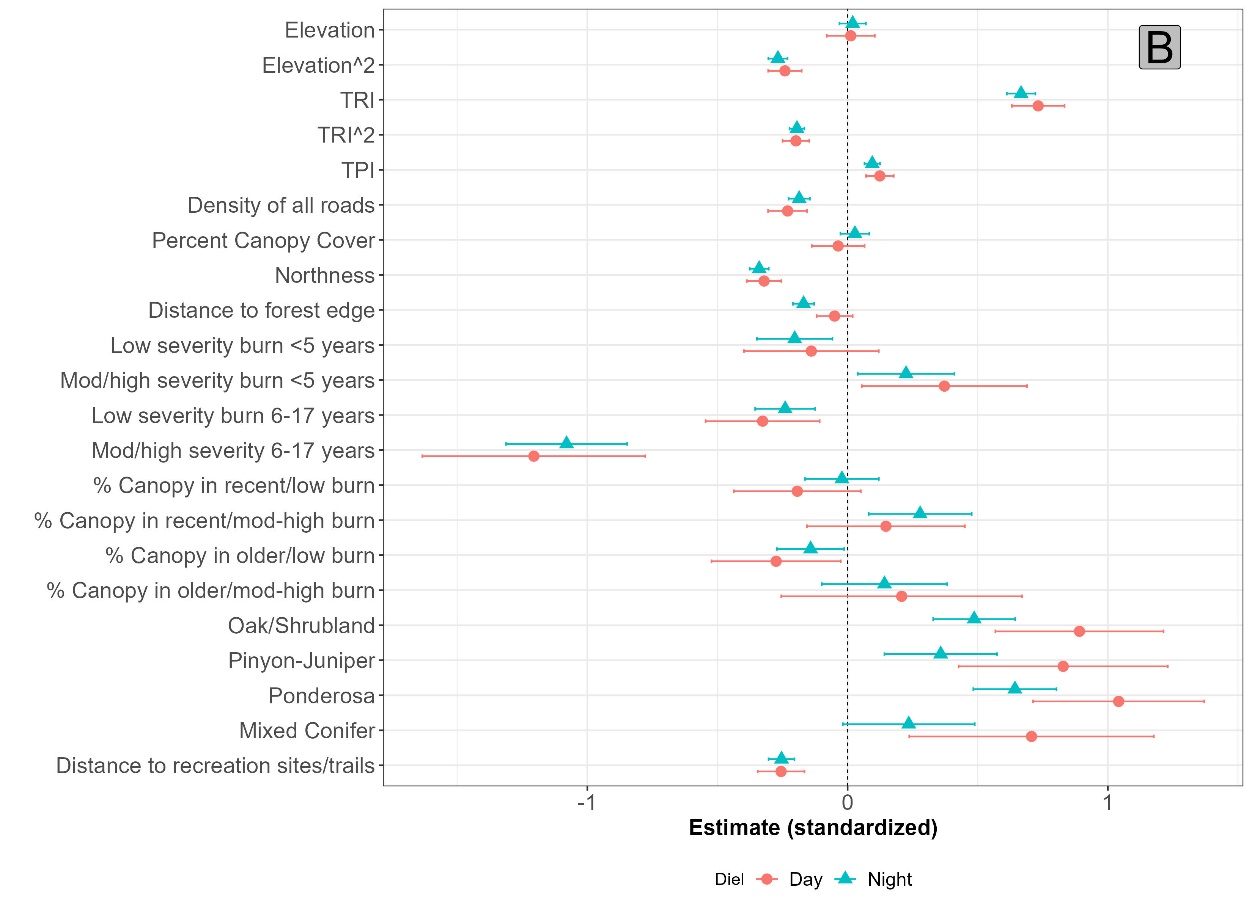


Figure C4: Standardized regression coefficients for Fall (A) and Winter (B) with 95% confidence intervals for the top mountain lion HSF models in west-central NM between 2021-2023, colored by diel period. For vegetation height the reference category was vegetation < 1 m; for dominant vegetation type the reference category was grassland and pastures; for fire history the reference category was unburned within the past 17 years.


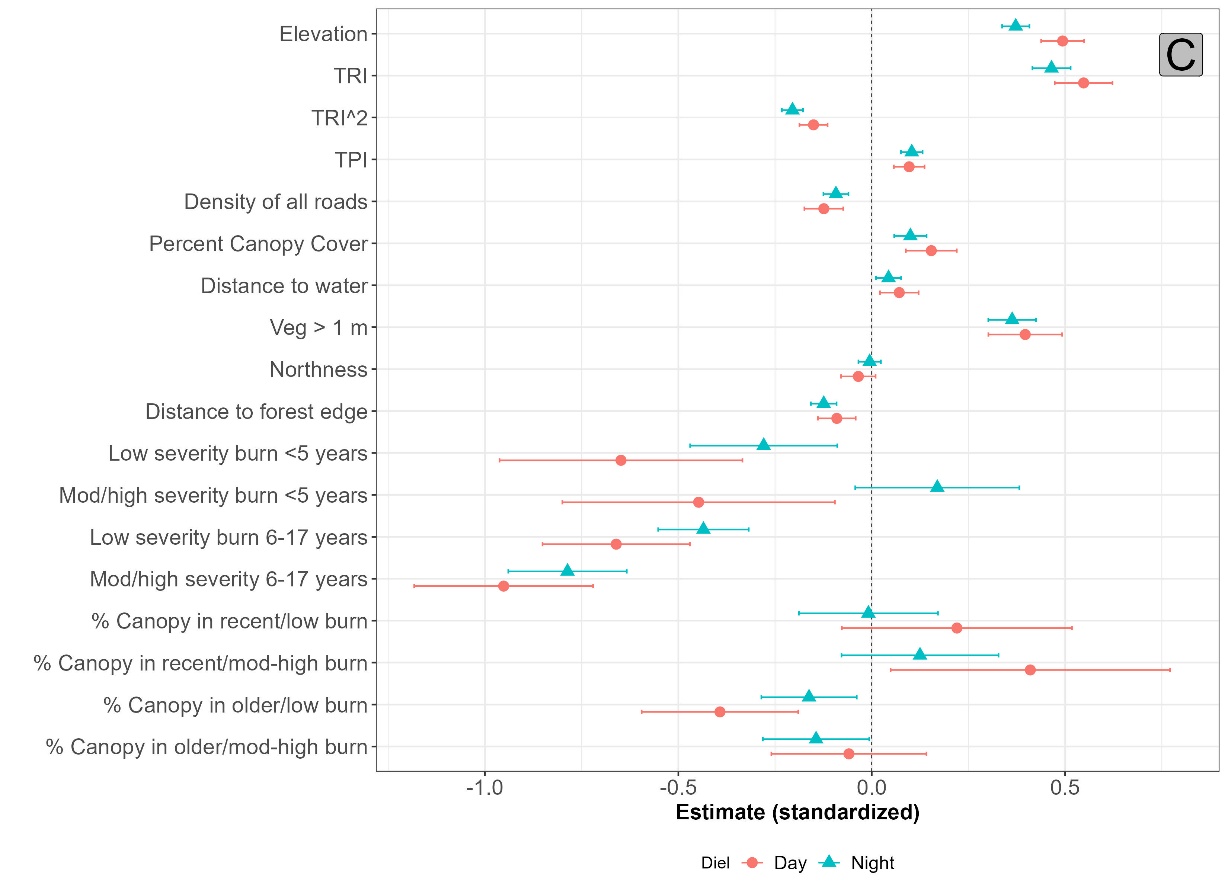

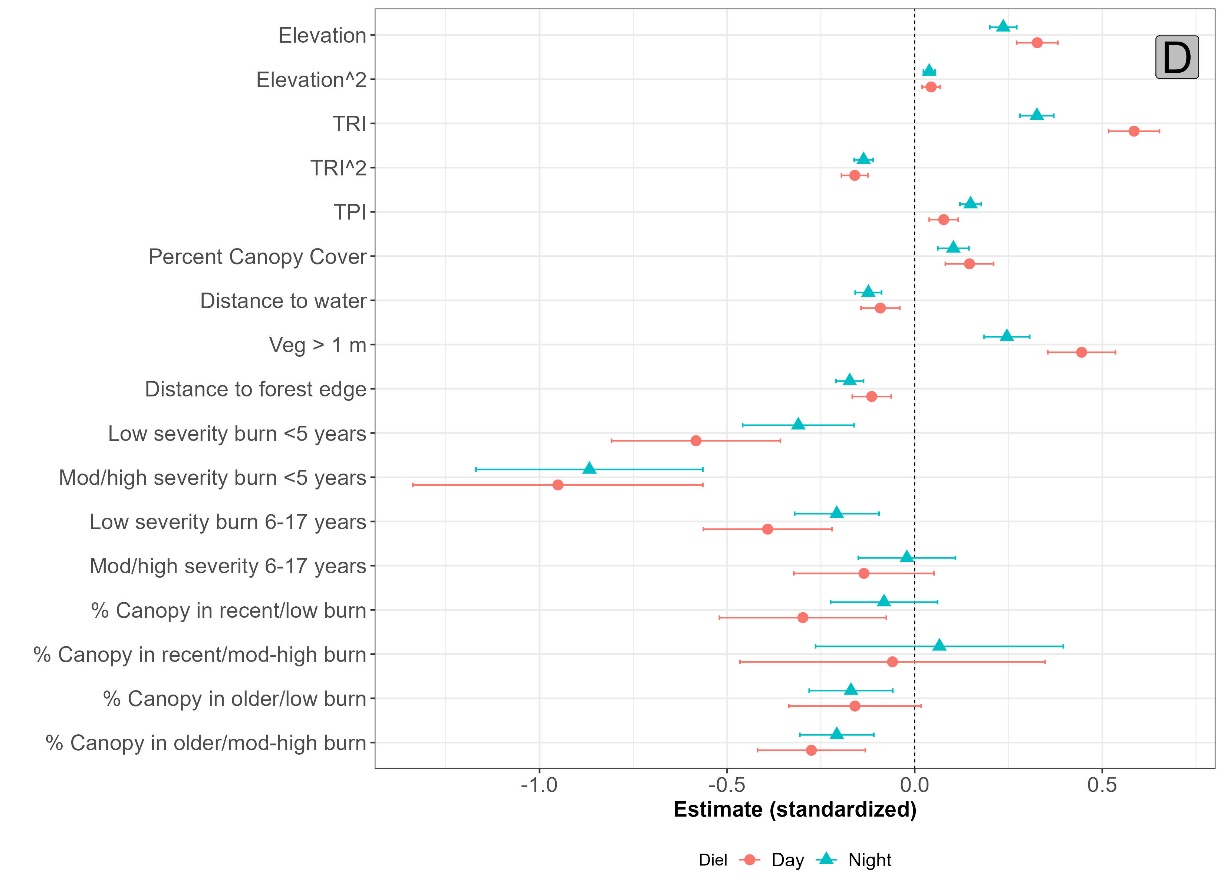


Figure C4 (*cont.*): Standardized regression coefficients for Spring (C) and Monsoon (D) with 95% confidence intervals for the top mountain lion HSF models in west-central NM between 2021-2023, colored by diel period. For vegetation height the reference category was vegetation < 1 m; for dominant vegetation type the reference category was grassland and pastures; for fire history the reference category was unburned within the past 17 years.

SUPPLEMENTAL MATERIAL 4

MEXICAN WOLF RISKY PLACES INDEX

*Methods*

Following methods developed by Thompson (2022) for the creation of a Mexican gray wolf risky places index based on locations where elk were killed by Mexican wolves, we incorporated additional data collected from June 2021 to June 2023 to create an updated wolf risky places model. We conducted cluster investigations on collared Mexican wolves year-round, using data obtained from the Mexican gray wolf Interagency Field Team with collars set to a 2-hour fix rate in winter, fall, and monsoon season. We switched to a 1-hour fix rate during calving season to increase the likelihood of identifying kills of ungulate neonates. We selected collared animals to be followed based on access to their home range and previous monitoring history, with the goal of following a given pack for four months at a time and avoiding packs that primarily occupied wilderness areas or private lands. We defined clusters as at least two GPS locations within 100 m and 24 hours of each other and identified clusters, as well as randomly selected single locations during calving season, using modified code from Kindschuh et al. (2016). At clusters, we determined whether a carcass was present, its species, sex, age, and searched within a 50 m radius for evidence of a kill by wolves including spattered blood, hemorrhaging on the lower legs and hindquarters, a scattered carcass and/or rumen, chewing on bones, evidence of a chase, or the presence of wolf scat or tracks (Alt and Eckert 2017). We excluded any clusters with carcasses that we deemed scavenging by Mexican wolves rather than kills. In addition to elk carcasses identified at Mexican wolf location clusters, we also included the locations of collared adult and neonatal elk that we found to be killed by wolves based on mortality investigations. Whenever possible, we performed necropsies on the same day the mortality signal was received and used the same criteria as cluster investigations to determine causes of mortality.

*Analysis*

We combined locations of wolf-killed elk collected by Thompson (2022), Martinez (2024) and Smith et al. (2023) with subsequently collected wolf-kill locations to create models of Mexican wolf risky places that incorporated data from 2015-2023 (Figure D1). To remain consistent with collared elk mortalities, which only included females and calves, we excluded kills of adult (> 2 years) and sub-adult (1-2 years) male elk (*n* = 34) and ensured that no duplicates occurred in these data from a collared elk being found at a Mexican wolf cluster. We assigned kills found at Mexican wolf clusters to the pack that created that cluster and assigned collared elk that were killed by Mexican wolves to the home range of the pack that overlapped the carcass location at that time. To avoid model estimation issues resulting from a single observation associated with a pack, we assigned the kills of any pack with only one kill to another pack that had a home range that overlapped the kill location and had multiple associated kills. As a result of the inequality in the number of kill locations assigned to each pack, sampling available points randomly across all Mexican wolf homes ranges may have produced bias for areas that were not truly available to Mexican wolves for hunting elk, or against areas that were not monitored sufficiently to produce comparable kill site data. We therefore sampled random points from each pack’s annual home range at a ratio of 20 available locations for each kill location assigned to that pack in each year. We buffered available and kill site locations by 150 m to account for the hunting process (Thompson 2022) and removed available locations that overlapped kill sites or other available locations, as this was the buffer size we subsequently used to extract habitat covariates. These covariates included: elevation, slope, northness, Vector Ruggedness Measure, Topographic Ruggedness Index, dominant vegetation type, vegetation cover, vegetation height, distance to forest cover, canopy cover, number of open canopy pixels in a 500 m rolling window, distance to water bodies, human density, distance to recreation sites or trails, distance to roads of three types, densities of roads of three types, burn severity, and years since last fire, and combined the burn severity and age classes to create a single metric of fire history (see Supplementary Material 1 – Table A2 and Supplementary Material 3 for complete descriptions of covariate manipulations).

Prior to modeling, we tested all linear covariates for multicollinearity using a Spearman’s rank correlation test and if any variable combinations produced a Spearman’s rho greater than 0.6, we did not include them in the same model. We centered and scaled all covariates and resampled the available locations for each pack to a ratio of 10 available locations per kill. We did not subset the data seasonally due to small sample sizes. We then constructed mixed-effects logistic regression models in the program R using the package *lme4*, including a random effect for pack ID (Table D1; Bates et al. 2015). We selected top models based on their Akaike’s Information Criterion for small sample sizes (AIC_C_) score and assessed model performance using a 10-fold cross validation repeated 100 times with 10 bins (Boyce et al. 2002) and checked variance inflation factor (VIF) scores for the top models.

*Results*

The final data included 557 locations where elk were killed by Mexican wolves and 5,570 available locations, with 133 adult female elk kills, 173 female yearlings, and 289 neonates. Kills were assigned to 32 wolf packs and the number of kills assigned to each pack ranged from 2 to 83. The top model for Mexican gray wolf risky places included canopy openness (480 m), the distance to the nearest recreation site or trail, the distance to the nearest road (all types), dominant vegetation type, slope, and burn history (Table D2; Figure D2). While three additional models were within ΔAIC_C_ < 2 of the top model, these models contained the top model structure with the addition of uninformative parameters, and were removed from consideration. The 10-fold cross validation resulted in a Spearman’s rho of 0.988 and all VIF scores for predictors were < 1.7. The odds of a location being a site where an elk was killed by Mexican wolves were higher in more open areas (less than 30% canopy cover), closer to recreation sites or trails, on flatter slopes, and in areas that burned within 17 years (Figure D3). Compared to grassland, locations in wet meadows/pastures were more likely to be a kill while locations in oak/shrubland, ponderosa, and mixed conifer/aspen habitats were less likely to be a kill. The effect of piñon-juniper habitat was not significantly different from grassland (α = 0.05), and the effect of distance to road was only weakly significant (p = 0.056), with the odds of a location being a kill site increasing closer to roads (Figure D3).

*Literature cited*

Alt, K., and M. Eckert. 2017. Predation ID manual: predator kill and scavenging characteristics. Simon and Schuster.

Bates, D., M. Maechler, B. Bolker, and S. Walker. 2015. Fitting linear mixed-effects models using lme4. Journal of Statistical Software 67:1–48.

Boyce, M. S., P. R. Vernier, S. E. Nielsen, and F. K. A. Schmiegelow. 2002. Evaluating resource selection functions. Ecological Modelling 157:281–300.

Kindschuh, S. R., J. W. Cain, D. Daniel, and M. A. Peyton. 2016. Efficacy of GPS cluster analysis for predicting carnivory sites of a wide‐ranging omnivore: the American black bear. Ecosphere 7:e01513.

Martinez, S. I. 2024. Kill rates and prey composition of Mexican gray wolves (Canis lupus baileyi) and cougars (Puma concolor) in the Southwest. M.S Thesis. New Mexico State University, Las Cruces.

Smith, J. B., A. R. Greenleaf, and J. K. Oakleaf. 2023. Kill rates on native ungulates by Mexican gray wolves in Arizona and New Mexico. Journal of Wildlife Management 87:e22491.

Thompson, C. 2022. Elk habitat selection in response to predation risk from Mexican gray wolves. MS Thesis, New Mexico State University, Las Cruces, USA.

Vore, J. M., and E. M. Schmidt. 2001. Movements of female elk during calving season in Northwest Montana. Wildlife Society Bulletin 29:720–725.

Wolfe, L. L., M. C. Fisher, T. R. Davis, and M. W. Miller. 2014. Efficacy of a low-dosage combination of Butorphanol, Azaperone, and Medetomidine (BAM) to immobilize rocky mountain elk. Journal of Wildlife Diseases 50:676–680.

*Tables*

Table D1. Model structures tested for the Mexican wolf risky places index using locations where elk were killed by Mexican wolves in east-central Arizona and west-central New Mexico between 2015 and 2023. All models included a random effect for unique pack ID (*n* = 32).

| Model Structure | |
| --- | --- |
| 1 | Burn + W_D_ + El + R2_DEN_ + Open_500_ + Priv_D_ + R3_D_ |
| 2 | Burn + W_D_ + El + R2_DEN_ + Open_500_ + Priv_D_ + TRI |
| 3 | Burn + W_D_ + El + R2_DEN_ + CC + Priv_D_ + R3_D_ |
| 4 | Burn + W_D_ + El + R2_DEN_ + HC + Priv_D_ + R3_D_ |
| 5 | Burn + Sl + El + R2_DEN_ + Open_500_ + Priv_D_ + R3_D_ |
| 6 | Burn + W_D_ + R2_DEN_ + Open_500_ + Priv_D_ + R3_D_ + Veg |
| 7 | Burn + W_D_ + R2_DEN_ + Open_500_ + Priv_D_ + TRI + Veg |
| 8 | Burn + W_D_ + R2_DEN_ + CC + Priv_D_ + R3_D_ + Veg |
| 9 | Burn + W_D_ + R2_DEN_ + HC + Priv_D_ + R3_D_ + Veg |
| 10 | Burn + Sl + R2_DEN_ + Open_500_ + Priv_D_ + R3_D_ + Veg |
| 11 | Burn + W_D_ + El + R2_DEN_ + Open_500_ + Priv_D_ + Priv_D_^2^ + R3_D_ |
| 12 | Burn + TRI + R2_DEN_ + Open_500_ + Priv_D_ + R3_D_ + Veg |
| 13 | Burn + VRM + VRM^2^ + R2_DEN_ + Open_500_ + Priv_D_ + R3_D_ + Veg |
| 14 | VRM + VRM^2^ + R2_DEN_ + Open_500_ + Priv_D_ + R3_D_ + Veg + YSF |
| 15 | Fire + R2_DEN_ + Open_500_ + Priv_D_ + R3_D_ + Veg |
| 16 | YSF + R2_DEN_ + Open_500_ + Priv_D_ + R3_D_ + Veg |
| 17 | Fire + R2_DEN_ + Open_500_ + Priv_D_ + R3_D_ + Veg + W_D_ |
| 18 | Fire + R2_DEN_ + Open_500_ + Priv_D_ + R3_D_ + El + W_D_ |
| 19 | YSF + R2_DEN_ + Open_500_ + Priv_D_ + R3_D_ + Veg + W_D_ |
| 20 | Burn + R2_DEN_ + Open_500_ + Priv_D_ + R3_D_ + Veg + Sl |
| 21 | VRM + VRM^2^ + W_D_ + R2_DEN_ + Open_500_ + Priv_D_ + R3_D_ + Veg + Burn |
| 22 | Burn + Sl + R2_DEN_ + Open_500_ + Priv_D_ + R3_D_ + El |
| 23 | Burn + W_D_ + R2_DEN_ + Open_500_ + Priv_D_ + Veg + Sl |
| 24 | YSF + R3_D_ + R2_DEN_ + Open_500_ + Priv_D_ + Veg + Sl |
| 25 | TRI + R2_DEN_ + Open_500_ + Priv_D_ + Veg + Fire |
| 26 | R2_DEN_ + Sl + Priv_D_ + Burn + Veg + HC + Edge_D_ + W_D_ + R3_D_ |
| 27 | R2_DEN_ + Open_500_ + Priv_D_ + Burn + Veg + R1_DEN_ + R3_D_ |
| 28 | R2_DEN_ + Priv_D_ + Burn + Veg + R1_DEN_ + R3_D_ |
| 29 | R2_DEN_ + Priv_D_ + Burn + Veg + R3_D_ |
| 30 | R2_DEN_ + Priv_D_ + Fire + Veg + R3_D_ |
| 31 | AllR_DEN_ + Open_500_ + Priv_D_ + Veg + R3_D_ |
| 32 | Priv_D_ + AllR_DEN_ + Edge_D_ + VRM + VRM^2^ + HC + Veg + Burn |
| 33 | R2_DEN_ + TRI + R3_D_ + Edge_D_ + Priv_D_ + HC + Veg |
| 34 | R2_DEN_ + Priv_D_ + Burn + Veg + R1_DEN_ + R3_D_ + TRI |
| 35 | RT_D_ + R2_DEN_ + W_D_ + R3_D_ + Edge_D_ + Priv_D_ + VRM + VRM^2^ + HC + Veg + Burn |
| 36 | Open_500_ + RT_D_ + AllR_DEN_ + W_D_ + Edge_D_ + Priv_D_ + VRM + VRM^2^ + Veg + YSF |
| 37 | HC + AllR_DEN_ + W_D_ + Edge_D_ + Priv_D_ + VRM + VRM^2^ + Veg + Burn |
| 38 | Burn + R2_DEN_ + Open_500_ + Edge_D_ + Priv_D_ + Veg + R1_DEN_ |
| 39 | Burn + AllR_DEN_ + Open_500_ + Priv_D_ + Veg + Edge_D_ + Sl |
| 40 | Burn + R2_DEN_ + HC + Priv_D_ + Veg + Edge_D_ + TRI + R3_D_ |
| 41 | Burn + AllR_DEN_ + HC + Priv_D_ + N + Veg + Edge_D_ |
| 42 | Burn + R2_DEN_ + Open_500_ + Priv_D_ + Priv_D_^2^ + Veg + R3_D_ |
| 43 | YSF + R2_DEN_ + Open_500_ + Priv_D_ + Priv_D_^2^ + Veg + R3_D_ |
| 44 | Burn + R2_DEN_ + Priv_D_ + Priv_D_^2^ + Veg + R3_D_ + HC |
| 45 | Burn + R2_DEN_ + Open_500_ + Priv_D_ + Priv_D_^2^ + Veg + R3_D_ + Sl |
| 46 | YSF + R2_DEN_ + Open_500_ + Priv_D_ + Priv_D_^2^ + Veg + R3_D_ + Sl |
| 47 | Burn + R2_DEN_ + Open_500_ + Priv_D_ + Priv_D_^2^ + Veg + R3_D_ + N |
| 48 | Burn + R2_DEN_ + Open_500_ + Priv_D_ + Priv_D_^2^ + Veg + R3_D_ + Sl |
| 49 | Burn + R2_DEN_ + HC + Priv_D_ + Priv_D_^2^ + Veg + R3_D_ + Sl + N |
| 50 | Burn + R2_DEN_ + Priv_D_ + Priv_D_^2^ + Veg + R3_D_ + HC + N |
| 51 | Burn + R2_DEN_ + Priv_D_ + Priv_D_^2^ + Veg + R3_D_ + HC + Sl |
| 52 | R2_DEN_ + Open_500_ + Priv_D_ + Priv_D_^2^ + Veg + R3_D_ + Sl + Fire |
| 53 | Burn + Open_500_ + W_D_ + Veg + R3_D_ + Sl + N |
| 54 | Burn + HC + W_D_ + Veg + R3_D_ + Sl + N |
| 55 | Open_500_ + W_D_ + Veg + R3_D_ + Sl + N |
| 56 | HC + W_D_ + Veg + R3_D_ + Sl + N |
| 57 | Burn + Open_500_ + W_D_ + Veg + R3_D_ + VRM + VRM^2^ + N |
| 58 | Burn + HC + W_D_ + Veg + R3_D_ + VRM + VRM^2^ + N |
| 59 | Fire + Open_500_ + W_D_ + Veg + R3_D_ + Sl + N |
| 60 | Burn + Open_500_ + W_D_ + Veg + R3_D_ + Sl + N + R2_DEN_ |
| 61 | Burn + Open_500_ + W_D_ + Veg + R3_D_ + TRI + N |
| 62 | Burn + Open_500_ + W_D_ + Veg + R3_D_ + Sl + N + Priv_D_ |
| 63 | Burn + Open_500_ + W_D_ + Veg + R3_D_ + Sl + N + Edge_D_ |
| 64 | Burn + R2_DEN_ + Open_500_ + Priv_D_ + Priv_D_^2^ + Veg + R3_D_ + Sl + N + W_D_ |
| 65 | Burn + R2_DEN_ + Open_500_ + Priv_D_ + Veg + R3_D_ + Sl + N + W_D_ |
| 66 | Burn + R2_DEN_ + Open_500_ + Priv_D_ + Veg + Sl + N + W_D_ |
| 67 | Open_500_ + W_D_ + Veg + R3_D_ + Sl + N + Priv_D_ + YSF |
| 68 | Burn + Open_500_ + W_D_ + Veg + R3_D_ + Sl + N + Priv_D_ + Edge_D_ |
| 69 | Burn + Open_500_ + W_D_ + Veg + R3_D_ + Sl + N + Priv_D_ + Priv_D_^2^ |
| 70 | Burn + Open_500_ + W_D_ + Veg + R3_D_ + Sl + N + Priv_D_ + Open_500_^2^ |
| 71 | Burn + Open_500_ + W_D_ + Veg + R3_D_ + Sl + N + Priv_D_ + R3_D_^2^ |
| 72 | Burn + Open_500_ + W_D_ + Veg + R3_D_ + Sl + N + Priv_D_ + R1_DEN_ |
| 73 | Burn + Open_500_ + W_D_ + Veg + R3_D_ + Sl + N + Edge_D_ + Priv_D_ + R1_DEN_ |
| 74 | Burn + Open_500_ + W_D_ + El + R3_D_ + Sl + N + Priv_D_ + Edge_D_ |
| 75 | Burn + Open_500_ + W_D_ + El + R3_D_ + Sl + N + Priv_D_ + El*Burn |
| 76 | Burn + Open_500_ + W_D_ + El + R3_D_ + Sl + N + Priv_D_ + El*YSF + YSF |
| 77 | Burn + Open_500_ + W_D_ + El + R3_D_ + Sl + N + Priv_D_ + El*Burn + Edge_D_ |
| 78 | Burn + W_D_ + Veg + R3_D_ + Sl + N + Priv_D_ + Priv_D_^2^ + R1_DEN_ |
| 79 | Burn + W_D_ + Veg + R3_D_ + Sl + N + R1_DEN_ |
| 80 | Burn + Open_500_ + W_D_ + El + R3_D_ + Sl + N + Priv_D_ + Priv_D_^2^ + El*Burn + Edge_D_ |
| 81 | Burn + Open_500_ + W_D_ + El + R3_D_ + Sl + N + El*Burn + Edge_D_ |
| 82 | Burn + Open_500_ + W_D_ + El + R3_D_ + Sl + N + Priv_D_ + El*Burn + Edge_D_ + R1_DEN_ |
| 83 | Burn + W_D_ + El + R3_D_ + Sl + N + Priv_D_ + Priv_D_^2^ + R1_DEN_ |
| 84 | Burn + W_D_ + Veg + R3_D_ + Sl + N + Priv_D_ + Priv_D_^2^ + R1_DEN_ + Edge_D_ |
| 85 | Burn + W_D_ + Veg + R3_D_ + Sl + N + Priv_D_ + Priv_D_^2^ + R2_DEN_ |
| 86 | Burn + W_D_ + Veg + R3_D_ + Sl + N + Priv_D_ + Priv_D_^2^ + AllR_DEN_ |
| 87 | Burn + W_D_ + Veg + El + El^2^ + R3_D_ + Sl + N + Priv_D_ + Priv_D_^2^ + R1_DEN_ |
| 88 | Burn + W_D_ + Veg + El + El^2^ + R3_D_ + Sl + N + Priv_D_ + Priv_D_^2^ + R1_DEN_ + R2_DEN_ |
| 89 | Burn + W_D_ + Veg + El + El^2^ + R3_D_ + Sl + N + Priv_D_ + R1_DEN_ + R2_DEN_ |
| 90 | Burn + W_D_ + Veg + R3_D_ + Sl + N + R1_DEN_ + R2_DEN_ |
| 91 | Burn + W_D_ + Veg + El + R3_D_ + Sl + N + Priv_D_ + Priv_D_^2^ + R1_DEN_ |
| 92 | Burn + W_D_ + Veg + R3_D_ + Sl + N + Priv_D_ + HC |
| 93 | Burn + Open_500_ + W_D_ + Veg + R3_D_ + Sl + N + Priv_D_ + Open_500_^2^ + R3_DEN_ |
| 94 | Burn + HC + W_D_ + Veg + R3­_D_ + Sl + N + R2_DEN_ |
| 95 | Burn + Open_500_ + W_D_ + Veg + R3_D_ + Sl + N + Priv_D_ + Open_500_^2^ + R1_DEN_ |
| 96 | Burn + Open_500_ + W_D_ + Veg + R3_D_ + Sl + N + Open_500_^2^ |
| 97 | Open_500_ + W_D_ + Veg + R3_D_ + Sl + Open_500_^2^ |
| 98 | Open_500_ + W_D_ + Veg + R3_D_ + Sl |
| 99 | Burn + W_D_ + Veg + R3_D_ + Sl + N + Priv_D_ + Priv_D_^2^ + R1_DEN_ + R2_DEN_ |
| 100 | Burn + W_D_ + El + El^2^ + R3_D_ + Sl + N + Priv_D_ + Priv_D_^2^ + R1_DEN_ + R2_DEN_ |
| 101 | Open_500_ + RT_D_ + AllR_DEN_ + W_D_ + Edge_D_ + Priv_D_ + VRM + VRM^2^ + Veg + N |
| 102 | Open_500_ + RT_D_ + AllR_DEN_ + W_D_ + Edge_D_ + Priv_D_ + Veg + N + Sl + Burn |
| 103 | Open_500_ + RT_D_ + AllR_DEN_ + W_D_ + Edge_D_ + Priv_D_ + Veg + N + Sl + Sl^2^ + Burn |
| 104 | Open_500_ + RT_D_ + W_D_ + Edge_D_ + Priv_D_ + Veg + N + Sl + Sl^2^ + YSF + AllR_D_ |
| 105 | Open_500_ + RT_D_ + Edge_D_ + Priv_D_ + Veg + N + Sl + Sl^2^ + YSF + AllR_D_ |
| 106 | CC + RT_D_ + W_D_ + Priv_D_ + Veg + N + Sl + Sl^2^ + Burn + AllR_D_ |
| 107 | RT_D_ + W_D_ + Priv_D_ + Veg + N + Sl + Burn + AllR_D_ |
| 108 | Open_500_ + RT_D_ + W_D_ + Priv_D_ + Veg + N + Sl + Burn + AllR_D_ |
| 109 | Open_500_ + RT_D_ + W_D_ + Veg + N + Sl + YSF + AllR_D_ |
| 110 | Open_500_ + RT_D_ + Priv_D_ + Veg + Sl + YSF + AllR_D_ |
| 111 | Open_500_ + RT_D_ + HC + Sl + Burn + AllR_D_ |
| 112 | Open_500_ + RT_D_ + Veg + Sl + Burn + AllR_D_ |
| 113 | Open_500_ + RT_D_ + Veg + Sl + YSF + AllR_D_ |
| 114 | RT_D_ + HC + Sl + YSF + AllR_D_ |
| 115 | RT_D_ + HC + Sl + AllR_D_ |
| 116 | RT_D_ + HC + Sl + YSF + AllR_D_ + W_D_ |
| 117 | HC + Sl + YSF + AllR_D_ |
| 118 | Open_500_ + RT_D_ + Veg + Sl + N + YSF + AllR_D_ |
| 119 | Open_500_ + RT_D_ + Veg + Sl + AllR_D_ |
| 120 | RT_D_ + Veg + Sl + AllR_D_ |
| 121 | RT_D_ + Veg + AllR_D_ |
| 122 | RT_D_ + Veg + Sl + AllR_D_ + Burn |
| 123 | RT_D_ + Veg + Sl + AllR_D_ + YSF |
| 124 | RT_D_ + Veg + Sl + AllR_D_ + Priv_D_ |
| 125 | Open_500_ + RT_D_ + W_D_ + Priv_D_ + Veg + Sl + N + AllR_D_ + YSF |
| 126 | Open_500_ + RT_D_ + R2_DEN_ + W_D_ + R3_D_ + Edge_D_ + VRM + VRM^2^ + Burn |
| 127 | RT_D_ + R2_DEN_ + W_D_ + R3_D_ + Edge_D_ + VRM + VRM^2^ + HC + Veg + Burn |

Model terms: Burn = burned in past 17 years (reference = unburned); W_D_ = distance to natural and artificial water bodies; El = elevation; R_DEN_ = density of road classes 1, 2, 3, or all classes; Open_500_ = number of open pixels in a 500 m rolling window; Priv_D_ = distance to private land; R_D_ = distance to road classes 1, 2, 3 or all classes; TRI = topographic ruggedness index; CC = percent canopy cover; HC = horizontal vegetation cover (reference = open); Sl = slope; Veg = dominant vegetation type (reference = grassland); VRM = vector ruggedness measure; YSF = years since most recent fire (reference = > 17 years/unburned); Fire = fire history combining burn severity and time since fire (reference = > 17 years/unburned); Edge_D_ = distance to forest edge; RT_D_ = distance to recreation sites and trails; N = northness index.

Table D2. Model comparisons by season Mexican wolf risky places models using locations where elk were killed by Mexican wolves in east-central Arizona and west-central New Mexico between 2015 and 2023. All models included a random intercept for unique pack ID (*n* = 32). K = degrees of freedom, AIC = Akaike’s Information Criterion score, ΔAIC = difference in AIC score relative to the top-ranked model, *w* = individual model weight, and LL = log likelihood.

| Model | K | AIC_C_ | ΔAIC_C_ | *w* | LL |
| --- | --- | --- | --- | --- | --- |
| Open_500_ + RT_D_ + Sl + Veg + Burn + AllR_D_ | 12 | 3615.05 | 0 | 0.75 | -1795.50 |
| RT_D_ + Sl + Veg + Burn + AllR_D_ | 11 | 3618.30 | 3.25 | 0.15 | -1798.13 |
| Open_500_ + RT_D_ + Sl + Veg + AllR_D_ | 11 | 3621.99 | 6.94 | 0.02 | -1799.97 |
| Open_500_ + RT_D_ + Sl + Veg + YSF + AllR_D_ | 14 | 3623.01 | 7.96 | 0.01 | -1797.47 |
| RT_D_ + W_D_ + Priv_D_ + Sl + Veg + N + Burn + AllR_D_ | 14 | 3623.17 | 8.13 | 0.01 | -1797.55 |
| CC + RT_D_ + W_D_ + Priv_D_ + Sl + Sl^2^ + Veg + N + Burn + AllR_D_ | 16 | 3623.91 | 8.86 | 0.01 | -1795.91 |
| Open_500_ + RT_D_ + AllR_DEN_ + W_D_ + Priv_D_ + Edge_D_ + Sl + Sl^2^ + Veg + N + Burn | 17 | 3624.18 | 9.13 | 0.01 | -1795.04 |
| Open_500_ + RT_D_ + Sl + Veg + N + YSF + AllR_D_ | 15 | 3624.67 | 9.62 | 0.01 | -1797.29 |
| Open_500_ + RT_D_ + AllR_DEN_ + W_D_ + Priv_D_ + Edge_D_ + Sl + Veg + N + Burn | 16 | 3624.78 | 9.73 | 0.01 | -1796.35 |
| Open_500_ + RT_D_ + Priv_D_ + Sl + Veg + YSF + AllR_D_ | 15 | 3625.02 | 9.97 | 0.01 | -1797.47 |

Model terms: Open_500_ = number of open pixels in a 500 m rolling window; RT_D_ = distance to recreation sites and trails; Sl = slope; Veg = dominant vegetation type (reference = grassland); Burn = burned in past 17 years (reference = unburned); R_DEN_ = density of road classes 1 (primary), 2 (secondary), 3 (tertiary/primitive), or all classes; R_D_ = distance to road classes 1, 2, 3 or all classes; W_D_ = distance to natural and artificial water bodies; Priv_D_ = distance to private land; N = northness index; CC = percent canopy cover; Edge_D_ = distance to forest edge; YSF = years since most recent fire (reference = > 17 years/unburned).

*Figures*


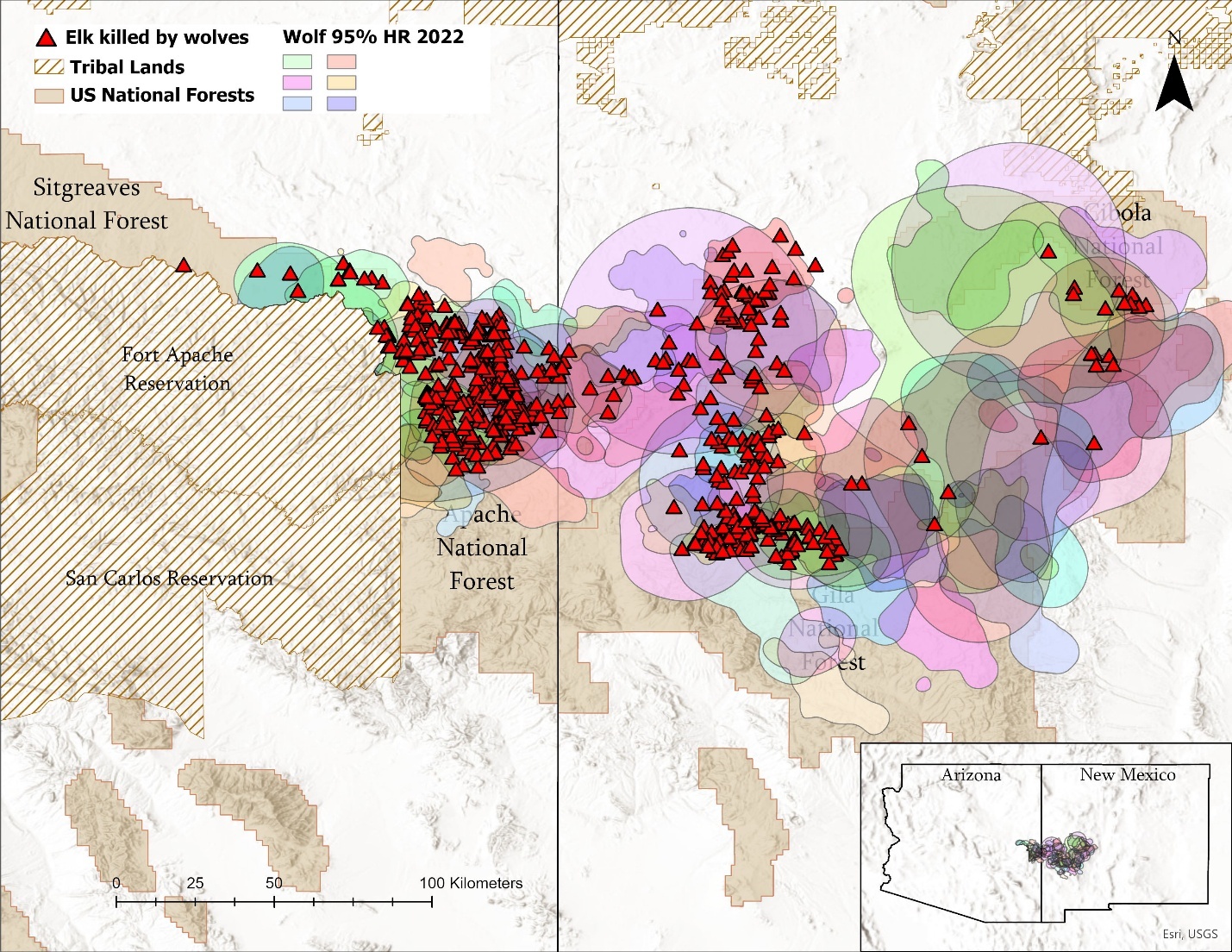


Figure D1. Locations of elk carcasses found at wolf clusters and of collared elk mortalities determined to have been killed by Mexican wolves from 2015-2023 in east-central Arizona and west-central New Mexico, with 95% KDE home range estimates for Mexican wolf packs for 2022. Data from tribal lands are excluded from figures based on an agreement between local tribes and USFWS.


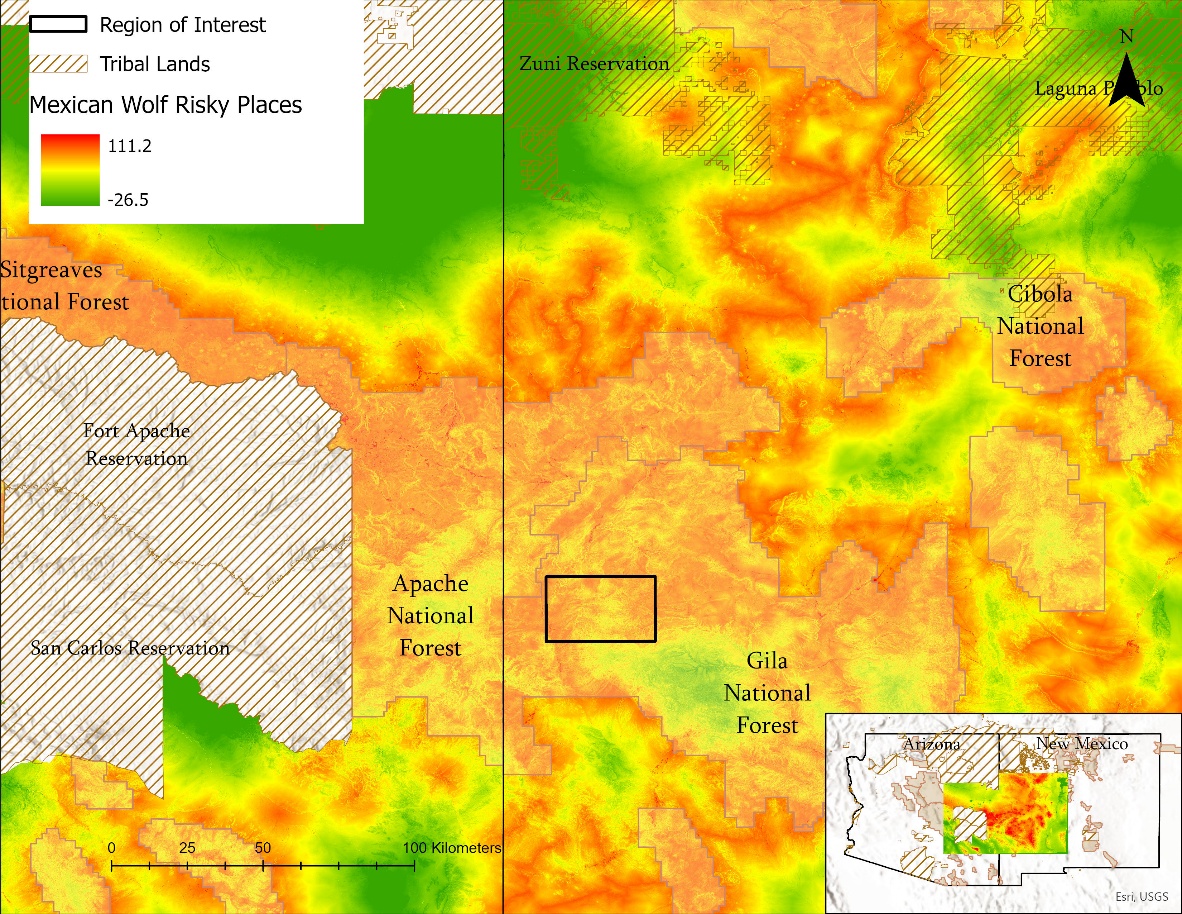

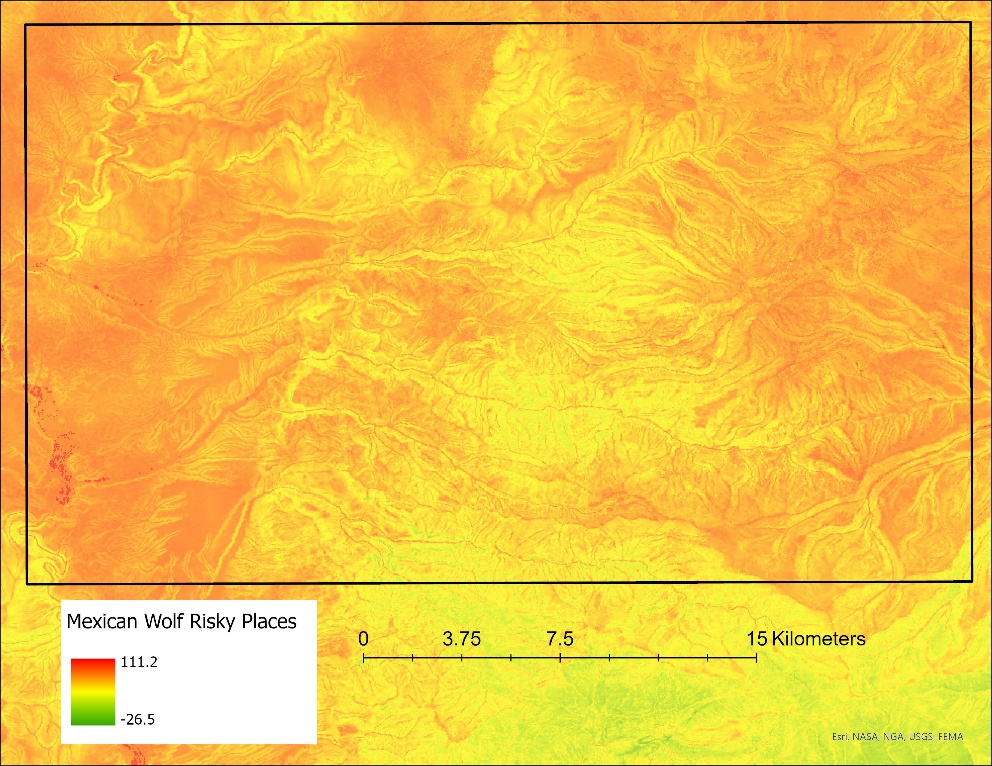


Figure D2. Mexican wolf predicted risky places for 2017-2023 in east-central Arizona and west-central New Mexico with red indicating higher probabilities of selection and green indicating lower probabilities of selection. The region of interest shows a portion of the study area in the Gila National Forest, NM. Data from tribal lands are excluded from figures based on an agreement between local tribes and USFWS.

*
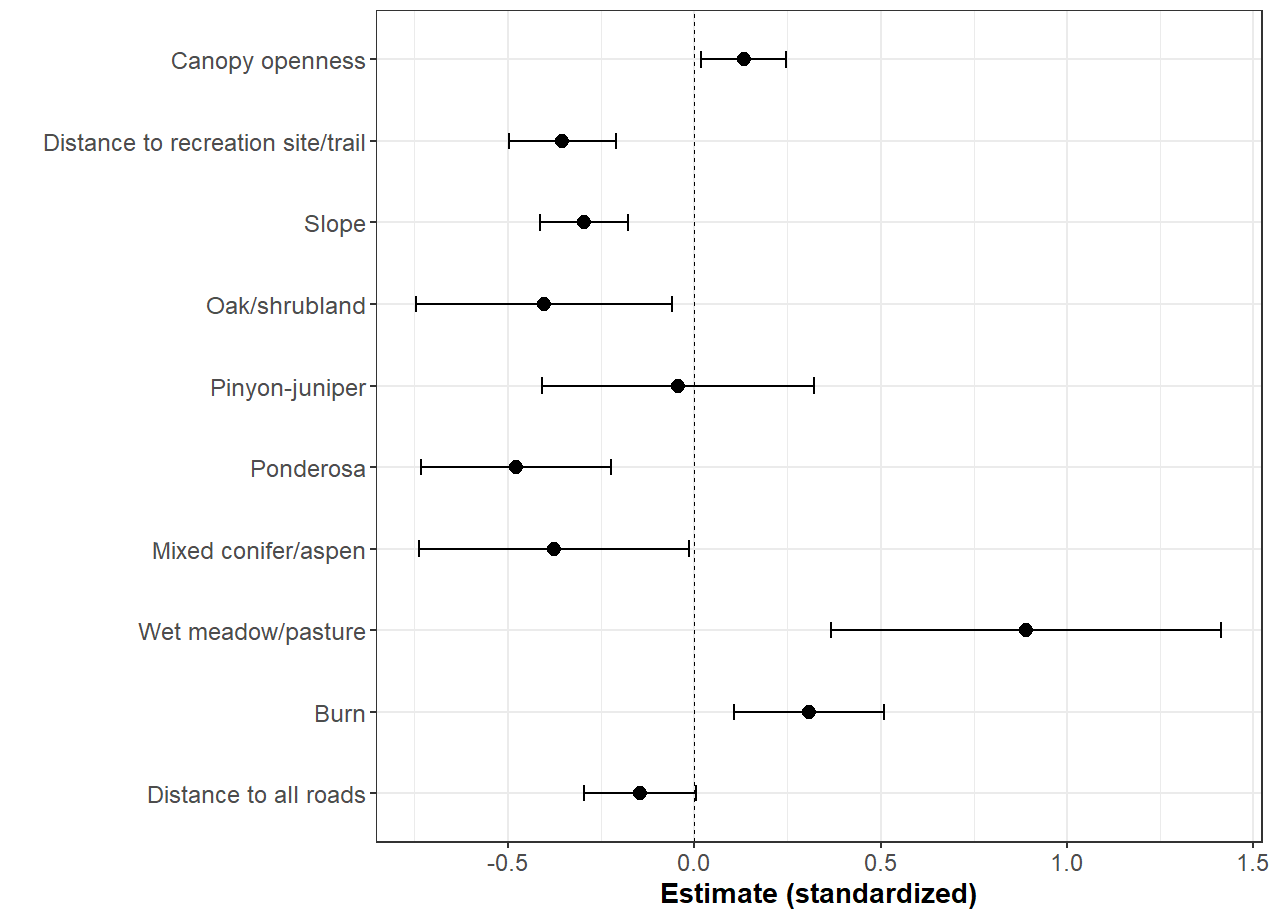
*

Figure D3: Standardized covariate effects for the top performing Mexican wolf risky places model of sites where elk were killed by wolves in east-central Arizona and west-central New Mexico between 2015 and 2023 with 95% SE confidence intervals. All variables were centered around zero and scaled prior to modeling. The reference category for dominant vegetation types was grassland, and for burn the reference category was unburned within 17 years.

SUPPLEMENTAL MATERIAL 5

MOUNTAIN LION RISKY PLACES INDEX

*Methods*

We used the locations of confirmed kills of elk by mountain lions, discovered either through collared elk mortality investigations or mountain lion cluster investigations, to model the characteristics of kill sites compared to available sites. We conducted cluster investigations between May 2021 and May 2023 and followed collared mountain lions in New Mexico. Collared individuals included both males and females and adults (>2.5 years old) and subadults (<2.5 years old); collars collected locations every hour. We followed each individual for ≥ 4 months and defined clusters as at least six points within 50 m and 48 hours of each other. We identified prey to species, age class, and sex. We prioritized clusters with more points or that began during crepuscular or night hours for investigation. If a female denned, we avoided clusters within 0.5 kilometers of the den site. We used VHF telemetry to check for the immediate presence of the collared individual to prevent an increase in kill rates due to pushing the individual off a carcass prematurely. At all clusters, we searched each point within a 50 m radius and walked between points methodically so that the entire area was searched. When carcasses were discovered, we took photos and conducted a necropsy.

*Analysis*

We included data from elk of all age classes (and included male elk due to small sample sizes in some seasons) killed either by a collared lion discovered at a cluster or by an unknown lion discovered through an elk collar mortality investigation. Mountain lions were only fitted with GPS collars in New Mexico, while elk were collared in Arizona and New Mexico and therefore kills by uncollared mountain lions were identified in both states. We assigned mountain lion kills based on evidence such as the presence of a drag trail or cache site, mountain lion scat or tracks near the carcass, bite marks to the neck or throat with the presence of hemorrhaging, a relatively intact carcass, and carcass entry through the ribcage or abdomen (Alt and Eckert 2017).

Because not all mortalities of collared elk could be assigned to a collared lion’s home range due to the presence of uncollared mountain lions, we did not sample random locations from home range polygons and instead calculated the average home range area for collared lions and used the radius of this area to create circular buffers centered on each kill location. We then sampled available points randomly at a 20:1 ratio to locations of elk kills and assigned a unique ID grouping the random locations with each kill, in the framework of a Type II habitat selection model design (Manly et al. 2002, Blake and Gese 2016). We subsequently removed any random points within twice the maximum cluster radius of each other or any kill sites then resampled the random locations to a ratio of 10:1.

We then buffered both kill site and available points by the maximum radius of any mountain lion cluster we investigated. Then for each of the buffered points, we extracted habitat covariates using the R package *terra* (Hijmans 2021). These covariates were distance to road of three categories (primary, secondary, and primitive), densities of roads, elevation, slope, northness index, VRM, topographic position index (TPI), horizontal cover, vegetation cover type, human density, distance to ecotone, distance to private land, burn history, canopy openness, and distance to recreation sites or trails (see Supplementary Material 3; Supplementary Material 1 – Table 2). We assessed all continuous covariates for multicollinearity and when the Spearman’s Rho value was greater than 0.6, did not include those two covariates in the same model. We centered and scaled all continuous covariates prior to modeling.

We constructed 33 *a priori* conditional logistic regression models stratified by each unique kill site and its associated random locations using the *survival* package in R (Therneau 2023; Table E1). For the response variable, we represented kill sites with 1 and available sites with 0. We used Akaike’s Information Criterion for small sample sizes (AIC_C_) to select the top-performing models and considered models competing if they were within 2.5 ΔAIC_C_ (Hilbe 2011). To assess the performance of the top-ranked model, we performed a 5-fold cross validation with 100 iterations and calculated a Spearman-rank correlation to assess the predictive performance of the model across 10 bins (Boyce et al. 2002). We also calculated VIF scores for the top-ranked model to ensure collinearity of covariates remained low.

*Results*

We included locations for 280 elk killed by mountain lions found at cluster investigations of 23 individual collared lions and 93 locations of collared elk whose causes of mortality were attributed to mountain lion kills, for a total of 373 kill sites and 3,730 available locations (Figure E1). Most kills were neonates (*n* = 276; 74%). Of the adult elk killed (*n* = 97; 26%), the majority were female (*n* = 82; 85%) with a small number of males (*n* = 11; 11%) and unknowns (*n* = 4; 4%). The average home range radius for collared mountain lions used to create the available area buffers was 18.86 kilometers and the maximum cluster radius used to create buffers for each point was 155 meters.

The top model for places with high mountain lion kill risk included quadratic terms for elevation and terrain ruggedness, distance to water, forest edges, and recreation sites/trails, obstructive vegetation, and the density of high-use, maintained roads (Table E2; Figure E2). VIF scores for all model terms were less than 2, indicating that covariates were not significantly correlated. The Spearman’s-rank rho value calculated from the 5-fold cross validation of the top model was 0.964. The odds of a location being a mountain lion kill site were higher at moderate elevations and moderate terrain ruggedness, and increased with proximity to water, forest edges, and recreation sites and trails (Figure E3). Additionally, the odds of a kill were higher in areas with obstructive vegetation (taller than 1 m) and lower in areas with high densities of human use and maintained roads (type 1). Terrain ruggedness and road density were the most influential terms in the model.

*Literature Cited*

Alt, K., and M. Eckert. 2017. Predation ID manual: predator kill and scavenging characteristics. Simon and Schuster.

Blake, L. W., and E. M. Gese. 2016. Resource selection by cougars: Influence of behavioral state and season: Cougar Resource Selection. Journal of Wildlife Management 80:1205–1217.

Boyce, M. S., P. R. Vernier, S. E. Nielsen, and F. K. A. Schmiegelow. 2002. Evaluating resource selection functions. Ecological Modelling 157:281–300.

Hijmans, R. J. 2021. terra: Spatial data analysis. R package version 1.4-7. <https://CRAN.R-project.org/package=terra>.

Hilbe, J. M. 2011. Negative Binomial Regression. 2nd edition. Cambridge University Press.

Manly, B. F. J., L. L. McDonald, D. L. Thomas, T. L. McDonald, and W. P. Erickson. 2002. Resource selection by animals: statistical design and analysis for field studies. Second edition. Springer Science & Business Media.

Therneau, T. 2023. A package for survival analysis in R. <https://CRAN.R-project.org/package=survival>.

*Tables*

Table E1. Model structures tested for the mountain lion risky places index using sites where elk were killed by mountain lions in east-central Arizona and west-central New Mexico between 2019 and 2023. All models were stratified by kill site ID.

| Model Structure | |
| --- | --- |
| 1 | TRI + TRI^2^ + TPI + AllR_DEN_ + CC + TPI*CC + AllR_DEN_*CC |
| 2 | TRI + TRI^2^ + TPI + AllR_DEN_ + CC + VH + TPI*VH + AllR_DEN_*VH |
| 3 | El + El^2^ + TRI + TRI^2^ + AllR_DEN_ + W_D_ + N + Veg |
| 4 | Human_DEN_ + Veg |
| 5 | AllR_DEN_ + Veg |
| 6 | El + TRI + TRI^2^ + AllR_DEN_ + CC + VH |
| 7 | El + El^2^ + TRI + TRI^2^ + AllR_DEN_ + BS_MAX_ |
| 8 | El + TRI + TRI^2^ + AllR_DEN_ + BS_MAX_ |
| 9 | El + TRI + TRI^2^ + AllR_DEN_ + BS_MAX_ + BS_MAX_ *AllR_DEN_ |
| 10 | El + TRI + TRI^2^ + Edge_D_ + BS_MAX_ |
| 11 | El + TRI + TRI^2^ + Human_DEN_ |
| 12 | El + TRI + TRI^2^ + Human_DEN_ + BS_MAX_ |
| 13 | El + TRI + TRI^2^ + TPI + AllR_DEN_ + CC + W_D_ + VH + N + Edge_D_ + BS_MAX_ + AllR_DEN_*VH |
| 14 | El + TRI + TRI^2^ + TPI + AllR_DEN_ + CC + W_D_ + VH + N + Edge_D_ + BS_MAX_ + CC* BS_MAX_ |
| 15 | El + El^2^ + TRI + TRI^2^ + AllR_DEN_ + W_D_ + N + Veg + RT_D_ |
| 16 | El + El^2^ + TRI + TRI^2^ + AllR_DEN_ + W_D_ + N + Veg + RT_D_ + BS_MAX_ |
| 17 | El + TRI + TRI^2^ + AllR_DEN_ |
| 18 | El + AllR_DEN_ + TPI + W_D_ |
| 19 | TRI + TRI^2^ + AllR_DEN_ + W_D_ + VH + AllR_DEN_*VH |
| 20 | El + TRI + TRI^2^ + TPI + AllR_DEN_ + CC + W_D_ + VH + N + Edge_D_ + BS_MAX_ |
| 21 | El + El^2^ + TRI + TRI^2^ + TPI + AllR_DEN_ + CC + W_D_ + VH + N + Edge_D_ + BS_MAX_ + CC* BS_MAX_ |
| 22 | El + El^2^ + TRI + TRI^2^ + TPI + AllR_DEN_ + CC + W_D_ + VH + N + Edge_D_ + BS_MAX_ |
| 23 | El + El^2^ + TRI + TRI^2^ + TPI + AllR_DEN_ + Veg + RT­_D_ + N + Edge_D_ + BS_MAX_ |
| 24 | El + El^2^ + TRI + TRI^2^ + TPI + AllR_DEN_ + CC + Veg + RT­_D_ + N + Edge_D_ + BS_MAX_ + CC* BS_MAX_ |
| 25 | El + TRI + TRI^2^ + TPI + AllR_DEN_ + CC + W_D_ + VH + N + Edge_D_ + BS_MAX_ |
| 26 | El + TRI + TRI^2^ + TPI + AllR_DEN_ + CC + W_D_ + VH + N + Edge_D_ + BS_MAX_ + CC* BS_MAX_ |
| 27 | El + El^2^ + TRI + TRI^2^ + TPI + CC + W_D_ + VH + Edge_D_ + BS_MAX_ |
| 28 | El + El^2^ + TRI + TRI^2^ + AllR_DEN_ + CC + NDVI + W_D_ + VH + RT_D_ + NDVI*VH |
| 29 | El + El^2^ + TRI + TRI^2^ + AllR_DEN_ + NDVI + W_D_ + VH + RT_D_ + NDVI*VH |
| 30 | El + El^2^ + TRI + TRI^2^ + R1_DEN_ + CC + W_D_ + VH + RT_D_ + Edge_D_ + BS_MAX_ + CC*BS_MAX_ |
| 31 | El + El^2^ + TRI + TRI^2^ + R1_DEN_ + CC + W_D_ + VH + RT_D_ + Edge_D_ + BS_MAX_ |
| 32 | El + El^2^ + TRI + TRI^2^ + R1_DEN_ + W_D_ + VH + RT_D_ + Edge_D_ |
| 33 | El + El^2^ + TRI + TRI^2^ + R1_DEN_ + W_D_ + Veg + RT_D_ + Edge_D_ + Burn |

Model terms: TRI = topographic ruggedness index; TPI = topographic position index; R_DEN_ = density of class 1 or all road classes; CC = percent canopy cover; VH = classified vegetation height (reference = 0-0.5 m); El = elevation; W_D_ = distance to natural and artificial water bodies; N = northness index; Veg = dominant vegetation type (reference = grassland); Human_DEN_ = human density; BS_MAX_ = maximum burn severity; Edge_D_ = distance to forest edge; RT_D_ = distance to recreation sites and trails; Burn = burned in past 17 years (reference = unburned).

Table E2. Model comparison for mountain lion risky places conditional logistic regression models using sites where elk were killed by mountain lions in east-central Arizona and west-central New Mexico between 2019 and 2023. All models were stratified by kill site ID. K = degrees of freedom, AIC = Akaike’s Information Criterion score, ΔAIC = difference in AIC score relative to the top-ranked model, *w* = individual model weight, and LL = log likelihood.

| Model | K | AIC­­_C_ | ΔAIC_C_ | *w* | LL |
| --- | --- | --- | --- | --- | --- |
| El + El^2^ + TRI + TRI^2^ + R1_DEN_ + W_D_ + VH + RT_D_ + Edge_D_ | 9 | 1652.07 | 0.00 | 0.71 | -816.78 |
| El + El^2^ + TRI + TRI^2^ + R1_DEN_ + CC + W_D_ + VH + RT_D_ + Edge_D_ + BS_MAX_ + CC*BS_MAX_ | 13 | 1654.12 | 2.13 | 0.25 | -813.59 |
| El + El^2^ + TRI + TRI^2^ + R1_DEN_ + W_D_ + Veg + RT_D_ + Edge_D_ + Burn | 13 | 1658.26 | 6.20 | 0.03 | -815.62 |
| El + El^2^ + TRI + TRI^2^ + R1_DEN_ + CC + W_D_ + VH + RT_D_ + Edge_D_ + BS_MAX_ | 16 | 1660.03 | 7.97 | 0.01 | -813.25 |
| El + TRI + TRI^2^ + TPI + AllR_DEN_ + CC + W_D_ + VH + N + Edge_D_ + BS_MAX_ + CC* BS_MAX_ | 11 | 1674.32 | 22.25 | 0.00 | -825.79 |
| El + El^2^ + TRI + TRI^2^ + AllR_DEN_ + CC + NDVI + W_D_ + VH + RT_D_ + NDVI*VH | 10 | 1677.01 | 24.95 | 0.00 | -828.20 |
| El + El^2^ + TRI + TRI^2^ + TPI + AllR_DEN_ + CC + Veg + RT­_D_ + N + Edge_D_ + BS_MAX_ + CC*BS_MAX_ | 16 | 1679.19 | 27.12 | 0.00 | -822.82 |
| El + El^2^ + TRI + TRI^2^ + TPI + AllR_DEN_ + Veg + RT­_D_ + N + Edge_D_ + BS_MAX_ | 12 | 1679.28 | 27.21 | 0.00 | -827.20 |
| El + El^2^ + TRI + TRI^2^ + TPI + CC + W_D_ + VH + Edge_D_ + BS_MAX_ | 12 | 1679.46 | 27.39 | 0.00 | -827.29 |
| El + El^2^ + TRI + TRI^2^ + AllR_DEN_ + NDVI + W_D_ + VH + RT_D_ + NDVI*VH | 15 | 1680.45 | 28.38 | 0.00 | -824.55 |

Model terms: El = elevation; TRI = topographic ruggedness index; R_DEN_ = density of class 1 or all road classes; W_D_ = distance to natural and artificial water bodies; VH = classified vegetation height (reference = 0-0.5 m); RT_D_ = distance to recreation sites and trails; Edge_D_ = distance to forest edge; CC = percent canopy cover; BS_MAX_ = maximum burn severity; Veg = dominant vegetation type (reference = grassland); Burn = burned in past 17 years (reference = unburned); TPI = topographic position index; N = northness index; NDVI = normalized difference vegetation index.

*Figures*


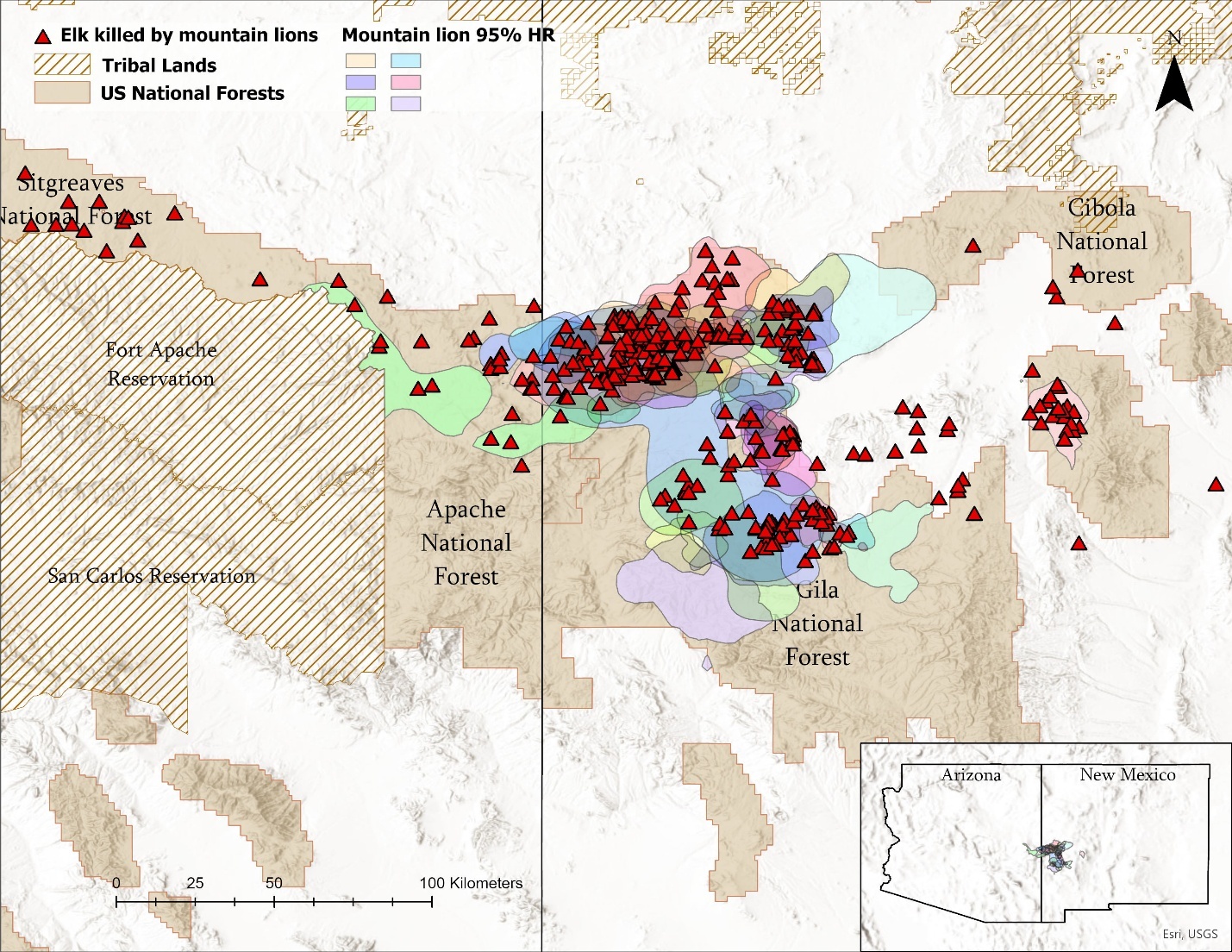


Figure E1. Locations of elk carcasses found at mountain lion clusters and of collared elk mortalities determined to have been killed by mountain lions from 2019-2023 in east-central Arizona and west-central New Mexico, with 95% KDE home range estimates for mountain lions. Data from tribal lands are excluded from figures based on an agreement between local tribes and USFWS.


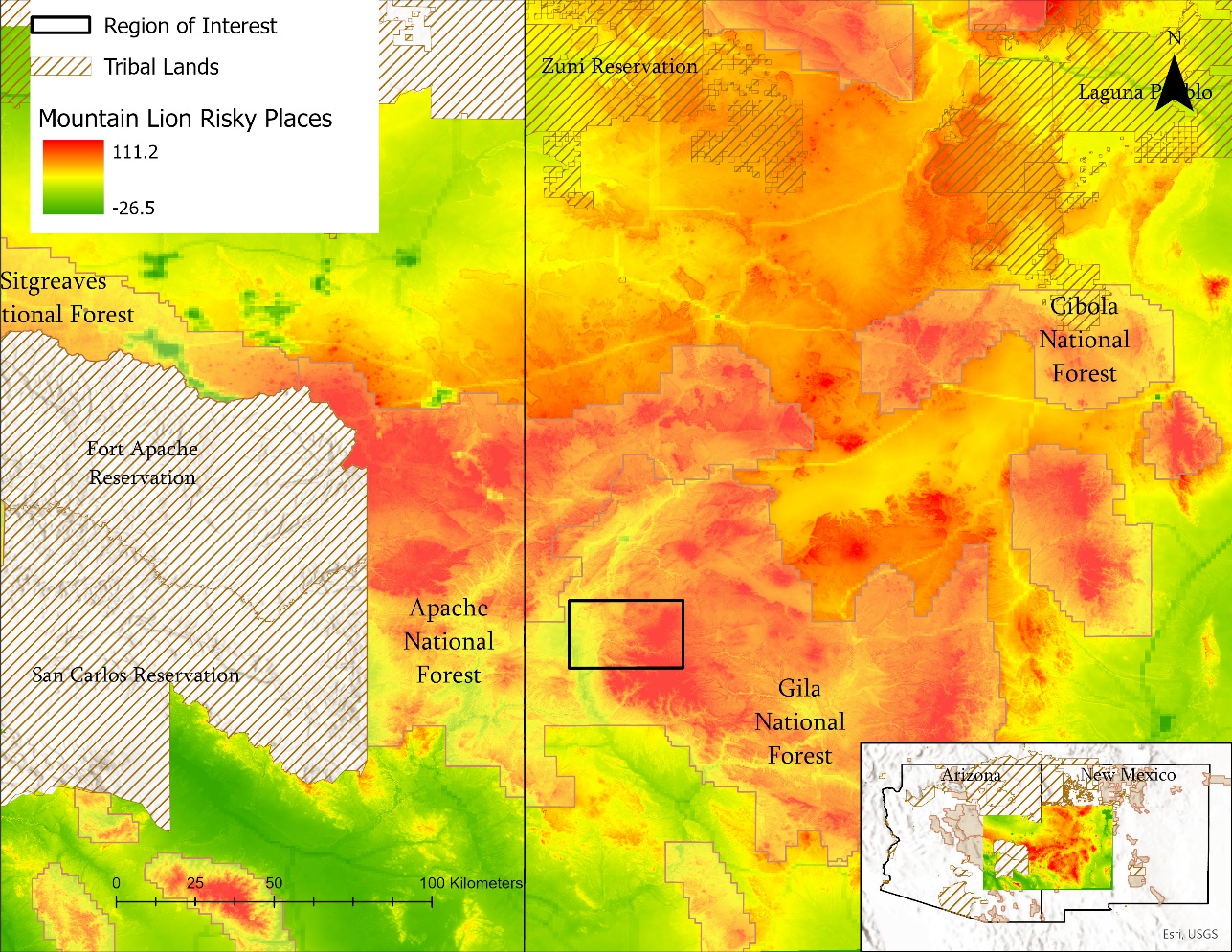

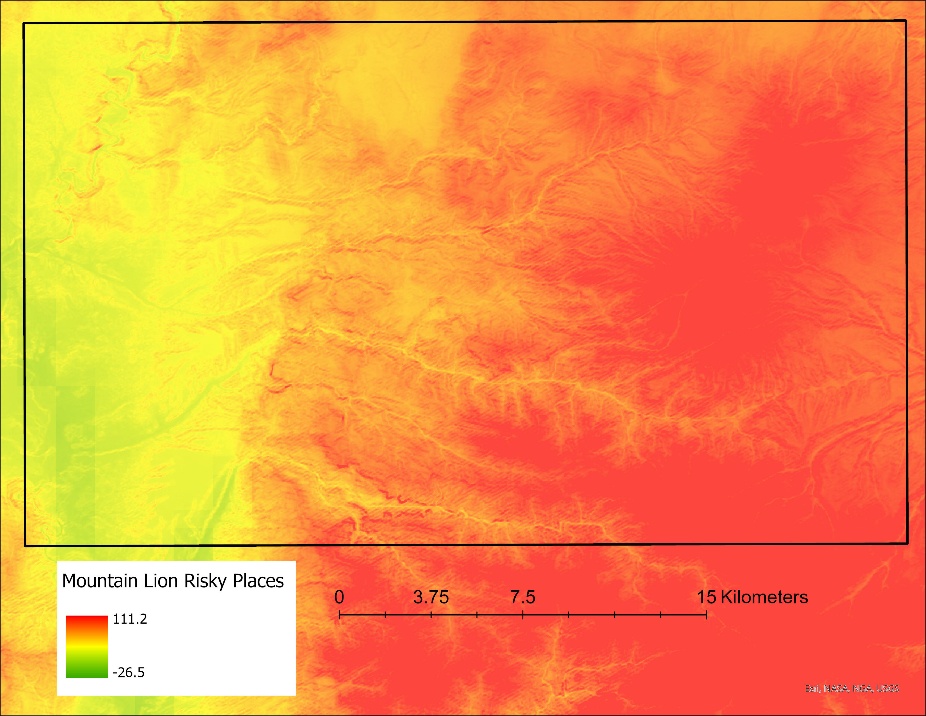


Figure E2. Mountain lion predicted risky places for 2021-2023 in east-central Arizona and west-central New Mexico with red indicating higher probabilities of selection and green indicating lower probabilities of selection. The region of interest shows a portion of the study area in the Gila National Forest, NM. Data from tribal lands are excluded from figures based on an agreement between local tribes and USFWS


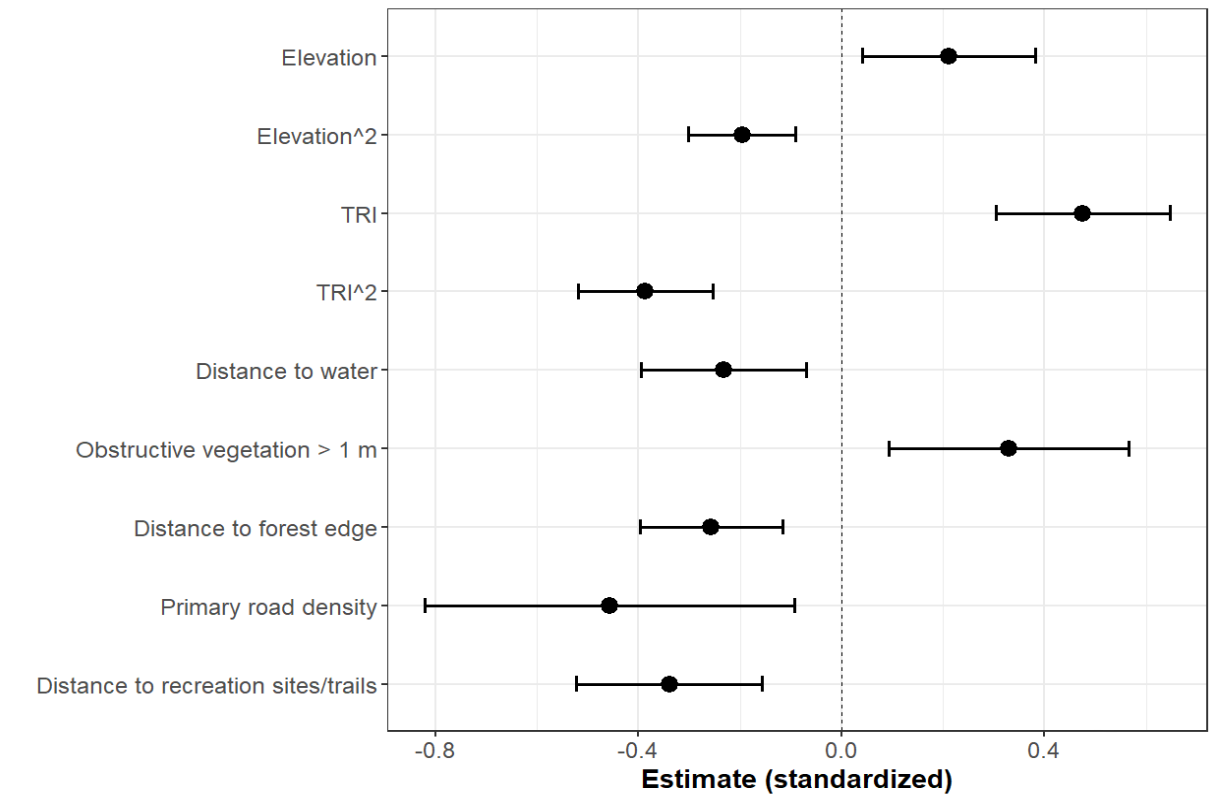


Figure E3. Standardized regression coefficients for the top mountain lion risky places model using locations where elk were killed by mountain lions in east-central Arizona and west-central New Mexico between 2019-2023 with 95% confidence intervals.

SUPPLEMENTAL MATERIAL 6

BAYESIAN MODEL STRUCTURES, DIAGNOSTICS, MODEL SELECTION, AND ADDITIONAL TABLES AND FIGURES

*Tables*

Table F1. Base *a priori* model structures for overall individual elk behavior and foraging proportion models from focal samples.

|  | Model structure | Model variables |
| --- | --- | --- |
| 0 | Null | Intercept |
| 1 | Temporal + Social + Environmental + Human | S + Diel + Herd + Calf + NDVI + R3_D_ + Human |
| 2 | Temporal + Social | S + Diel + Herd + Calf |
| 3 | Temporal + Social + Temporal*Social | S + Diel + Herd + Calf + S*Herd |
| 4 | Temporal + Social + Human | S + Diel + Herd + Calf + R3_D_ + Human |
| 5 | Social + Human | Herd + Calf + R3_D_ + Human |
| 6 | Temporal + Social + Human + Temporal*Social | S + Diel + Herd + Calf + R3_D_ + Human + S*Herd |
| 7 | Temporal + Social + Human + Temporal*Human | S + Diel + Herd + Calf + R3_D_ + Human + S*Human |
| 8 | Temporal + Environmental | S + Diel + NDVI |

Model terms: S = Season; Diel = diel period (reference = day); Herd = herd size; Calf = ratio of calves to cows in the herd; NDVI = normalized difference vegetation index; R3_D_ = distance to unmaintained roads; Human = human density.

Table F2. Predator effect model structures tested on the top base model for each behavioral model type. The inclusion of wolf distance covariates in model 8 was based on preliminary rankings of models 1-6.

| Model Structure | |
| --- | --- |
| 1 | Base + **Wolf encounter** |
| 2 | Base + **Wolf kill** |
| 3 | Base + **Mountain lion encounter** |
| 4 | Base + **Mountain lion kill** |
| 5 | Base + **Wolf distance** |
| 6 | Base + **Wolf encounter** + **Wolf distance** |
| 7 | Base + **Wolf encounter** + **Wolf distance** + **Wolf encounter * Wolf distance** |
| 8 | Base + **Wolf encounter** + **Mountain lion** +/- **Wolf distance** +/- **Wolf encounter * Wolf distance** |

Wolf/mountain lion encounter = risk of wolf or mountain lion encounter based on wolf/mountain lion Habitat selection Function (HSF) or HSF multiplied by Utilization Distribution (UD) and pack size for wolves. Wolf/mountain lion kill = risk of being killed by wolf or mountain lion based on risky places regression models or risky places models multiplied by UD and pack size for wolves. Wolf distance = distance to nearest collared wolf in 24 hours prior to observation. +/- indicates this term was sometimes included or not included depending on the performance of the term across model structures.

Table F3. Minimum tail effective sample sizes (ESS) and maximum Rhat values for all parameter estimates and the number of estimates with Pareto *k* > 0.7 out of the total number of samples from PSIS-LOO cross validation for the top-ranked model in each response type for elk foraging behavior in east-central Arizona and west-central New Mexico between 2022-2023.

| Response type | Min. tail ESS | Max. Rhat | *n* Pareto k > 0.7 |
| --- | --- | --- | --- |
| Individual | 12,438 | 1.00 | 2/595 |
| FIR | 12,261 | 1.00 | 0/595 |
| AFA | 13,777 | 1.00 | 1/580 |
| Multitask | 10,446 | 1.00 | 0/3054 |
| Herd | 14,441 | 1.00 | 3/352 |

Table F4. Model comparison for overall individual elk behavior. Model performance was assessed using Pareto-Smoothed Importance Sampling-Leave One Out cross validation, and models with standard error differences of the Expected Log Pointwise Predictive Density score (ΔSE ELPD) larger than the ΔELPD were considered competing (unshaded in table). Model weights (*w*) were estimated using Pseudo-Bayesian model averaging. Predation risk metrics are shown in bold.

| Model | ΔELPD | ΔSE | ELPD | SE | *w* |
| --- | --- | --- | --- | --- | --- |
| S + Diel + Herd + Calf + R3_D_ + Human + S*Herd + Wolf encounter | 0.00 | 0.00 | 5675.44 | 96.33 | 0.42 |
| S + Diel + Herd + Calf + R3_D_ + Human + S*Herd + Wolf kill | -0.49 | 1.11 | 5674.95 | 96.32 | 0.25 |
| S + Diel + Herd + Calf + R3_D_ + Human + S*Herd | -1.00 | 2.68 | 5674.45 | 96.16 | 0.15 |
| S + Diel + Herd + Calf + R3_D_ + Human + S*Herd + Lion encounter | -1.67 | 2.89 | 5673.77 | 96.20 | 0.08 |
| S + Diel + Herd + Calf + R3_D_ + Human + S * Herd + Wolf encounter + Lion encounter | -2.26 | 1.33 | 5673.19 | 96.38 | 0.04 |
| S + Diel + Herd + Calf + R3_D_ + Human + S * Herd + Wolf encounter + Wolf_D_ | -2.36 | 1.52 | 5673.08 | 96.426 | 0.04 |
| S + Diel + Herd + Calf + R3_D_ + Human + S * Herd + Wolf_D_ | -4.24 | 2.94 | 5671.20 | 96.18 | 0.01 |
| S + Diel + Herd + Calf + R3_D_ + Human + S * Herd + Lion kill | -4.30 | 2.91 | 5671.14 | 96.23 | 0.01 |
| Herd + Calf + R3_D_ + Human | -5.11 | 8.99 | 5670.33 | 96.70 | 0.00 |
| S + Diel + Herd + Calf + R3_D_ + Human + S*Herd + Wolf encounter + Wolf_Dcat_ + Wolf encounter*Wolf_Dcat_ | -5.12 | 2.27 | 5670.33 | 96.46 | 0.00 |
| S + Diel + Herd + Calf + R3_D_ + Human | -5.91 | 6.88 | 5669.54 | 96.45 | 0.00 |
| S + Diel + Herd + Calf + S*Herd | -7.88 | 8.12 | 5667.56 | 96.15 | 0.00 |
| S + Diel + Herd + Calf + NDVI + R3_D_ + Human | -8.80 | 7.07 | 5666.64 | 96.47 | 0.00 |
| S + Diel + Herd + Calf | -11.93 | 10.35 | 5663.51 | 96.45 | 0.00 |
| Null | -13.91 | 12.45 | 5661.53 | 96.56 | 0.00 |
| S + Diel + Herd + Calf + R3_D_ + Human + S*Human | -14.43 | 9.12 | 5661.02 | 96.77 | 0.00 |
| S + Diel + NDVI | -17.95 | 11.32 | 5657.49 | 96.26 | 0.00 |

Model terms: S = season; Diel = diel period; Herd = herd size; Calf = calf:cow ratio; R3_D_ = distance to unmaintained roads; Human = human density; Wolf encounter = Mexican wolf HSF weighted by yearly pack UD and average yearly pack sizes; Wolf kill = Mexican wolf risky places model weighted by yearly pack UD and average yearly pack sizes; Lion encounter = unweighted mountain lion HSF; Wolf_D_ = Mexican wolf distance in past 24 hours; Lion kill = unweighted mountain lion risky places model; Wolf_Dcat_ = categorical Mexican wolf distance (1 = collared wolf was within 3 km in past 24 hours, 0 = no collared wolf was within 3 km in past 24 hours); NDVI = normalized difference vegetation index.

Table F5. Model comparison for individual elk Forage Intake Rate (FIR), calculated as the proportion of time spent foraging during a foraging bout. Model performance was assessed using Pareto-Smoothed Importance Sampling-Leave One Out cross validation, and models with standard error differences of the Expected Log Pointwise Predictive Density score (ΔSE ELPD) larger than the ΔELPD were considered competing (unshaded in table). Model weights (*w*) were estimated using Pseudo-Bayesian model averaging. Parameters that were universally uninformative (Human and Calf) were removed from all model structures except when included in an interaction. Predation risk metrics are shown in bold.

| Model | ΔELPD | ΔSE | ELPD | SE | *w* |
| --- | --- | --- | --- | --- | --- |
| S + Diel + Herd + R3_D_ + S*Herd + Lion encounter + Wolf kill + Wolf_D_ | 0.00 | 0.00 | 184.23 | 18.93 | 0.20 |
| S + Diel + Herd + R3_D_ + S*Herd + Lion encounter | -0.32 | 1.89 | 183.91 | 18.56 | 0.14 |
| S + Diel + Herd + R3_D_ + S*Herd | -0.43 | 2.40 | 183.80 | 18.52 | 0.13 |
| S + Diel + Herd + R3_D_ + S*Herd + Wolf encounter + Wolf_D_ | -0.45 | 1.20 | 183.78 | 18.84 | 0.13 |
| S + Diel + Herd + R3_D_ + S*Herd + Wolf_D_ | -0.58 | 2.03 | 183.64 | 18.63 | 0.11 |
| S + Diel + Herd + R3_D_ + S*Herd + Wolf kill | -0.59 | 2.06 | 183.64 | 18.66 | 0.11 |
| S + Diel + Herd + R3_D_ + S*Herd + Wolf encounter | -0.73 | 2.07 | 183.50 | 18.64 | 0.10 |
| S + Diel + Herd + R3_D_ + S*Herd + Lion kill | -1.62 | 2.58 | 182.60 | 18.51 | 0.04 |
| S + Diel + Herd + R3_D_ + S*Herd + Wolf encounter + Wolf_Dcat_ + Wolf encounter* Wolf_Dcat_ | -1.680 | 2.11 | 182.55 | 18.88 | 0.04 |
| S + Diel + Herd + R3_D_ | -3.52 | 4.45 | 180.70 | 18.29 | 0.01 |
| S + Diel + Herd + NDVI + R3_D_ | -4.48 | 4.44 | 179.74 | 18.33 | 0.00 |
| S + Diel + Herd + R3_D_ + Human + S*Human | -4.84 | 5.07 | 179.39 | 18.51 | 0.00 |
| Herd + R3_D_ | -5.57 | 6.24 | 178.65 | 18.22 | 0.00 |
| S + Diel + Herd + S*Herd | -10.63 | 6.56 | 173.59 | 18.33 | 0.00 |
| S + Diel + Herd | -12.96 | 7.65 | 171.27 | 18.21 | 0.00 |
| Null | -13.16 | 8.61 | 171.06 | 17.87 | 0.00 |
| S + Diel + NDVI | -13.40 | 7.72 | 170.83 | 18.18 | 0.00 |

Model terms: S = season; Diel = diel period; Herd = herd size; R3_D_ = distance to unmaintained roads; Lion encounter = unweighted mountain lion HSF; Wolf kill = Mexican wolf risky places model weighted by yearly pack UD and average yearly pack sizes; Wolf_D­­_ = distance to nearest collared Mexican wolf in the past 24 hours; Wolf encounter = Mexican wolf HSF weighted by yearly pack UD and average yearly pack sizes; Lion kill = unweighted mountain lion risky places model; Wolf_Dcat_ = categorical Mexican wolf distance (1 = collared wolf was within 3 km in past 24 hours, 0 = no collared wolf was within 3 km in past 24 hours); NDVI = normalized difference vegetation index; Human = human density.

Table F6. Model comparison for individual elk Accepted Food Abundance (AFA), calculated as the number of steps an individual took per minute while actively foraging. Model performance was assessed using Pareto-Smoothed Importance Sampling-Leave One Out cross validation, and models with standard error differences of the Expected Log Pointwise Predictive Density score (ΔSE ELPD) larger than the ΔELPD were considered competing (unshaded in table). Model weights (*w*) were estimated using Pseudo-Bayesian model averaging. Predation risk metrics are shown in bold.

| Model | ΔELPD | ΔSE | ELPD | SE | *w* |
| --- | --- | --- | --- | --- | --- |
| S + Diel + Herd + Calf + S*Herd + Lion kill | 0.00 | 0.00 | -2039.04 | 45.38 | 0.32 |
| S + Diel + Herd + Calf + S*Herd | -0.31 | 2.64 | -2039.35 | 46.77 | 0.23 |
| S + Diel + Herd + Calf + S*Herd + Lion encounter | -0.35 | 1.64 | -2039.39 | 45.97 | 0.22 |
| S + Diel + Herd + Calf + S*Herd + Wolf kill + Lion kill | -1.59 | 1.74 | -2040.63 | 46.38 | 0.06 |
| S + Diel + Herd + Calf + S*Herd + Wolf_D_ | -1.81 | 3.11 | -2040.85 | 47.21 | 0.05 |
| S + Diel + Herd + Calf + S*Herd + Wolf kill | -1.90 | 3.23 | -2040.94 | 47.29 | 0.05 |
| S + Diel + Herd + Calf + S*Herd + Wolf encounter | -2.24 | 3.28 | -2041.28 | 47.41 | 0.03 |
| S + Diel + Herd + Calf + R3_D_ + Human + S*Herd | -2.46 | 3.44 | -2041.50 | 46.93 | 0.03 |
| S + Diel + Herd + Calf + S*Herd + Wolf kill + Wolf_D_ | -4.47 | 5.22 | -2043.51 | 49.01 | 0.00 |
| S + Diel + Herd + Calf + S*Herd + Wolf kill + Wolf_Dcat_ + Wolf kill*Wolf_Dcat_ | -4.95 | 4.21 | -2043.99 | 47.97 | 0.00 |
| S + Diel + Herd + Calf + R3_D_ + Human + S*Human | -5.60 | 11.04 | -2044.64 | 48.32 | 0.00 |
| S + Diel + Herd + Calf | -12.76 | 9.06 | -2051.80 | 49.33 | 0.00 |
| S + Diel + NDVI | -12.88 | 9.27 | -2051.91 | 49.11 | 0.00 |
| S + Diel + Herd + Calf + R3_D_ + Human | -12.95 | 9.53 | -2051.99 | 48.99 | 0.00 |
| S + Diel + Herd + Calf + NDVI + R3_D_ + Human | -13.29 | 9.75 | -2052.33 | 48.79 | 0.00 |
| Null | -20.66 | 14.28 | -2059.70 | 49.72 | 0.00 |
| Herd + Calf + R3_D_ + Human | -21.87 | 13.02 | -2060.91 | 50.82 | 0.00 |

Model terms: S = season; Diel = diel period; Herd = herd size; Calf = calf:cow ratio; Lion kill = mountain lion risky places model weighted by seasonal population UD; Lion encounter = mountain lion HSF weighted by seasonal population UD; Wolf kill = Mexican wolf risky places model weighted by yearly pack UD and maximum yearly pack sizes; Wolf_D­­_ = distance to nearest collared Mexican wolf in the past 24 hours; Wolf encounter = Mexican wolf HSF weighted by yearly pack UD and maximum yearly pack sizes; Wolf_Dcat_ = categorical Mexican wolf distance (1 = collared wolf was within 3 km in past 24 hours, 0 = no collared wolf was within 3 km in past 24 hours); R3_D_ = distance to unmaintained roads; Human = human density; NDVI = normalized difference vegetation index.

Table F7. Model comparison for probability of individual elk multitasking. Model performance was assessed using Pareto-Smoothed Importance Sampling-Leave One Out cross validation, and models with standard error differences of the Expected Log Pointwise Predictive Density score (ΔSE ELPD) larger than the ΔELPD were considered competing (unshaded in table). Model weights (*w*) were estimated using Pseudo-Bayesian model averaging. Distance to unmaintained road (R3_D_) was universally uninformative and was removed from all model structures. All model structures included a random intercept for individual elk. Predation risk metrics are shown in bold.

| Model | ΔELPD | ΔSE | ELPD | SE | *w* |
| --- | --- | --- | --- | --- | --- |
| S + Diel + Herd + Calf + Human + S*Calf + Wolf_UDy_ + Wolf_Dcat_ + Lion encounter + Wolf_UDy_*Wolf_Dcat_ | 0.00 | 0.00 | -1918.50 | 20.01 | 0.48 |
| S + Diel + Herd + Calf + Human + S*Calf + Wolf_UDy_ + Lion encounter | -0.56 | 2.02 | -1919.06 | 19.82 | 0.28 |
| S + Diel + Herd + Calf + Human + S*Calf + Lion encounter | -1.76 | 2.48 | -1920.26 | 19.67 | 0.08 |
| S + Diel + Herd + Calf + Human + S*Calf + Wolf_UDy_ + Wolf_Dcat_ + Wolf_UDy_*Wolf_Dcat_ | -2.42 | 2.04 | -1920.92 | 19.85 | 0.04 |
| S + Diel + Herd + Calf + Human + S*Calf + Wolf_UDy_ | -2.59 | 2.79 | -1921.09 | 19.66 | 0.04 |
| S + Diel + Herd + Calf + Human + S*Calf + Lion kill | -2.79 | 2.84 | -1921.29 | 19.63 | 0.03 |
| S + Diel + Herd + Calf + Human + S*Calf + Wolf kill | -3.17 | 2.94 | -1921.67 | 19.65 | 0.02 |
| S + Diel + Herd + Calf + Human + S*Calf | -3.95 | 3.24 | -1922.45 | 19.52 | 0.01 |
| S + Diel + Herd + Calf + Human + S*Calf + Wolf_D_ | -4.14 | 3.18 | -1922.64 | 19.58 | 0.01 |
| S + Diel + Herd + Calf + Human | -4.46 | 3.75 | -1922.96 | 19.34 | 0.01 |
| S + Diel + Herd + Calf + NDVI + Human | -4.83 | 3.73 | -1923.33 | 19.39 | 0.00 |
| S + Diel + Herd + Calf | -5.18 | 3.94 | -1923.68 | 19.23 | 0.00 |
| S + Diel + Herd + Calf + Human + S*Human | -6.02 | 3.83 | -1924.52 | 19.48 | 0.00 |
| S + Diel + Herd + Calf + S*Herd | -7.35 | 3.95 | -1925.84 | 19.38 | 0.00 |
| S + Diel + NDVI | -7.76 | 4.67 | -1926.26 | 19.00 | 0.00 |
| Herd + Calf + Human | -10.12 | 5.55 | -1928.62 | 18.76 | 0.00 |
| Null | -12.35 | 6.13 | -1930.85 | 18.39 | 0.00 |

Model terms: S = season; Diel = diel period; Herd = herd size; Calf = calf:cow ratio; Human = human density; Wolf_UDy_ = yearly Mexican wolf pack UD; Wolf_Dcat_ = categorical Mexican wolf distance (1 = collared wolf was within 3 km in past 24 hours, 0 = no collared wolf was within 3 km in past 24 hours); Lion encounter = mountain lion HSF weighted by global population UD; Lion kill = mountain lion risky places model weighted by global population UD; Wolf kill = Mexican wolf risky places model weighted by yearly population UD; Wolf_D­­_ = distance to nearest collared Mexican wolf in the past 24 hours; NDVI = normalized difference vegetation index.

Table F8. Model comparison for overall herd behavior. Model performance was assessed using Pareto-Smoothed Importance Sampling-Leave One Out cross validation, and models with standard error differences of the Expected Log Pointwise Predictive Density score (ΔSE ELPD) larger than the ΔELPD were considered competing (unshaded in table). Model weights (*w*) were estimated using Pseudo-Bayesian model averaging. Human density was universally uninformative and was removed from all model structures. Predation risk metrics are shown in bold.

| Model | ΔELPD | ΔSE | ELPD | SE | *w* |
| --- | --- | --- | --- | --- | --- |
| S + Diel + Herd + Calf + R3_D_ + Wolf encounter + Wolf_D_ | 0.00 | 0.00 | 4300.15 | 80.24 | 0.62 |
| S + Diel + Herd + Calf + R3_D_ + Wolf_D_ | -0.66 | 2.48 | 4299.49 | 80.11 | 0.32 |
| S + Diel + Herd + Calf + R3_D_ | -3.05 | 9.58 | 4297.11 | 80.57 | 0.03 |
| S + Diel + Herd + Calf + R3_D_ + Wolf encounter | -3.24 | 9.27 | 4296.92 | 80.69 | 0.02 |
| S + Diel + Herd + Calf + R3_D_ + Wolf kill | -4.54 | 9.25 | 4295.61 | 80.66 | 0.01 |
| S + Diel + Herd + Calf + R3_D_ + NDVI | -5.45 | 9.77 | 4294.70 | 80.43 | 0.00 |
| S + Diel + Herd + Calf + R3_D_ + Lion encounter | -6.54 | 9.64 | 4293.61 | 80.56 | 0.00 |
| S + Diel + Herd + Calf + R3_D_ + Lion kill | -6.56 | 9.38 | 4293.59 | 80.59 | 0.00 |
| S + Diel + Herd + Calf + R3_D_ + Wolf encounter + Lion kill | -7.28 | 9.25 | 4292.87 | 80.75 | 0.00 |
| S + Diel + Herd + Calf + R3_D_ + Wolf encounter + Wolf_Dcat_ + Wolf encounter*Wolf_Dcat_ | -8.75 | 9.59 | 4291.40 | 80.74 | 0.00 |
| Herd + Calf + R3_D_ | -9.09 | 12.67 | 4291.07 | 81.03 | 0.00 |
| S + Diel + Herd + Calf + R3_D_ + S*Herd | -9.92 | 11.58 | 4290.23 | 80.97 | 0.00 |
| S + Diel + NDVI | -11.41 | 12.80 | 4288.74 | 79.12 | 0.00 |
| S + Diel + Herd + Calf | -11.70 | 11.24 | 4288.45 | 79.94 | 0.00 |
| S + Diel + Herd + Calf + R3_D_ + Human + S*Human | -12.56 | 12.41 | 4287.59 | 81.60 | 0.00 |
| S + Diel + Herd + Calf + S*Herd | -19.69 | 12.86 | 4280.46 | 80.28 | 0.00 |
| Null | -20.86 | 15.26 | 4279.30 | 79.82 | 0.00 |

Model terms: S = season; Diel = diel period; Herd = herd size; Calf = calf:cow ratio; R3_D_ = distance to unmaintained roads; Wolf encounter = Mexican wolf HSF weighted by global pack UD and average pack sizes; Wolf_D­­_ = distance to nearest collared Mexican wolf in the past 24 hours; Wolf kill = Mexican wolf risky places model weighted by global pack UD and average pack sizes; NDVI = normalized difference vegetation index; Lion encounter = unweighted mountain lion HSF; Lion kill = unweighted mountain lion risky places model; Wolf_Dcat_ = categorical Mexican wolf distance (1 = collared wolf was within 3 km in past 24 hours, 0 = no collared wolf was within 3 km in past 24 hours); Human = human density.

*Figures*

**A**


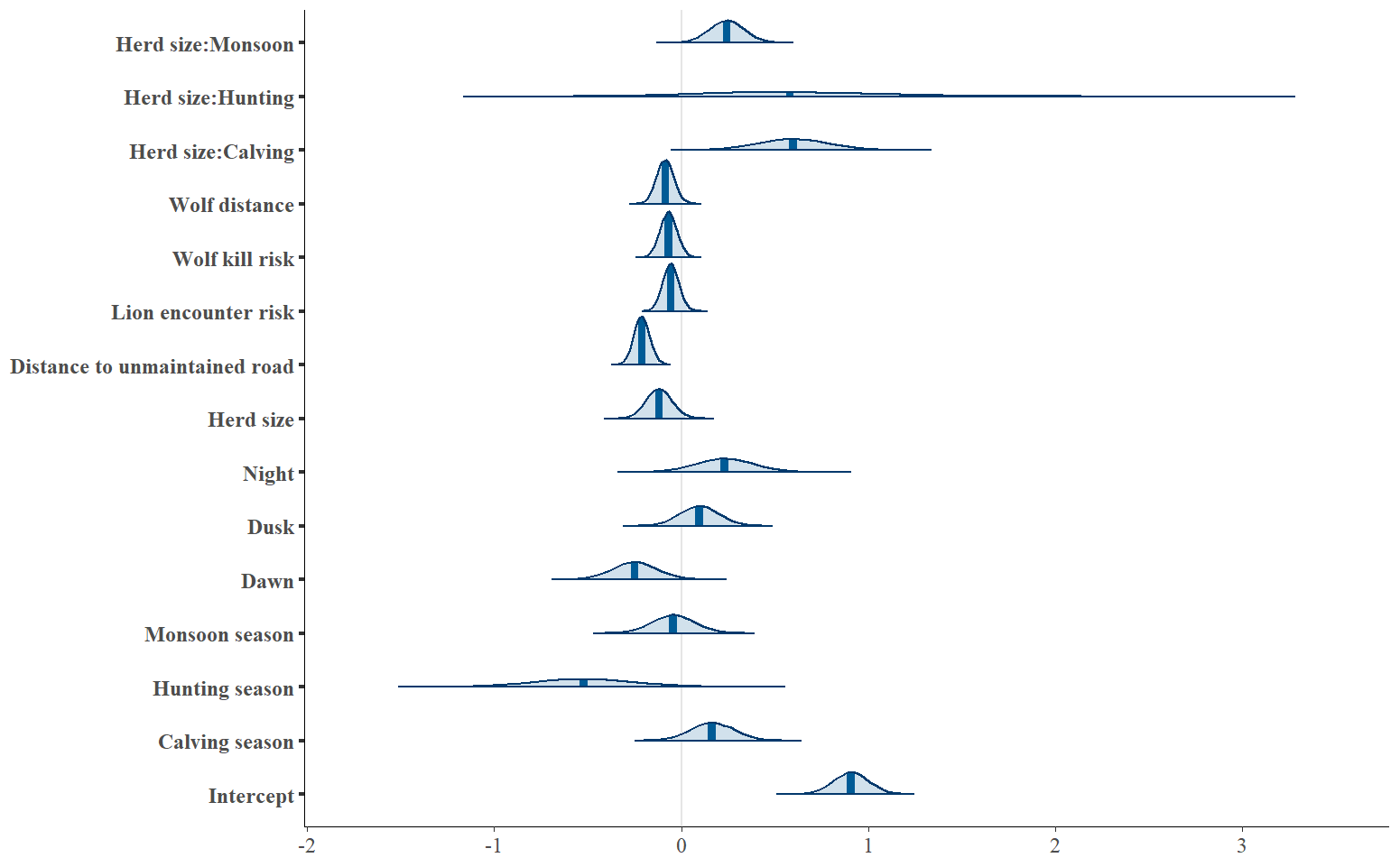

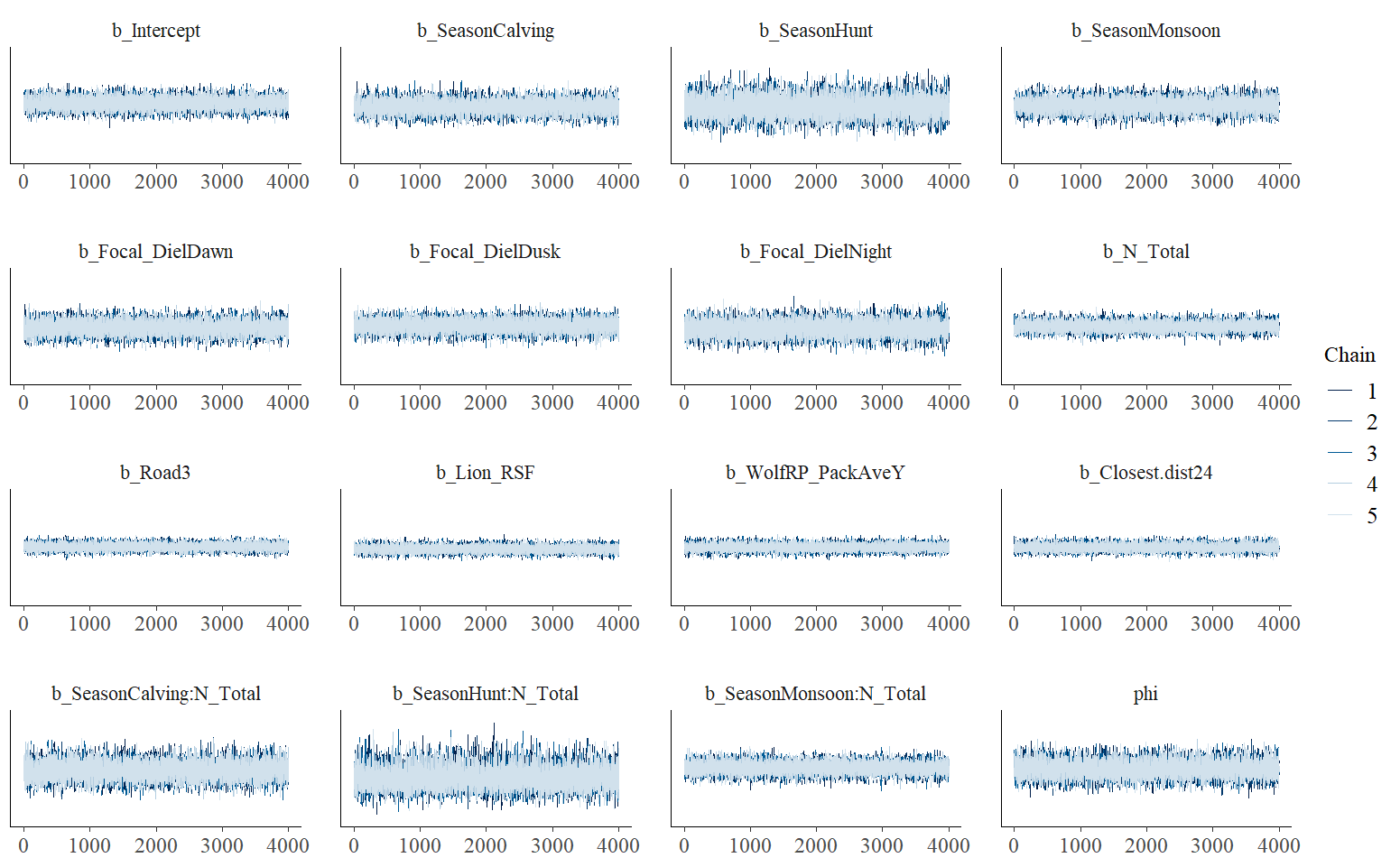


**B**

Figure F1. Posterior density plots for parameter estimates (A) and trace plots of MCMC chain mixing (B) from the top ranked individual proportion foraging model of elk in east-central Arizona and west-central New Mexico between 2022-2023. The 95% credible intervals are shown as light-blue shaded portions of posterior density plots.


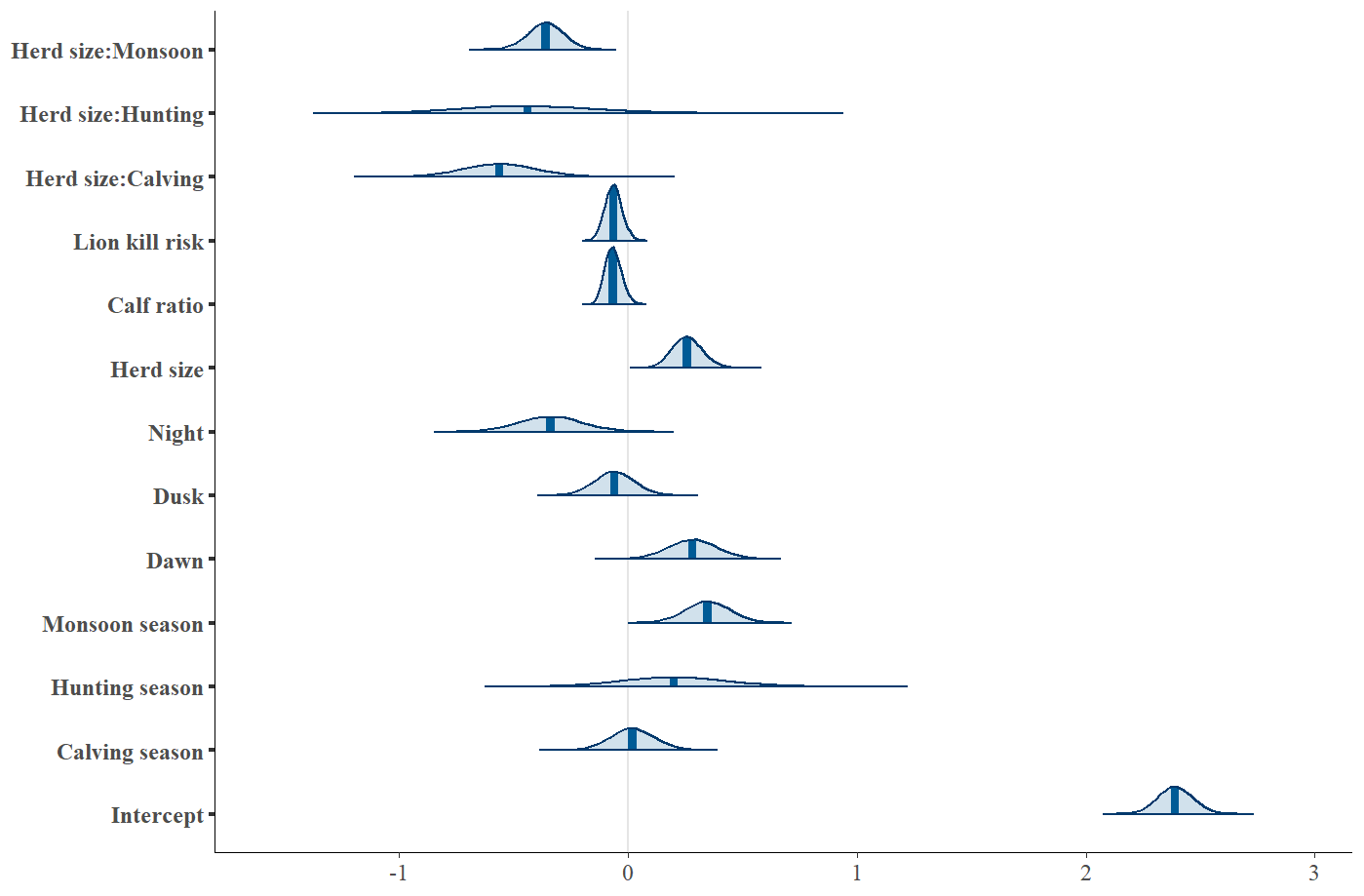


**A**

**B**


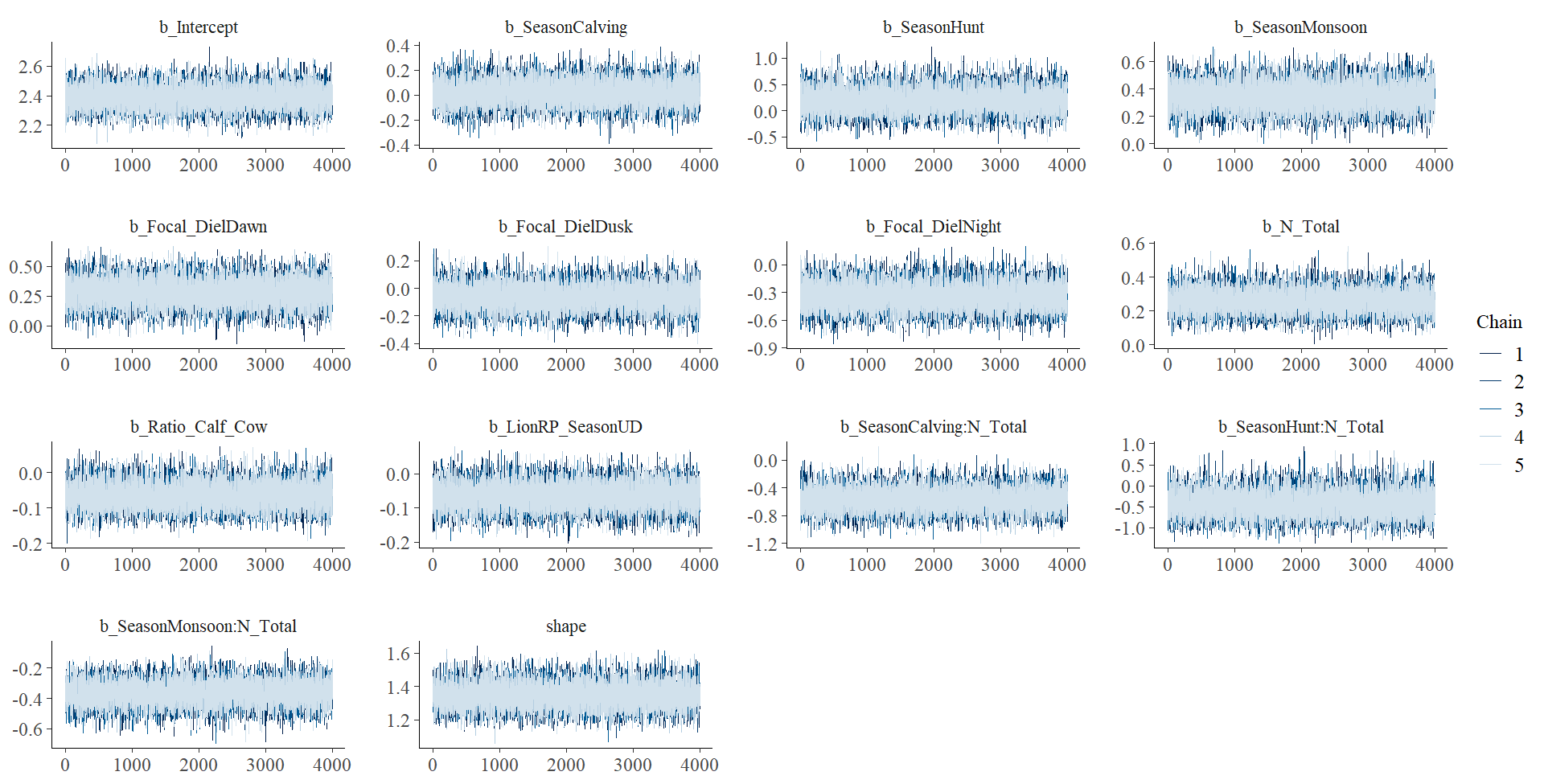


Figure F2. Posterior density plots for parameter estimates (A) and trace plots of MCMC chain mixing (B) from the top ranked SPM model of elk in east-central Arizona and west-central New Mexico between 2022-2023. The 95% credible intervals are shown as light-blue shaded portions of posterior density plots.


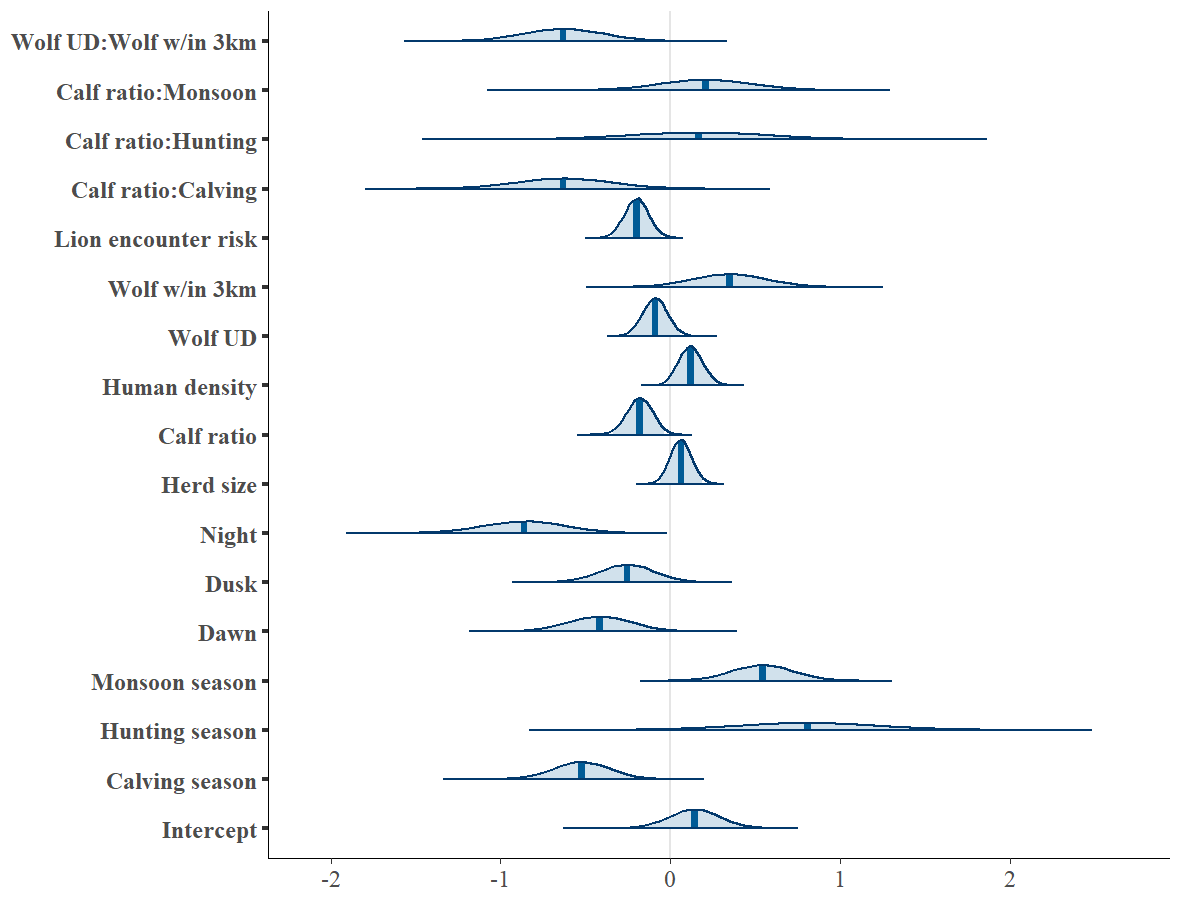


**B**

**A**


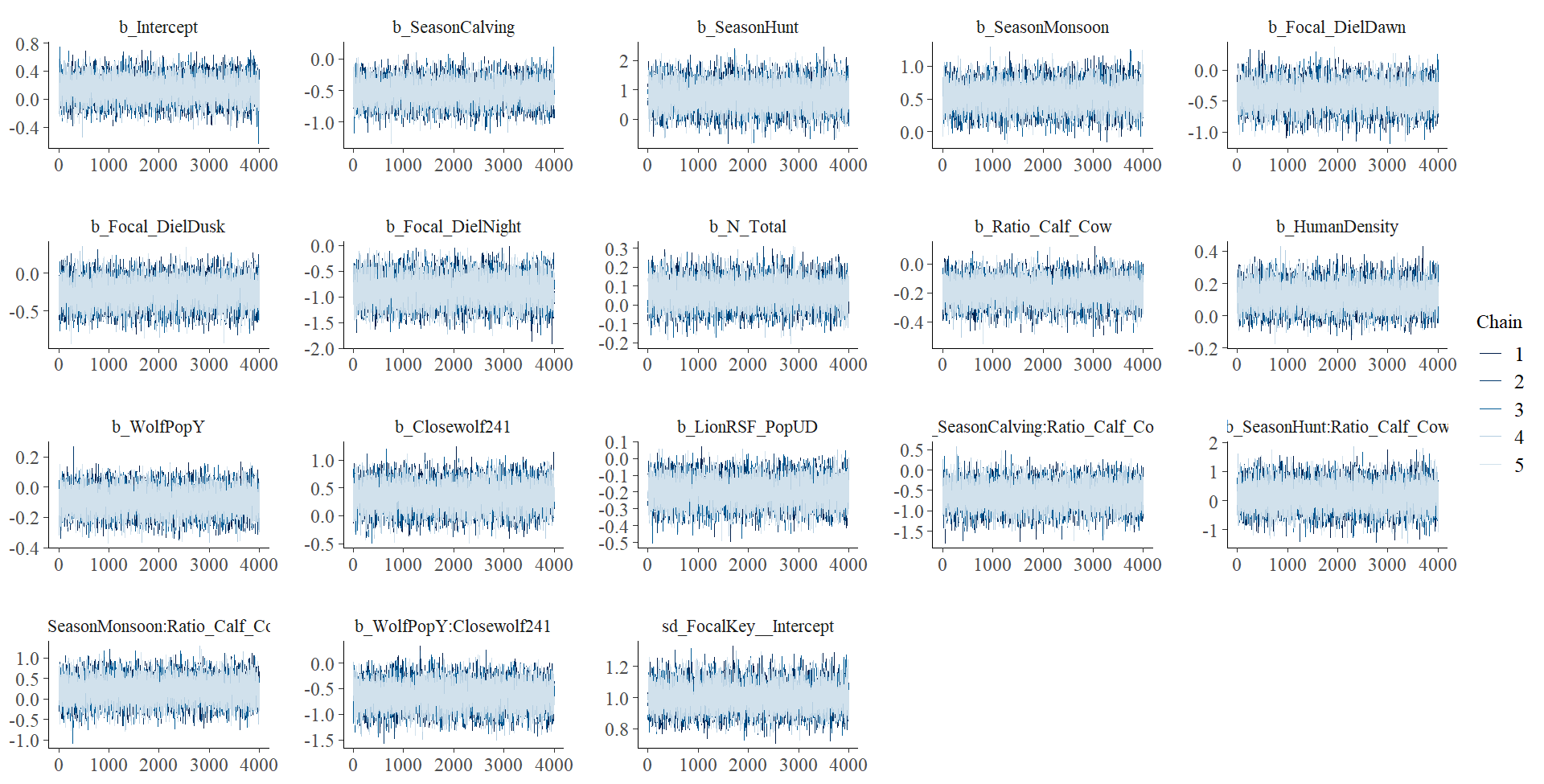


Figure F3. Posterior density plots for parameter estimates (A) and trace plots of MCMC chain mixing (B) from the top ranked multitasking probability model of elk in east-central Arizona and west-central New Mexico between 2022-2023. The 95% credible intervals are shown as light-blue shaded portions of posterior density plots.


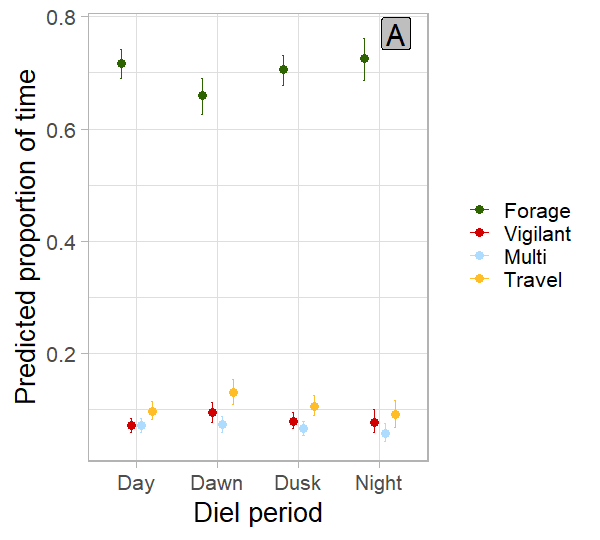

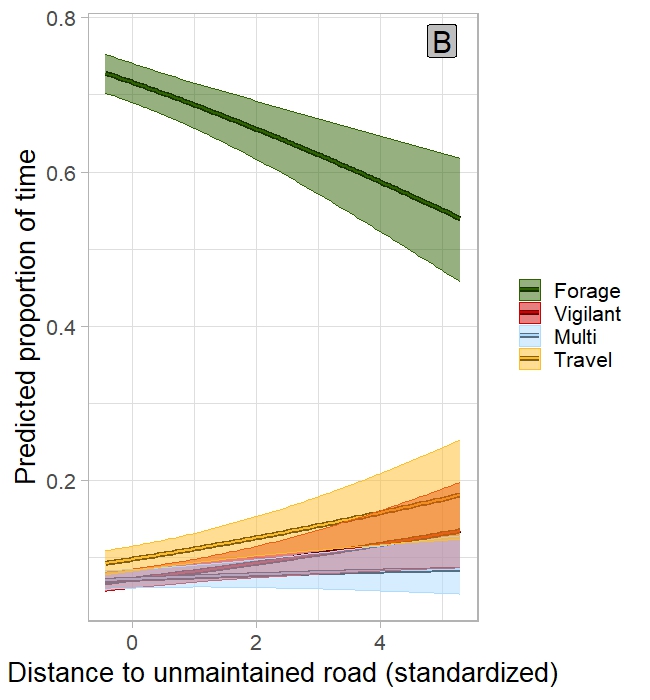

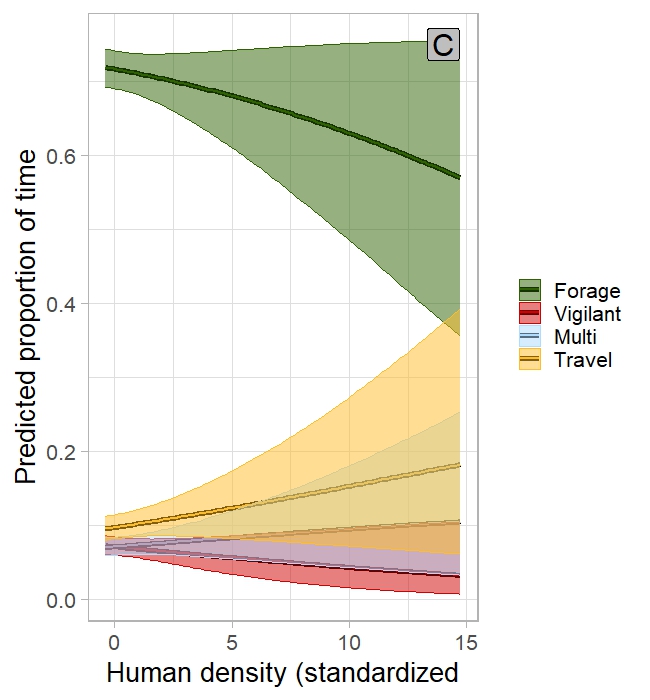

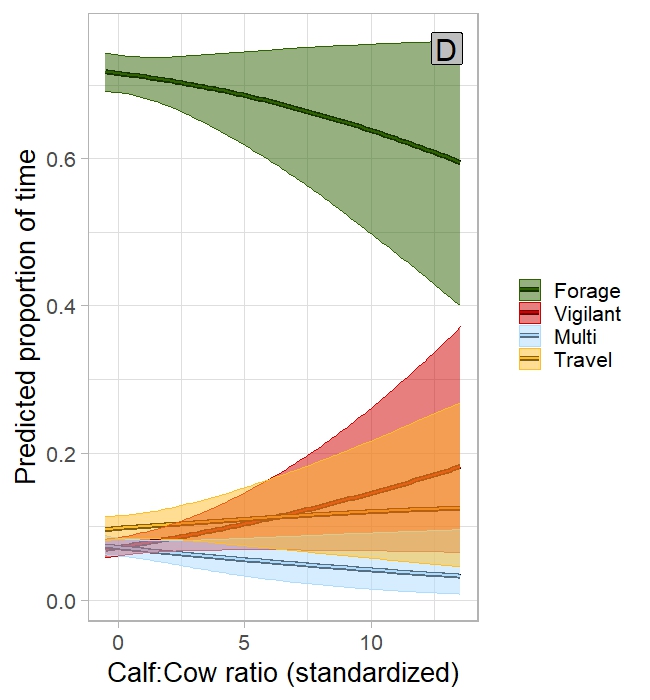


Figure F4. Effects of (A) diel period, (B) distance to unmaintained road, (C) human density, and (D) calf:cow ratio on the predicted proportions of time individual elk spent foraging, intensely vigilant, multitasking, and traveling during foraging bouts in east-central Arizona and west-central New Mexico between 2022-2023 with 95% credible intervals. “Other” behaviors were modeled but excluded from plots for clarity.


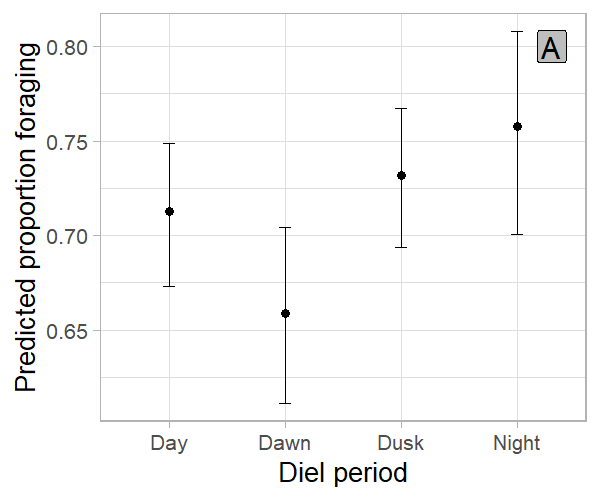

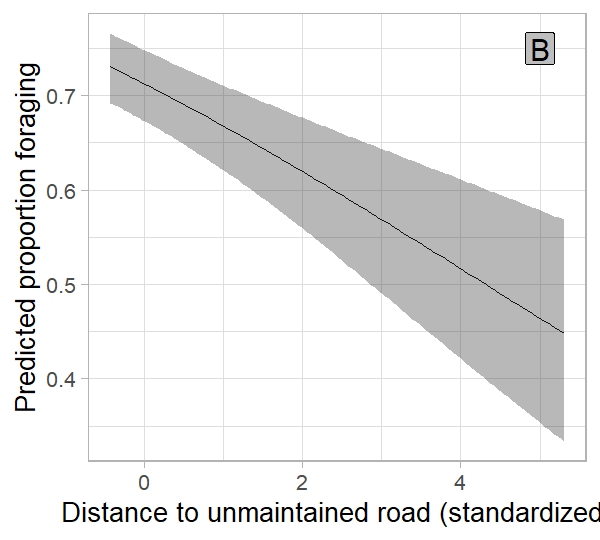


Figure F5. Effects of (A) diel period, and (B) distance to unmaintained road on the predicted proportion of time an individual elk spent foraging during an active foraging bout in east-central Arizona and west-central New Mexico between 2022-2023 with 95% credible intervals.


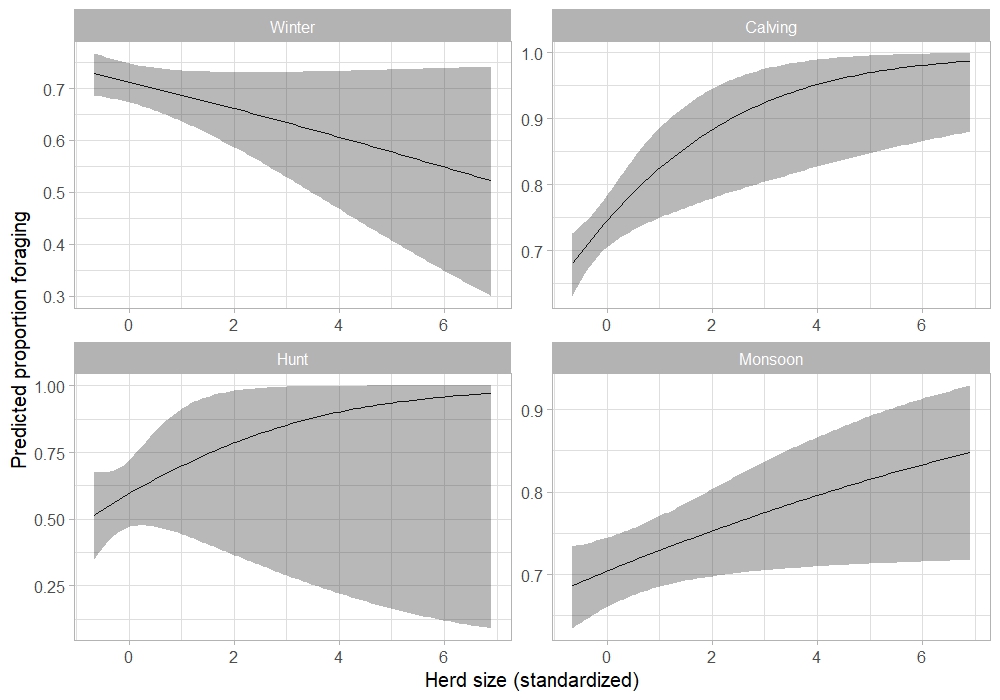


Figure F6. Effect of herd size, conditional on season, on the predicted proportion of time an individual elk spent foraging during a foraging bout in east-central Arizona and west-central New Mexico between 2022-2023 with 95% credible intervals. Winter was defined as January to April, calving season as May to June, monsoon season as July to August, and hunting season as September to December.


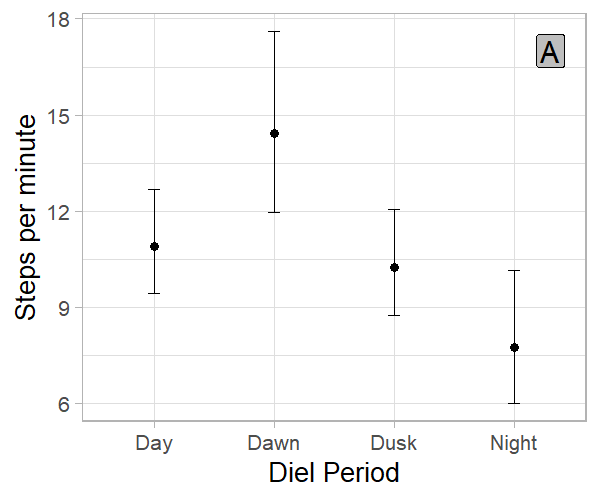

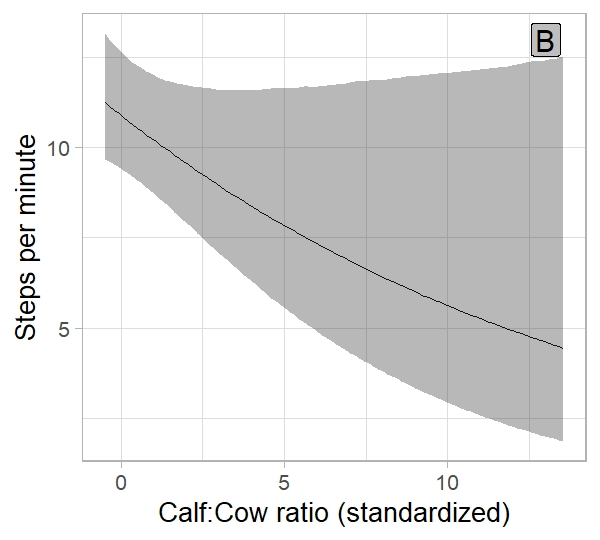


Figure F7. Effects of (A) diel period and (B) the calf:cow ratio in the herd on the predicted number of steps individual elk took per minute while foraging in east-central Arizona and west-central New Mexico between 2022-2023 with 95% credible intervals.


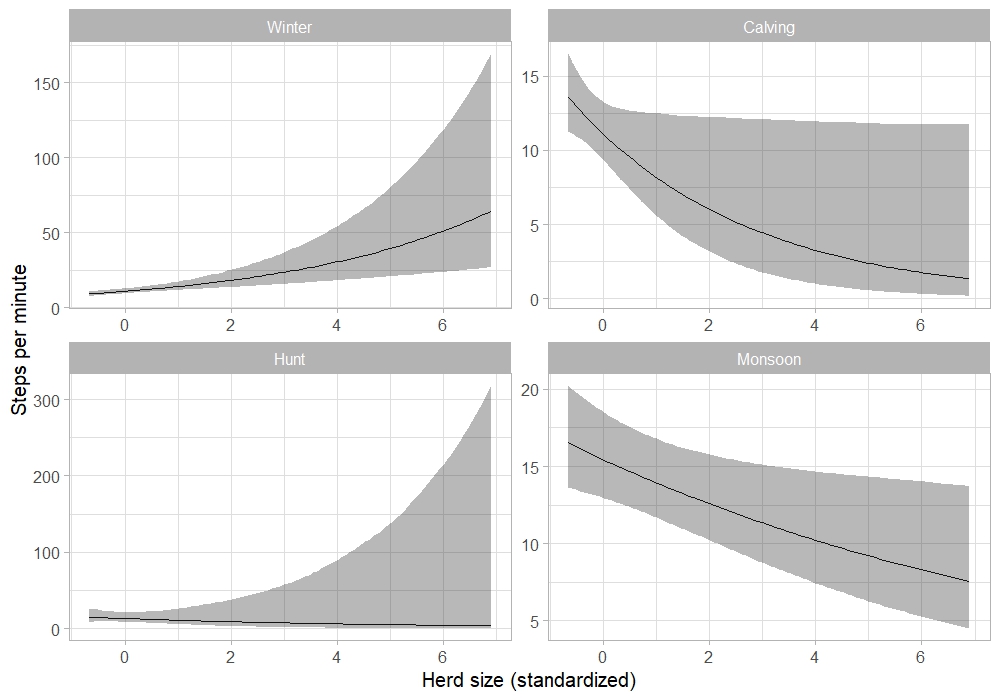


Figure F8. Effect of herd size on the predicted step rate of individual elk while actively foraging, conditional on season in east-central Arizona and west-central New Mexico between 2022-2023 with 95% credible intervals. Winter was defined as January to April, calving season as May to June, monsoon season as July to August, and hunting season as September to December.


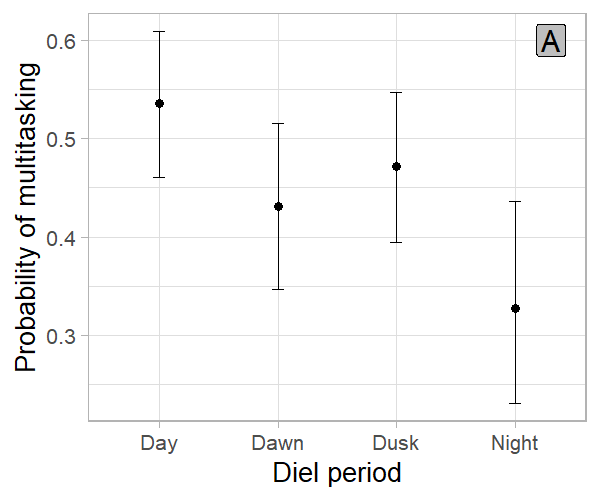

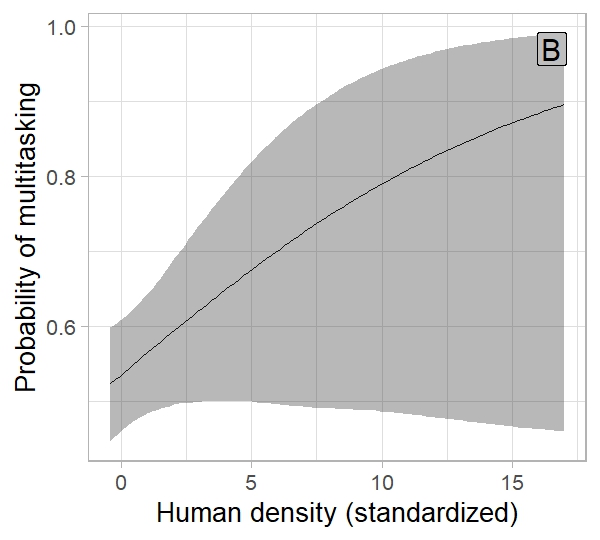

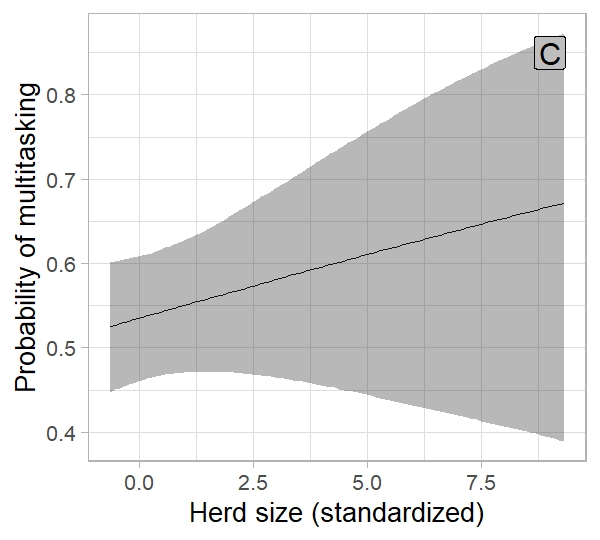


Figure F9. Effects of (A) diel period, (B) human population density, and (C) herd size on the probability of multitasking versus vigilance for individual elk in east-central Arizona and west-central New Mexico between 2022-2023 with 95% credible intervals.


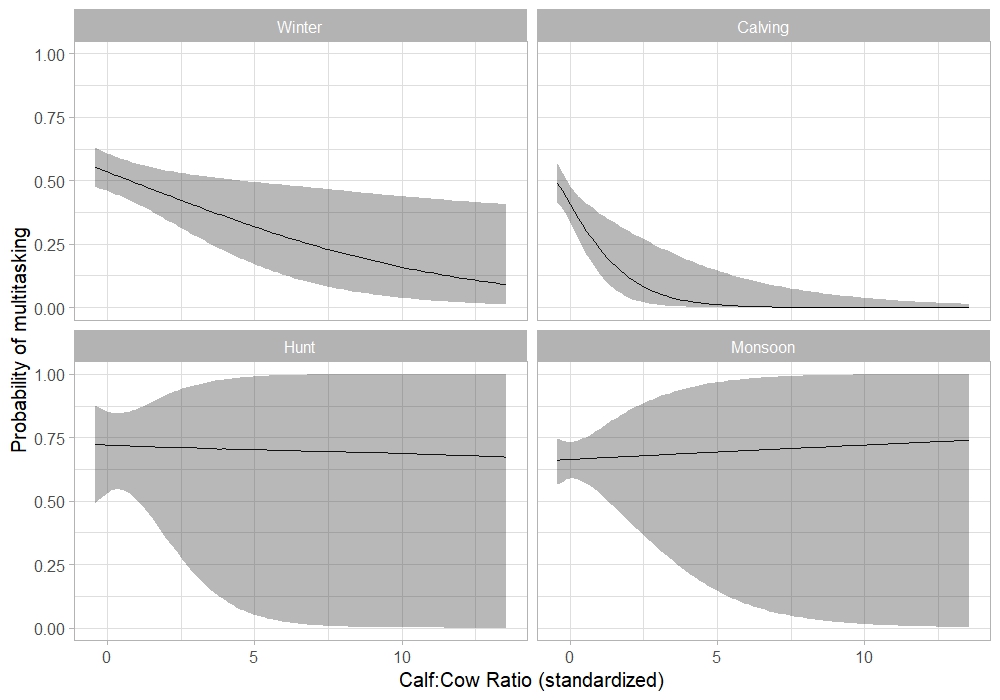


Figure F10. Effect of herd size on the probability of multitasking for individual elk, conditional on season in east-central Arizona and west-central New Mexico between 2022-2023 with 95% credible intervals. Winter was defined as January to April, calving season as May to June, monsoon season as July to August, and hunting season as September to December.


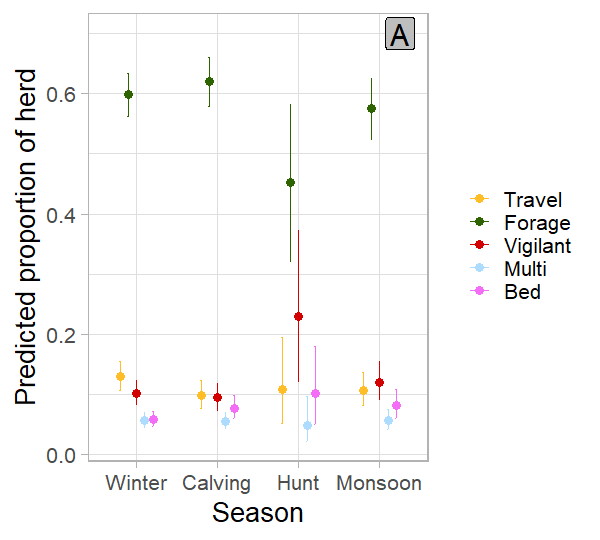

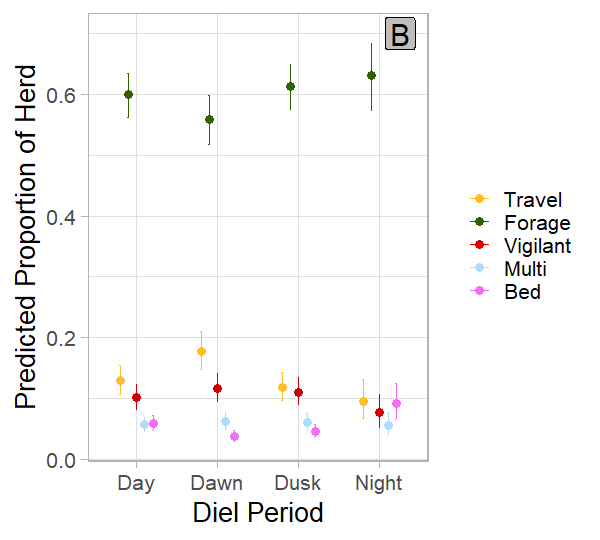

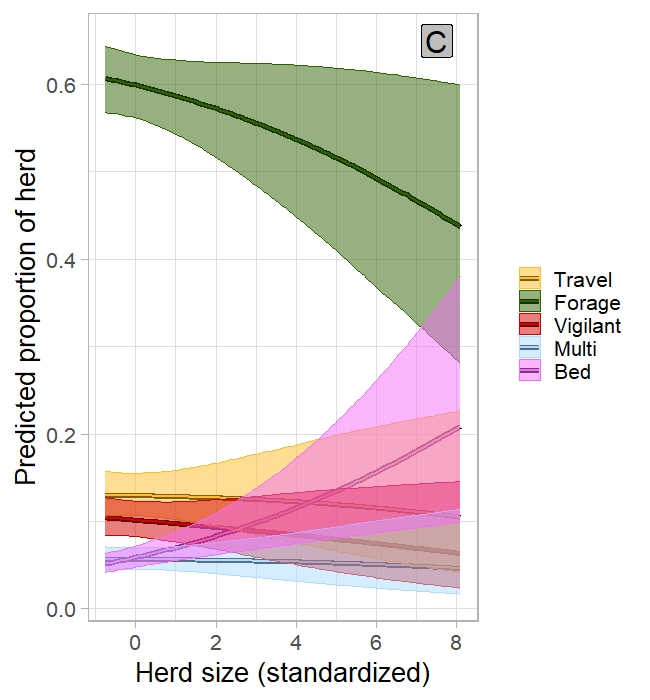

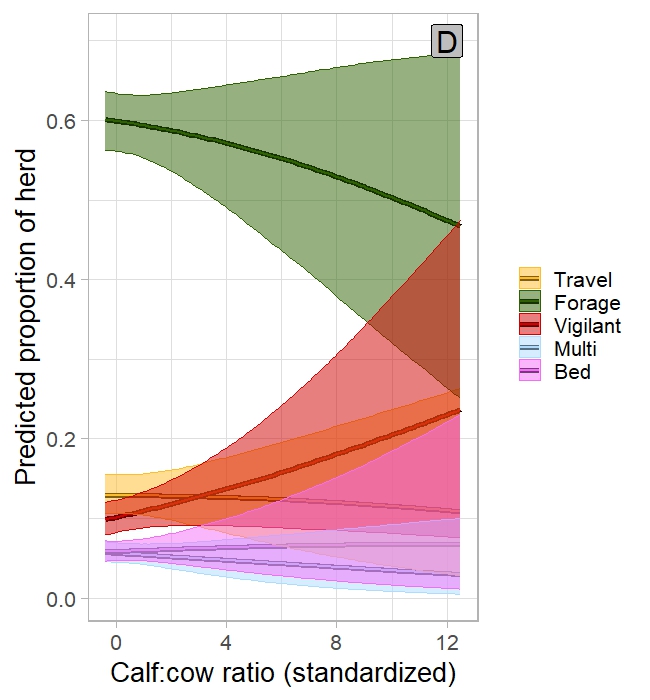


Figure F11. Effects of (A) season, (B) diel period, (C) herd size, and (D) calf:cow ratio on the predicted average proportion of an elk herd engaged in foraging, intense vigilance, multitasking, traveling, and bedded behaviors in east-central Arizona and west-central New Mexico between 2022-2023 with 95% credible intervals. “Other” behaviors were modeled but excluded from plots for clarity. Winter was defined as January to April, calving season as May to June, monsoon season as July to August, and hunting season as September to December.


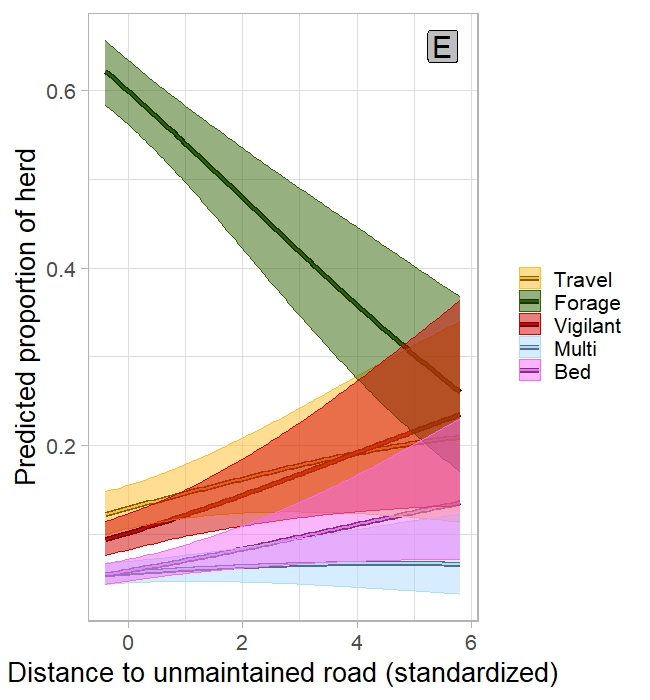


Figure F11 (*cont*.). (E) Effect of distance to unmaintained road on the predicted average proportion of an elk herd engaged in foraging, intense vigilance, multitasking, traveling, and bedded behaviors in east-central Arizona and west-central New Mexico between 2022-2023 with 95% credible intervals. “Other” behaviors were modeled but excluded from plots for clarity.
